# Structure–Activity Relationship Study on Ligands Activating the Voltage‐Gated Potassium Channel K_V_7.1

**DOI:** 10.1002/ardp.70268

**Published:** 2026-06-05

**Authors:** Florian Roßner, Judith Schmidt, Guiscard Seebohm, Bernhard Wünsch

**Affiliations:** ^1^ GRK 2515, Chemical Biology of Ion Channels (Chembion) Universität Münster Münster Germany; ^2^ Institute of Pharmaceutical and Medicinal Chemistry University of Münster Münster Germany; ^3^ Department of Cardiovascular Medicine, Institute for Genetics of Heart Diseases (IfGH) University Hospital Münster Münster Germany

**Keywords:** 1,4‐benzodiazepin‐2‐ones, activation, chiral HPLC, K_V_7.1 ion channel, lipophilicity, metabolic stability, pharmacokinetic parameters, structure–activity relationships, Sugasawa reaction, two‐electrode voltage clamp

## Abstract

K_V_7.1 agonists are promising therapeutic agents for the treatment of hypertension, arrhythmia and preterm labor. The 3‐(indol‐3‐ylmethyl) substituted 1,4‐benzodiazepine (R)‐L3 has been reported as a potent K_V_7.1 activator. In this study, (R)‐L3 was systematically modified at eight positions including the configuration at 3‐position. For the synthesis of the designed 1,4‐benzodiazepines **8** and **12**, 2‐aminobenzophenones **4** were reacted with amino acid derived 1,3‐oxazolidinediones **7** or **11**. The required 2‐aminobenzophenones **4** were obtained regioselectively by Sugasawa reaction of anilines **2** and **3** with various substituted benzonitriles, using BCl_3_ and AlCl_3_. Racemization during deprotonation and alkylation of secondary lactams **8** and **12** was not observed. However, racemization occurred during conversion of lactam **8b** into thiolactam **17**. The enantiomers of thiolactam *rac*‐**17** and triazole *rac*‐**18** were separated by chiral HPLC. The ion channel modulatory activity of the resulting 1,4‐benzodiazepines was evaluated by two‐electrode voltage‐clamp (TEVC) experiments. These TEVC experiments showed that (3*R*)‐configuration and the indolylmethyl moiety at 3‐position are essential for K_V_7.1 activation. High activity was observed for secondary lactam **8b** and methylated lactam **9b**, but larger N‐substituents reduced the channel activation. The thioamide (*R*)‐**17** and the triazole (*R*)‐**18** did not significantly activate the K_V_7.1 channel. Replacement of the F‐atom in 2‐position of the 5‐phenyl moiety by a proton or other halogen atoms reduced the K_V_7.1 activity. However, the 9‐hydroxy derivative **9i** appeared to exhibit higher agonistic activity (+68% activity increase at 1 µM) than (R)‐L3 (**9b**, +45% at 1 µM). Moreover, higher phase I metabolic stability was observed for phenol **9i**.

## Graphical abstract

Activation of the voltage gated K^+^ channel K_V_7.1 leads to hyperpolarization shift of cell membranes. In a thorough SAR study eight positions of the lead compound (R)‐L3 were modified. (*R*)‐configuration, the indolylmethyl moiety in 3‐position and the 2‐fluorophenyl moiety in 5‐position appeared to be essential for high K_V_7.1 activation, whereas an OH moiety in 9‐position increased the agonistic activity.

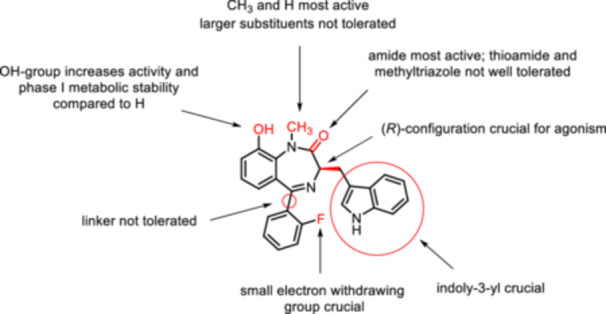

**Abbreviations**
ee: enantiomeric excess
HPAC: high performance affinity chromatography
HPLC: high performance liquid chromatography
LQTS1: long QT syndrom 1
MOPS: 3‐
  morpholinopropanesulfonic acid
NADPH: Nicotinamide‐Adenine‐Dinucleotide‐Phosphate‐hydrogen
PPB: plasma protein binding
SEM: standard error of mean
TEVC: two‐electrode voltage clamp

## 1 Introduction

**Figure 1:**
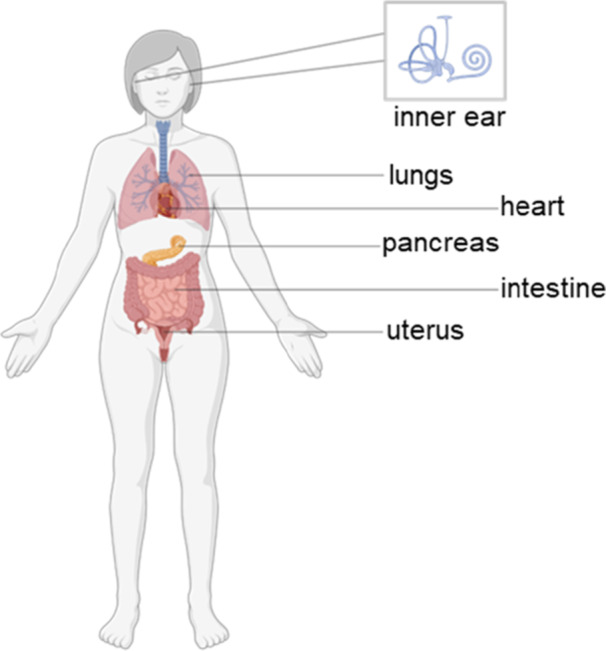
Distribution of K_V_7.1 in the human body. Created in https://BioRender.com.

The voltage‐gated potassium channel K_V_7.1 is formed by the assembly of four identical subunits (homotetramer) encoded by the *KCNQ1* gene [1]. Upon depolarization of the cell, the channel opens and allows an outflow of K^+^ ions, which subsequently leads to a repolarization of the cell [2]. It is expressed ubiquitously in the human body and is therefore involved in several (patho)physiological processes. Organs with highly relevant K_V_7.1 physiological functions include heart, lungs, inner ear, pancreas, intestine and uterus, but are not limited to these organs (Figure 1) [3].

Historically, the ion channel is best known for its critical role in the heart. In cardiac myocytes, the K_V_7.1‐homotetramer can co‐assemble with the KCNE1 auxiliary subunit (minK, IsK) to form the slow delayed rectifier current I_Ks_ and thus regulates the action potential duration. K_V_7.1 dysfunction due to loss‐of‐function mutations leads to long QT syndrome type 1 (LQTS1), which can cause life‐threatening arrhythmia. Due to this important function, the channel was originally termed K_V_LQT1 [4, 5].

However, more recent data on K_V_7.1 expression in the human body provided the basis for the discovery of its various roles in different (patho)physiological processes. Activation of K_V_7.1 channels has been shown to cause a significant vasodilatation of preconstricted rat mesenteric arteries [6]. The activation of the channel causes a hyperpolarization of the cell and therefore reduces the activity of voltage‐dependent Ca^2+^ channels, which open upon depolarization. The reduced Ca^2+^ influx subsequently induces a relaxation of vascular smooth muscles [7]. This effect on vascular smooth muscles suggests a potential use of K_V_7.1 activators for the treatment of hypertension.

Similar to the observed relaxation of vascular smooth muscles, K_V_7 activators have been shown to suppress myometrial contractions and therefore uphold promise as a novel tocolytic treatment approach for preterm labor [8]. In fact, the lead compound (R)‐L3 of this manuscript was already mentioned in a patent by MSD as the most preferred compound for the treatment of preterm labor [9].

In addition, the robust link between polymorphisms of the gene *KCNQ1* and type 2 diabetes suggests a vital role of the K_V_7.1 channel in insulin secretion [10, 11, 12]. In fact, it is known that patients with *KCNQ1* loss‐of‐function mutations suffer from hyperinsulinemia and hypoglycemia [13]. Because an opening of voltage‐dependent Ca^2+^ channels is crucial for insulin secretion, a possible explanation for the observed link between K_V_7.1 and insulin release might be that an opening of the K_V_7.1 ion channels reduces the open state probability of voltage‐dependent Ca^2+^ channels (similar to what is observed in smooth muscle cells) and therefore inhibits insulin secretion [14, 15].

In summary, K_V_7.1 channel activators are of high interest for pharmacological research and for the potential treatment of diseases such as cardiac arrhythmia, hypertension, preterm labor and hyperinsulinemia.

**Figure 2:**
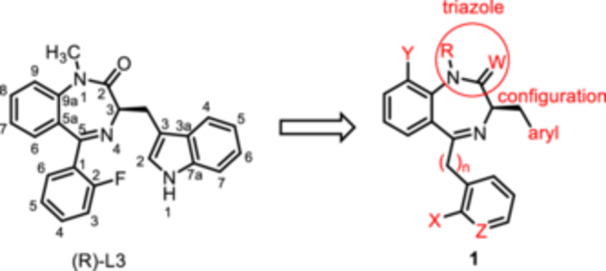
Lead compound (R)‐L3 and envisaged structural modifications summarized in general formula **1**.

For this study, (R)‐L3 (Figure 2), initially termed L‐364,373, served as the lead compound, which was first synthesized within a set of cholecystokinin inhibitors [16]. Later, its potential to activate the K_V_7.1 channel was detected. (R)‐L3 was able to potently shorten the action potential duration in guinea pig cardiac myocytes. This property suggests a potential for the treatment of the long QT syndrome type 1 (LQTS1) [17].

Despite the high relevance of the K_V_7.1 channel in various diseases, detailed relationships between structural modifications of (R)‐L3 and K_V_7.1 channel activation have not been reported yet. Herein, the synthesis of diversely modified (R)‐L3 derivatives and their effects on K_V_7.1 channels are presented. In Figure 2, the envisaged modifications of (R)‐L3 are summarized.

## 2 Results and Discussion

### 2.1 Synthesis

Various 2‐acylated anilines **4a**–**h** should serve as building blocks for the synthesis of the desired 1,4‐benzodiazepines. However, acylation of aniline derivatives without a *p*‐substituent leads to mixtures of *o*‐ and *p*‐substituted anilines. Therefore, the Sugasawa reaction was used to introduce the acyl moiety selectively in *o*‐position of the amino moiety. In the Sugasawa reaction, anilines **2** and **3** were acylated with benzonitriles using BCl_3_ and AlCl_3_. BCl_3_ coordinates with both the N‐atom of anilines and the N‐atom of benzonitriles directing the acylation in *o*‐position [18].

**Scheme 1:**
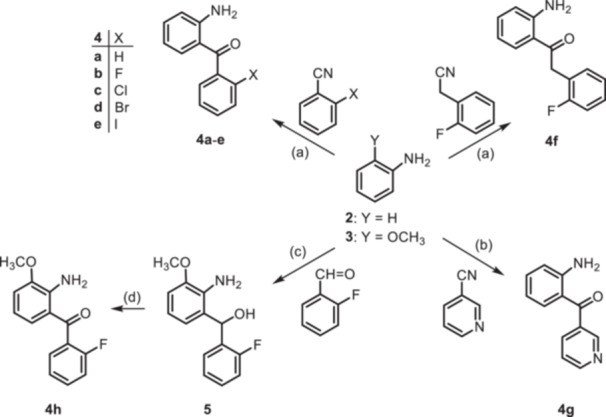
Synthesis of differently 2‐acylated anilines **4a–h**. Reagents and reaction conditions: (a) 1. BCl_3_, AlCl_3_, xylene, 0°C then 144°C, 16 h; 2. H_2_O, 100°C, 40 min, 63%–91%. (b) 1. BCl_3_, AlCl_3_, xylene, 0°C then 144°C, 6 h; 2. 3 M HCl, 100°C, 1 h, 13%. (c) 1. 2‐methoxyaniline (**3**), PhBCl_2_, NEt_3_, CH_2_Cl_2_, −20°C, 30 min; 2. 2‐fluorobenzaldehyde, CH_2_Cl_2_, rt, 5 h; 3. 2 M NaOH, THF, rt, 1 h, 53%. (d) MnO_2_, CH_2_Cl_2_, THF, 40°C, 16 h, 93%.

Standard Sugasawa reaction conditions led to acylation of aniline (**2**) with various benzonitriles as well as with homologous (2‐fluorophenyl)acetonitrile to afford the 2‐acylated anilines **4a**–**f** in 63%–91% yield. For the synthesis of pyridin‐3‐ylmethanone **4g** modified Sugasawa conditions developed by Earley et al. [19] were used employing higher amounts of BCl_3_ and AlCl_3_. To obtain the *o*‐methoxy substituted product **4h** a two‐step procedure was necessary since the strong Lewis acids BCl_3_ and AlCl_3_ used in the standard Sugasawa reaction led to cleavage of the ether. Thus, at first 2‐methoxyaniline (**3**) was reacted with PhBCl_2_ and 2‐fluorobenzaldehyde. The resulting aminoborane intermediate was hydrolyzed with 2 M NaOH to give the secondary alcohol **5** [20], which was oxidized with MnO_2_ [21] to obtain the 2‐aminobenzophenone **4 h** (Scheme 1).

**Scheme 2:**
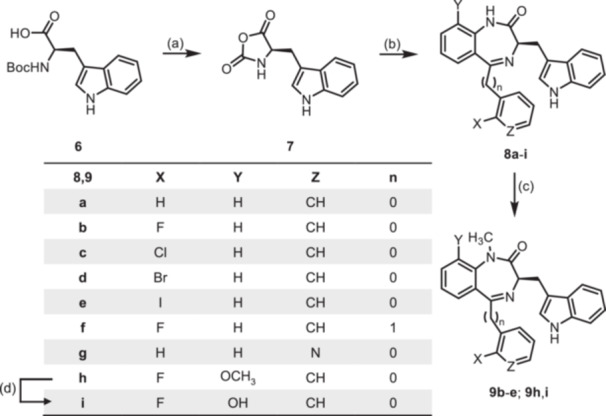
Synthesis of indol‐3‐ylmethyl substituted 1,4‐benzodiazepin‐2‐ones **8** and **9**. Reagents and reaction conditions: (a) PCl_3_, CH_2_Cl_2_, 0°C, then rt, 16 h. (b) 1. Addition of 2‐aminoketones **4a**–**h**, TFA, toluene, 60°C, 1 h; 2. Et_3_N, toluene 80°C, 1 h, 7%–53%. (c) 1. NaH, DMF, 0°C, 1 h; 2. CH_3_I, rt, 1 h, 42%–89%. (d) BBr_3_, CH_2_Cl_2_, rt, 3 h, 65% (**8i**), 55% (**9i**). The (*S*)‐configured enantiomers *ent*‐**8b** and *ent*‐**9b** were prepared analogously starting with Boc‐protected (*S*)‐configured tryptophan (*ent*‐**6**).

Since it has been reported that the synthesis of 3‐substituted 1,4‐benzodiazepin‐2‐ones is susceptible to racemization when using Boc or Fmoc protected amino acids [22] or amino acid esters [23], the oxazolidinedione method reported by Fier and Whittaker [24] was used to obtain the 1,4‐benzodiazepines **8** and **12**. According to this method, Boc protected (*R*)‐configured tryptophan (**6**) was treated with PCl_3_ to obtain the 1,3‐oxazolidine‐2,5‐dione **7** as an intermediate [25]. Without purification, oxazolidinedione **7** was reacted with the 2‐acylated anilines **4a**–**h** to provide 1,4‐benzodiazepines **8a**–**h** in 7%–67% yield. Deprotonation with NaH and subsequent reaction with CH_3_I [16] converted the secondary lactams **8** into tertiary lactams **9**. Cleavage of the methyl ether of **8h** and **9h** with BBr_3_ led to the phenols **8i** and **9i**. The enantiomers *ent*‐**8b** and *ent*‐**9b** were prepared starting with Boc‐protected (*S*)‐configured tryptophan (*ent*‐**6**) (Scheme 2).

**Scheme 3:**
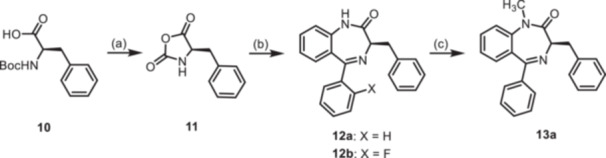
Synthesis of benzyl substituted 1,4‐benzodiazepin‐2‐ones **12** and **13**. Reagents and reaction conditions: (a) PCl_3_, CH_2_Cl_2_, 0°C, then rt, 16 h. (b) 1. Addition of 2‐aminoketones **4a** and **4b**, TFA, toluene, 60°C, 1 h; 2. Et_3_N, toluene 80°C, 1 h, 52%–67%. (c) 1. NaH, DMF, 0°C, 1 h; 2. CH_3_I, rt, 1 h, 73%. The (*S*)‐configured enantiomers *ent*‐**12a** and *ent*‐**13a** were prepared analogously starting with Boc‐protected (*S*)‐configured phenylalanine (*ent*‐**10**).

The 3‐benzyl substituted 1,4‐benzodiazepines **12** were prepared analogously. Boc protected (*R*)‐phenylalanine (**10**) was converted into the oxazolidinedione **11** upon treatment with PCl_3_. Reaction of intermediate **11** with 2‐aminobenzophenones **4a** and **4b** provided 1,4‐benzodiazepines **12a** and **12b**. Methylation of secondary lactam **12a** led to the methylated 1,4‐benzodiazepine **13a**. Starting the reaction sequence with Boc‐protected (*S*)‐configured phenylalanine (*ent*‐**10**) led to the enantiomers *ent*‐**12a** and *ent*‐**13a** (Scheme 3).

**Scheme 4:**
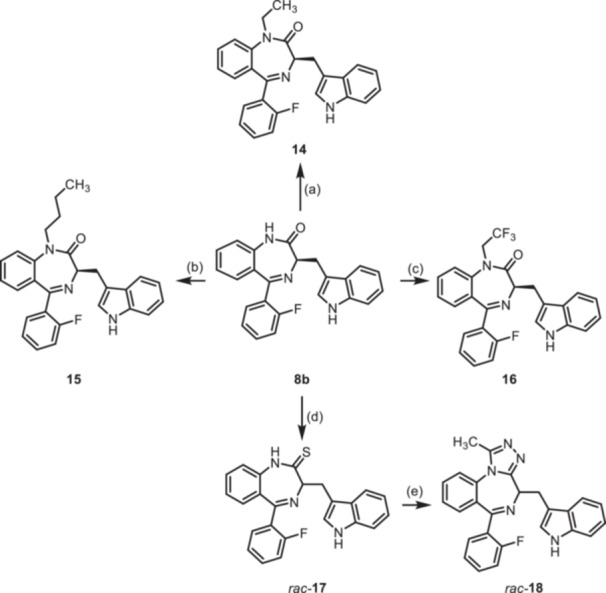
Synthesis of 1,4‐benzodiazepine‐2‐ones **14**–**16** with various alky substituents in 1‐position, thioamide *rac*‐**17** as well as triazole *rac*‐**18**. Reagents and reaction conditions: (a) 1. NaH, DMF, 0°C, 1 h; 2. ethyl methanesulfonate, 40°C, 1 h, 82%. (b) 1. NaH, DMF, 0°C, 1 h; 2. butyl methanesulfonate, 40°C, 1 h, 54%. (c) 1. NaH, DMF, 0°C, 1 h; 2. trifluoroethyl trifluoromethanesulfonate, rt, 40 min, 91% (d) Lawessons reagent, THF, reflux, 16 h, 73%. (e) AcNHNH_2_, butan‐1‐ol, reflux, 16 h, 35%.

To further expand the diversity of the substitution pattern, further alkyl groups were introduced in 1‐position of 1,4‐benzodiazpine **8b**. After deprotonation of secondary lactam **8b** with NaH, alkylation with ethyl and butyl methanesulfonate provided the ethyl and butyl derivatives **14** and **15**, whereas the trifluoroethyl moiety of **16** was introduced by alkyation with the corresponding trifluoromethanesulfonate (Scheme 4). The transformation of the amide **8b** into the thioamide *rac‐* **17** was achieved with Lawesson's reagent. During this transformation racemization of the product was observed, which is in accordance with literature reports [26]. Reaction of racemic thioamide *rac‐* **17** with acetohydrazide [27] led to the 1,2,4‐triazole *rac‐* **18** in 35% yield. (Scheme 4).

### 2.2 Evaluation of Enantiopurity of 9b and Separation of Enantiomers of 17 and 18

**Figure 3:**
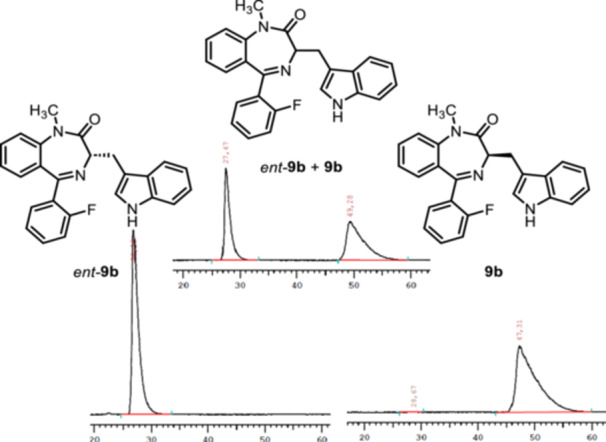
Chiral HPLC chromatograms of **9b** and e*nt*‐**9b**. Top: racemic mixture of **9b** and *ent*‐**9b**; bottom left: *ent‐* **9b**; bottom right: **9b**. HPLC conditions: column: Daicel Chiralpak IA (l = 250 mm, d = 4.6 mm); eluent: *iso*‐hexane/*iso*‐propanol = 90:10; flow rate: 1.0 mL/min; detection λ = 254 nm. *ent‐* **9b**: 100.0% ee; **9b**: 99.9% ee.

To prove that the synthesis of 1,4‐benzodiazepins **8** and their subsequent alkylation did not lead to racemization, a racemic mixture of **9b** (lead compound (R)‐L3) and its enantiomer *ent‐* **9b** was prepared and analyzed by chiral HPLC. Subsequently, the pure enantiomers **9b** and *ent*‐**9b** were measured separately using the established separation method (Figure 3). These experiments resulted in an enantiomeric excess of 100.0% for e*nt‐* **9b** and 99.9% for **9b**. These experiments led to the conclusion that the synthesis of 1,4‐benzodiazepines **8** and their subsequent alkylation did not lead to racemization.

**Figure 4:**
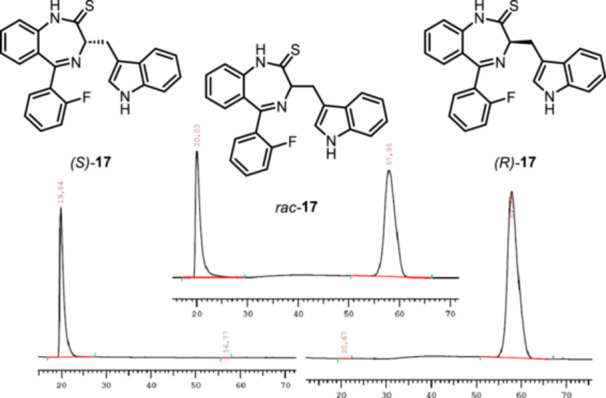
Chiral HPLC chromatograms of thioamides **17**. Top: racemic mixture *rac*‐**17**; bottom left: (*S*)‐**17**; bottom right: (*R*)‐**17**. HPLC conditions: column: Daicel Chiralpak IA (l = 250 mm, d = 4.6 mm); eluent: *iso*‐hexane/*iso*‐propanol = 87:13; flow rate: 1.0 mL/min; detection λ = 254 nm. (*S*)‐**17**: > 99.9% ee; (*R*)‐**17**: > 99.9% ee.

However, analysis of the enantiomeric purity of thioamide *rac*‐**17** and triazole *rac*‐**18** showed two enantiomers, indicating racemization during the reaction of 1,4‐benzodiazepinone **8b** with Lawesson's reagent. Therefore, the enantiomers of thioamide *rac*‐**17** and triazole *rac*‐**18** were separated by semi‐preparative chiral HPLC. The chiral column Daicel Chiralpak AD and the mobile phase iso‐hexane/iso‐propanol 80:20 were used for the separation. HPLC chromatograms of the thioamide **17** before (top) and after separation (bottom) are shown in Figure 4.

**Figure 5:**
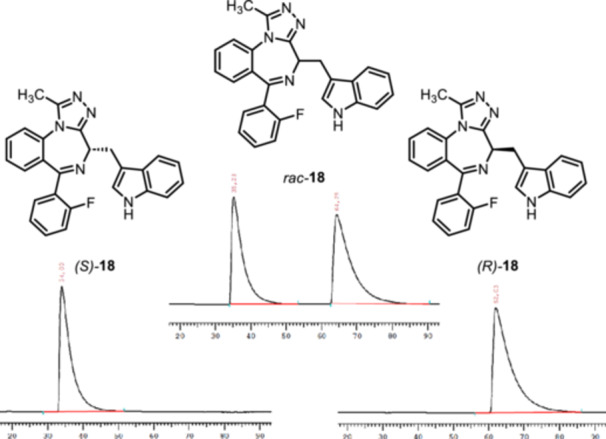
Chiral HPLC chromatograms of triazoles **18**. Top: racemic mixture *rac*‐**18**; bottom left: (*S*)‐**18**; bottom right: (*R*)‐**18**. HPLC conditions: column: Daicel Chiralpak IA (l = 250 mm, d = 4.6 mm); eluent: *iso*‐hexane/*iso*‐propanol = 87:13 + 0.05% Et_2_NH; flow rate: 1.0 mL/min; detection λ = 254 nm. (*S*)‐**18**: 100.0% ee; (*R*)‐**18**: 100.0% ee.

The separation and subsequent analysis of the triazole enantiomers **18** was conducted by the same method (Figure 5).

For the thioamides **17** and the triazoles **18** we hypothesize that the enantiomers with shorter retention time have (*S*)‐configuration and the enantiomers with longer retention time have (*R*)‐configuration. This observation is in accordance with the retention times of 2‐fluorophenyl substituted 1,4‐benzodiazepines **9b** and e*nt‐* **9b**, whose configuration is proved unequivocally by the synthesis from (*R*)‐ or (*S*)‐configured tryptophan. Moreover, (*R*)‐configured enantiomers **9b**, (*R*)‐**17** and (*R*)‐**18** (longer retention time) rotate linearly polarized light to the right, whereas (*S*)‐configured enantiomers *ent*‐**9b**, (*S*)‐**17** and (*S*)‐**18** (shorter retention time) are laevorotatory. This configurational assignment is further supported by the agonistic activity at the K_v_7.1 channel, as the dextrorotatory enantiomers **9b**, (*R*)‐**17** and (*R*)‐**18** show higher agonistic activity than the laevorotatory enantiomers *ent*‐**9b**, (*S*)‐**17** and (*S*)‐**18** (compare Table 1).

**Table 1:** Activity of synthesized 1,4‐benzodiazepines at the K_V_7.1 channel. Data in % refers to the change of channel activity in the presence of test compound (10 µM) compared to the activity in the presence of buffer only. Peak tail currents were measured at −120 mV after a pulse to +40 mV.

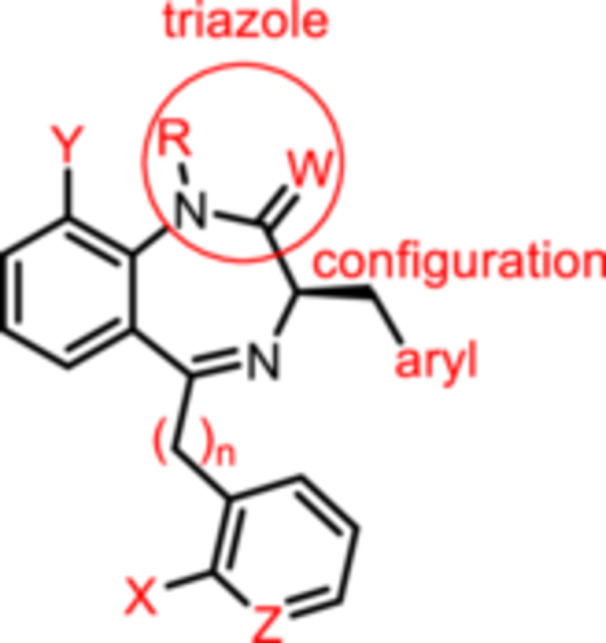									
Compd.	W	X	Y	Z	n	R	Aryl	Configuration	Activity change ± SEM [%]^[a]^
**8a**	O	H	H	CH	0	H	indol‐3‐yl	(*R*)	+6 ± 5
**8b**	O	F	H	CH	0	H	indol‐3‐yl	(*R*)	+85 ± 14
*ent‐* **8b**	O	F	H	CH	0	H	indol‐3‐yl	(*S*)	−13 ± 4
**8c**	O	Cl	H	CH	0	H	indol‐3‐yl	(*R*)	+39 ± 7
**8d**	O	Br	H	CH	0	H	indol‐3‐yl	(*R*)	+11 ± 3
**8e**	O	I	H	CH	0	H	indol‐3‐yl	(*R*)	−3 ± 2
**8f**	O	F	H	CH	1	H	indol‐3‐yl	(*R*)	−29 ± 6
**8g**	O	H	H	N	0	H	indol‐3‐yl	(*R*)	+20 ± 7
**8h**	O	F	OCH_3_	CH	0	H	indol‐3‐yl	(*R*)	−6 ± 2
**8i**	O	F	OH	CH	0	H	indol‐3‐yl	(*R*)	+66 ± 9
**9b** ((*R*)‐L3)	O	F	H	CH	0	CH_3_	indol‐3‐yl	(*R*)	+84 ± 14
*ent‐**9b***	O	F	H	CH	0	CH_3_	indol‐3‐yl	(*S*)	−17 ± 11
**9c**	O	Cl	H	CH	0	CH_3_	indol‐3‐yl	(*R*)	+47 ± 6
**9 d**	O	Br	H	CH	0	CH_3_	indol‐3‐yl	(*R*)	+32 ± 7
**9e**	O	I	H	CH	0	CH_3_	indol‐3‐yl	(*R*)	−4 ± 1
**9 h**	O	F	OCH_3_	CH	0	CH_3_	indol‐3‐yl	(*R*)	+6 ± 4
**9i**	O	F	OH	CH	0	CH_3_	indol‐3‐yl	(*R*)	+92 ± 8
**12a**	O	H	H	CH	0	H	phenyl	(*R*)	−17 ± 2
*ent‐* **12a**	O	H	H	CH	0	H	phenyl	(*S*)	−21 ± 2
**12b**	O	F	H	CH	0	H	phenyl	(*R*)	−16 ± 3
**13a**	O	H	H	CH	0	CH_3_	phenyl	(*R*)	−11 ± 7
*ent‐* **13a**	O	H	H	CH	0	CH_3_	phenyl	(*S*)	−37 ± 4
**14**	O	F	H	CH	0	CH_2_CH_3_	indol‐3‐yl	(*R*)	+24 ± 6
**15**	O	F	H	CH	0	CH_2_CH_2_CH_2_CH_3_	indol‐3‐yl	(*R*)	−21 ± 2
**16**	O	F	H	CH	0	CH_2_CF_3_	indol‐3‐yl	(*R*)	−18 ± 2
*rac*‐**17**	S	F	H	CH	0	H	indol‐3‐yl	*rac*	−33 ± 7
*(R)‐* **17**	S	F	H	CH	0	H	indol‐3‐yl	(*R*)	+13 ± 7
*(S)*‐**17**	S	F	H	CH	0	H	indol‐3‐yl	(*S*)	−24 ± 3
*rac*‐**18**	triazole	F	H	CH	0	triazole	indol‐3‐yl	*rac*	+14 ± 6
*(R)‐* **18**	triazole	F	H	CH	0	triazole	indol‐3‐yl	(*R*)	+24 ± 12
*(S)*‐**18**	triazole	F	H	CH	0	triazole	indol‐3‐yl	(*S*)	−21 ± 3

*Note:* [a] Activity change of the K_V_7.1 channel in the presence of test compound (10 µM) referring to buffer only. Activity was recorded by TEVC. Values represent the mean of at least four independent experiments (*n* ≥ 4).

Abbreviation: TEVC, two‐electrode voltage‐clamp.

A summary correlating the retention times, specific optical rotation and biological activity of enantiomeric pairs is given in Supporting Information: Table S1.

### 2.3 Activity of 1,4‐Benzodiazepines at the K_V_7.1 Channel Measured by TEVC

Oocyte preparation and TEVC experiments were performed as described by Schreiber et al. [28]. In brief, cRNA encoding KCNQ1 was injected into oocytes and afterwards the oocytes were incubated for 2–3 days to overexpress the K_V_7.1 channel. Whole‐cell currents of these oocytes were recorded at room temperature by two‐electrode voltage‐clamp (TEVC). In Table 1, the results of the TEVC experiments are summarized. The effect of each compound on K_V_7.1 channels is given as the change of channel activity (in %) compared to the channel activity of the same oocyte in the presence of only ND96 buffer. The activity was determined from peak tail currents measured at −120 mV after a pulse to +40 mV. The recorded data is summarized in Table 1.

The activity of the secondary lactam **8b** was similar to that of the methylated lead compound **9b** ((R)‐L3). However, with increasing length of the alkyl chain the agonistic activity was consistently reduced in the order methyl (**9b**, +84%) > ethyl (**14**, +24%) > trifluoroethyl (**16**, −18%) > butyl (**15**; −21%). Obviously, the complementary binding pocket of the K_V_7.1 channel does not accept *N*‐substituents larger than a methyl moiety.

(*R*)‐configuration of the center of chirality in 3‐position of the 1,4‐benzodiazpine system appeared to be crucial to activate the K_V_7.1 channel. This is clearly demonstrated by the complete lack of agonistic activity of (*S*)‐configured ligands *ent‐* **8b** (−13%) and *ent‐* **9b** (−17%) compared to their (*R*)‐configured enantiomers **8b** (+85%) and **9b** (+84%). It was further shown that replacement of the indolylmethyl group by a benzyl group completely diminished the agonistic activity of **12b** (−16%) compared to **8b** (+85%). Comparing the activity of the (*R*)‐configured ligand **13a** (−11%) with that of the (*S*)‐configured compound *ent‐* **13a** (−37%) revealed that some (*S*)‐configured 1,4‐benzodiazepins elicit even mild antagonistic effects. This observation is in good accordance with literature reports describing (*R*)‐configured 3‐amino‐1,4‐benzodiazepines with the same orientation of the substituent in 3‐position as (*S*)‐configured 3‐alkyl derivatives such as *ent*‐**13a** as potent K_V_7.1 antagonists [29].

Introduction of a methylene spacer between the 1,4‐benzodiazepine scaffold and the phenyl moiety in 5‐position reversed the agonistic activity of **8b** (+85%) into a weak inhibitory activity of **8f** (−29%). An electron‐withdrawing substituent at 2‐position of the 5‐phenyl moiety seems to be essential to obtain strong agonistic activity. Whereas the 5‐phenyl derivative **8a** led to very weak activation (+6%) of the K_V_7.1 channel, the analogous 2‐fluorphenyl derivative **8b** resulted in +85% activation. Increasing the size and decreasing the electronegativity of the halogen substituent in 2‐postion of the phenyl ring reduced the agonistic activity of the 1,4‐benzodiazepines **8b** (F: +85%) > **8c** (Cl +39%) > **8d** (Br +11%) > **8e** (I: −3%). The same trend applies to the 1‐methylated lactams **9b**–**e**.

Bioisosteric replacement of the amide with a thioamide in *(R)‐* **17** (+13%) and triazole in *(R)*‐**18** (+24%) decreased the agonistic activity of the lactam **8b** (+85%). The reduced agonistic activity of thioamide *(R)‐* **17** and triazole *(R)‐* **18** compared to amide **8b** further supports the concept that the binding pocket does not tolerate large substituents in 1‐ and/or 2‐position.

The 9‐methoxy substituted 1,4‐benzodiazepins **8h** (−6%) and **9h** (+6%) did not activate the K_V_7.1 channel. However, the corresponding 9‐hydroxy derivative **8i** (+66%) has only slightly lower activity than the lead compound **9b** (+84%) and the 1‐methyl analog **9i** (+92%) appeared to be even more potent at K_V_7.1. channels. Due to the high variability of the results obtained by TEVC recordings, the activities of phenols **8i** and **9i** and the lead compound **9b** do not differ significantly.

The three most potent compounds (**8b**, **9b**, and **9i**) of the first screening at 10 µM were selected to investigate their dose‐response dependency together with (*R*)‐**18**, which has a much lower activity (+24%) than the other ones, but the highest metabolic stability of all tested compounds (see Table 3 below). For this purpose, (*R*)‐configured secondary amide **8b**, phenol **9i** and triazole *(R)‐* **18** as well as the lead compound **9b** ((R)‐L3) were tested additionally at concentrations of 100 nM and 1 µM.

**Figure 6:**
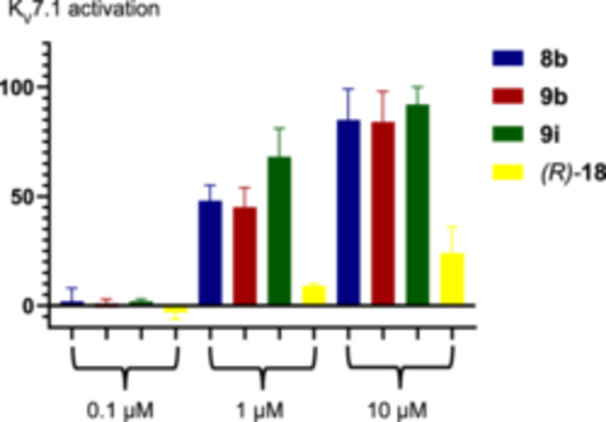
Activation of the K_V_7.1 channel by different concentrations of the 1,4‐benzodiazepines **8b**, **9b**, **9i**, and (*R*)*‐* **18**.

The results are summarized in Table 2 and Figure 6. At a concentration of 100 nM, the test compounds were not able to activate the K_V_7.1 channel, they could not increase the ion current across the oocyte membrane. However, at a concentration of 1 µM, the potency of the three new test compounds was clearly different, Whereas the secondary lactam **8b** (+48%) exhibited the same K_V_7.1 activation as the tertiary lactam **9b** (+45%), the triazole (*R*)‐**18** (+9%) was less potent and the phenol **9i** (+68%) was more potent than the lead compound **9b**.

**Table 2:** Activity increase at K_V_7.1 channels using different concentrations of selected 1,4‐benzodiazepines **8b, 9b, 9i**, and (*R*)*‐* **18**. Data in % refers to the change of channel activity in the presence of test compound compared to the activity in the presence of buffer only. Peak tail currents were measured at −120 mV after a pulse to +40 mV.

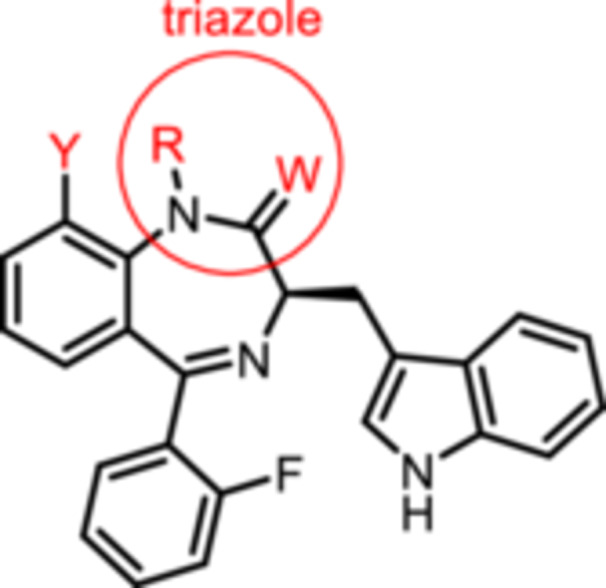						
				Activity change ± SEM [%] at different concentrations^[a]^		
Compd.	W	Y	R	100 nM	1 µM	10 µM
**8b**	O	H	H	+2 ± 6	+48 ± 7	+85 ± 14
**9b**	O	H	CH_3_	+1 ± 2	+45 ± 9	+84 ± 14
**9i**	O	OH	CH_3_	+2 ± 1	+68 ± 13	+92 ± 8
(*R*)‐**18**	triazole	H	triazole	−3 ± 3	+9 ± 1	+24 ± 12

*Note:* [a] Activity change of the KV7.1 channel in the presence of different concentrations of test compounds referring to buffer only. Activity was recorded by TEVC. Values represent the mean of at least four independent experiments (*n* ≥ 4).

Abbreviations: SEM, standard error of mean; TEVC, two‐electrode voltage‐clamp.

### 2.4 Physicochemical and Pharmacokinetic Properties of Selected Ligands

Some pharmacokinetic parameters were recorded for the most promising (*R*)‐configured test compounds **8b**, **9b**, **9i**, and (*R*)*‐* **18**. The logD_7.4_ values were determined by the micro‐shake flask method using MS quantification of the test compounds in the MOPS buffer layer [30]. For the analysis of plasma protein binding (PPB), a stationary phase coated with human serum albumin was used in high‐performance affinity chromatography (HPAC) [31]. After incubation with mouse liver microsomes and NADPH at 37°C for 90 min, the amount of parent was determined by LC‐MS. The amount of intact K_v_7.1 activator is a measure for its stability during phase I metabolism (Table 3) [32].

**Table 3:** logD_7.4_ values and some pharmacokinetic parameters of 1,4‐benzodiazeines **8b, 9b, 9i**, and (*R*)*‐* **18**.

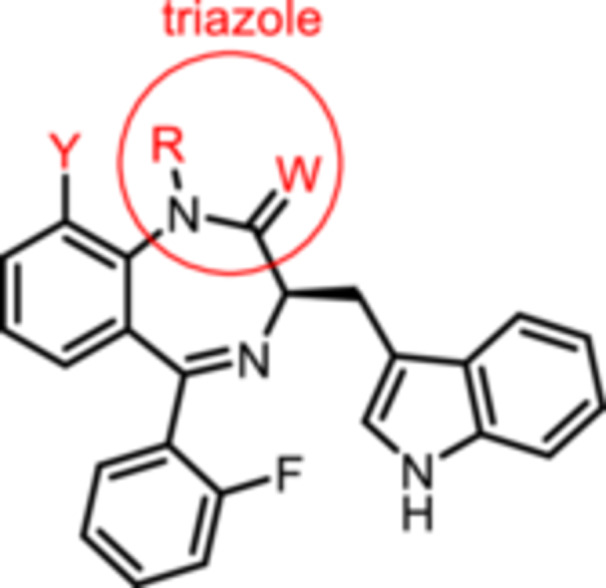						
compd.	´W	Y	R	logD_7.4_ ± SD (*n* = 3)	Plasma protein binding (%)^a^	Metabolic stability ± SD (%) (*n* = 3)^b^
**8b**	O	H	H	3.99 ± 0.07	> 98	59 ± 1.3
**9b**	O	H	CH_3_	4.08 ± 0.12	> 98	62 ± 5.9
**9i**	O	OH	CH_3_	4.16 ± 0.02	> 98	73 ± 7.9
(*R*)*‐* **18**	triazole	H	triazole	3.31 ± 0.06	> 98	82 ± 4.5
Imipramine^c^					> 98	26 ± 2.1

Abbreviations: HPAC, high‐performance affinity chromatography; SD, standard deviation.

^a^ Plasma protein binding was determined by HPAC.

^b^ Amount (in %) of parent compound after incubation with mouse liver microsomes and NADPH for 90 min.

^c^ Imipramine was used as a reference compound to prove the activity of the assay.

The tested compounds had very similar logD_7.4_ values in the range of 4. Since their logD_7.4_ values are below 5, they obey Lipinski's rule of five [33]. As expected, methylation of the secondary lactam **8b** increased the logD_7.4_ value of the tertiary lactam **9b** slightly. However, an unexpected increase of the logD_7.4_ value to 4.16 was observed for the phenol **9i** containing an additional OH moiety. The triazole represents the most polar ligand of this series with a logD_7.4_ value of 3.31 (Table 3).

All compounds show very high plasma protein binding of > 98%, which is characteristic for drugs with a 1,4‐benzodiazepin‐2‐one scaffold [34].

The amount of intact parent compound after an incubation period of 90 min with mouse liver microsomes and NADPH ranged between 59% (**8b**) and 82% ((*R*)*‐* **18**). Interestingly, phenol **9i** with the highest K_v_7.1 activation showed higher phase I metabolic stability than **9b**: after 90 min, 73% of **9i** remained unchanged, whereas only 62% of intact lead compound **9b** were detected after 90 min. The highest metabolic stability (82% intact after 90 min) was observed for the triazole (*R*)‐**18**.

## 3 Conclusion

The 3‐[(indol‐3‐yl)methyl]−1,4‐benzodiazepine (R)‐L3 (**9b**) was described as a potent activator of the K^+^ channel K_V_7.1 [17], but detailed relationships between structural modifications and K_V_7.1 activation were not reported. In this study, various 3‐arylmethyl substituted 1,4‐benzodiazepines were designed and 31 derivatives were prepared and pharmacologically evaluated. The influence of the synthesized 1,4‐benzodiazepines on the K_V_7.1 channel activity was recorded by TEVC experiments providing comprehensive insights into the structural requirements for K_V_7.1 modulation.

(*R*)‐Configuration at C‐3 proved to be crucial for K_V_7.1 activation: Whereas (*R*)‐configured 1,4‐benzodiazepines **8b** and **9b** considerably increased the ion current along the ion channel, the (*S*)‐configured enantiomers *ent‐* **8b** and *ent‐* **9b** showed complete loss of agonistic activity.

In addition to the C‐3 configuration, the 1,4‐benzodiazepine core of **9b** was modified at seven positions. Replacement of the F‐atom in 2‐position of the 5‐phenyl moiety by a proton or other halogen‐atoms resulted in reduced activity. A proton or a small CH_3_ moiety at N‐1 are well tolerated by the K_V_7.1 channel, but longer alkyl side chains, a thioamide or embedding the amide into a triazole considerably reduced the agonistic activity. The indol‐3‐yl moiety appeared to be essential for K_V_7.1 activation, as its replacement by a phenyl moiety led only to weak inhibition of the ion flux.

Unexpectedly, the phenol **9i** with an OH moiety at 9‐position showed higher agonistic activity at the K_V_7.1 channel than the lead compound **9b**. Although its lipophilicity was slightly increased (logD_7.4_ = 4.16), its metabolic stability was also increased revealing 73% intact parent compound after incubation for 90 min with mouse liver microsomes and NADPH.

## 4 Experimental Section

### 4.1 Chemistry

#### 4.1.1 Chemistry, General Methods

NMR spectroscopy: NMR spectra were recorded in deuterated solvents on Agilent DD2 400 MHz and 600 MHz spectrometers (Agilent, Santa Clara CA, USA); chemical shifts (*δ*) are reported in parts per million (ppm) against the reference substance tetramethylsilane and calculated using the solvent residual peak of the non‐deuterated solvent; coupling constants are given with 0.5 Hz resolution; assignment of ^1^H and ^13^C NMR signals was supported by 2‐D NMR techniques where necessary. Optical rotation: Polarimeter 341 (Perkin Elmer); 1.0 dm tube; concentration *c* in g/100 mL; T = 20°C; wavelength 589 nm (D‐line of Na light); the unit of the specific rotation ([α]_D_^T^ grad.mL.dm^−1^.g^−1^) is omitted for clarity.

#### 4.1.2 HPLC Method 1 for the Determination of the Purity

Equipment 1: Pump: L‐7100, degasser: L‐7614, autosampler: L‐7200, UV detector: L‐7400, interface: D‐7000, data transfer: D‐line, data acquisition: HSM‐Software (all from Merck Hitachi, Darmstadt, Germany); Equipment 2: Pump: LPG‐3400SD, degasser: DG‐1210, autosampler: ACC‐3000T, UV‐detector: VWD‐3400RS, interface: DIONEX UltiMate 3000, data acquisition: Chromeleon 7 (equipment and software from Thermo Fisher Scientific, Lauenstadt, Germany); column: LiChrospher® 60 RP‐select B (5 µm), LiChroCART® 250‐4 mm cartridge; flow rate: 1.0 mL/min; injection volume: 5.0 µL; detection at λ = 210 nm; solvents: A: demineralized water with 0.05% (V/V) trifluoroacetic acid, B: CH_3_CN with 0.05% (V/V) trifluoroacetic acid; gradient elution (% A): 0–4 min: 90%; 4–29 min: gradient from 90% to 0%; 29–31 min: 0%; 31–31.5 min: gradient from 0% to 90%; 31.5–40 min: 90%. Unless otherwise mentioned, the purity of all test compounds is greater than 95%.

#### 4.1.3 Synthetic Procedures


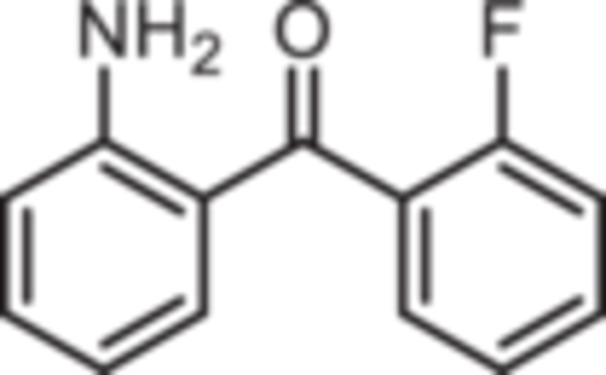


(2‐Aminophenyl)‐(2‐fluorophenyl)methanone (**4b**): At 0°C a solution of BCl_3_ in p‐xylene (1 M, 11 mL, 11 mmol, 1.1 eq.) was added to a solution of aniline (0.92 mL, 931 mg, 10 mmol, 1.0 eq.) in xylene (2 mL). Then, 2‐fluorobenzonitrile (2.18 mL, 2.42 g, 20 mmol, 2.0 eq.), AlCl_3_ (1.47 g, 11 mmol, 1.1 eq.) and additional xylene (2 mL) were added. The ice bath was removed and the mixture was stirred in a preheated (144°C) oil bath for 16 h. After cooling to room temperature, water (22 mL) was added and the mixture was stirred at 100°C for 40 min. Ethyl acetate (35 mL) and water (28 mL) were added and the aqueous layer was extracted with ethyl acetate (3 × 50 mL). The organic layer was dried (Na_2_SO_4_), filtered and concentrated in vacuo. The residue was purified by flash chromatography (SiO_2_, cyclohexane/ethyl acetate, gradient 100:0 → 0:100). Yellow solid, mp 127°C, yield 1.64 g (7.6 mmol, 76%). Purity (HPLC): 97.9% (t_R_ = 20,6 min). Chemical formula: C_13_H_10_FNO (215.2 g/mol). TLC: R_f_ = 0.59 (cyclohexane/ethyl acetate 70:30). ^1^H NMR (600 MHz, DMSO‐d_6_): δ (ppm) = 6.47 (ddd, *J* = 8.1/6.9/1.1 Hz, 1H, 5‐*H*_PhNH__2_), 6.86 (dd, *J* = 8.5/1.1 Hz, 1H, 3‐*H*_PhNH__2_), 7.10 (m, 1H, 6‐*H*_PhNH__2_), 7.29 (ddd, *J* = 8.5/6.9/1.6 Hz, 1H, 4‐*H*_PhNH__2_), 7.33 (m, 2H, 6‐*H*_FPhe_, 3‐*H*_FPhe_), 7.43 (m, 3H, N*H*_2_, 5‐*H*_FPhe_), 7.57 (m, 1H, 4‐*H*_FPhe_). ^13^C NMR (151 MHz, DMSO‐d_6_): δ (ppm) = 114.5 (C‐5_PhNH__2_), 115.8 (d, *J* = 21.6 Hz, C‐3_FPhe_), 116.3 (C‐1_PhNH__2_), 116.9 (C‐3_PhNH__2_), 124.6 (d, *J* = 3.3 Hz, C‐6_FPhe_), 128.5 (d, *J* = 17.3 Hz, C‐1_FPhe_), 129.3 (d, *J* = 3.7 Hz, C‐5_FPhe_), 131.9 (d, *J* = 8.1 Hz, C‐4_FPhe_), 133.8 (C‐6_PhNH__2_), 135.1 (C‐4_PhNH__2_), 152.1 (C‐2_PhNH__2_), 158.1 (d, *J* = 245.8 Hz, C‐2_FPhe_), 194.1 (C=O). IR (neat): ν̃ (cm^−1^) = 3433 and 3317 (NH_2_), 1620 (C=O). Exact mass (APCI): *m/z* = 216.0827 (calcd 216.0819 for C_13_H_11_FNO [M+H]^+^).


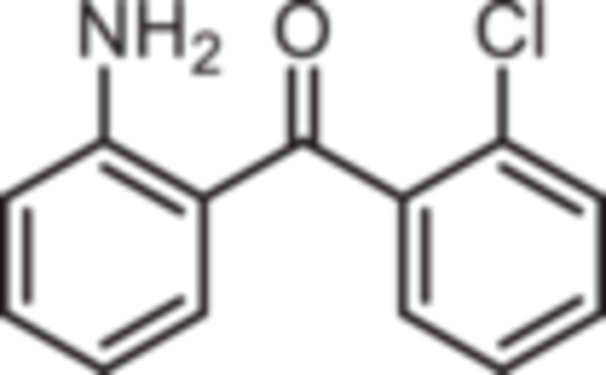


(2‐Aminophenyl)‐(2‐chlorophenyl)methanone (**4c**): At 0°C a solution of BCl_3_ in p‐xylene (1 M, 11 mL, 11 mmol, 1.1 eq.) was added to a solution of aniline (0.92 mL, 931 mg, 10 mmol, 1.0 eq.) in xylene (2 mL). Then, 2‐chlorobenzonitrile (2.75 g, 20 mmol, 2.0 eq.), AlCl_3_ (1.47 g, 11 mmol, 1.1 eq.) and additional xylene (2 mL) were added. The ice bath was removed and the mixture was stirred in a preheated (144°C) oil bath for 16 h. After cooling to room temperature, water (22 mL) was added and the mixture was stirred at 100°C for 40 min. Ethyl acetate (35 mL) and water (28 mL) were added and the aqueous layer was extracted with ethyl acetate (3 × 50 mL). The organic layer was dried (Na_2_SO_4_), filtered and concentrated in vacuo. The residue was purified by flash chromatography (SiO_2_, cyclohexane/ethyl acetate, gradient 100:0 → 0:100). Slightly yellow solid, mp 69°C–70°C, yield 1.46 g (6.3 mmol, 63%). Purity (HPLC): 99.3% (t_R_ = 20.7 min). Chemical formula: C_13_H_11_ClNO (231.7 g/mol). TLC: R_f_ = 0.32 (cyclohexane/ethyl acetate 90:10). ^1^H NMR (600 MHz, DMSO‐d_6_): δ (ppm) = 6.44 (ddd, *J* = 8.1/6.9/1.1 Hz, 1H, 5‐*H*_PhNH__2_), 6.86 (dt, *J* = 8.5/1.3 Hz, 1H, 3‐*H*_PhNH__2_), 6.94 (dd, *J* = 8.1/1.6 Hz, 1H, 6‐*H*_PhNH__2_), 7.28 (ddd, *J* = 8.4/6.8/1.6 Hz, 1H, 4‐*H*_PhNH__2_), 7.39 (dd, *J* = 7.5/1.7 Hz, 1H, 3‐*H*_ClPhe_), 7.43–7.54 (m, 4H, 4‐*H*_ClPhe_, 5‐*H*_ClPhe_, N*H*_2_), 7.56 (dd, *J* = 8.1/1.2 Hz, 1H, 6‐*H*_ClPhe_). ^13^C NMR (151 MHz, DMSO‐d_6_): δ (ppm) = 114.5 (C‐5_PhNH__2_), 115.7 (C‐1_PhNH__2_), 116.9 (C‐3_PhNH__2_), 127.2 (C‐4_ClPh_), 128.3 (C‐3_ClPh_), 129.2 (C‐1_ClPh_), 129.5 (C‐6_ClPh_), 130.6 (C‐5_ClPh_), 133.8 (C‐6_PhNH__2_), 135.1 (C‐4_PhNH__2_), 139.7 (C‐2_ClPh_), 152.3 (C‐2_PhNH__2_), 195.9 (C=O). IR (neat): ν̃ (cm^−1^) = 3429 and 3309 (NH_2_), 1649 (C=O). Exact mass (APCI): *m/z* = 232.0512 (calcd 232.0524 for C_13_H_11_ClNO [M+H]^+^).


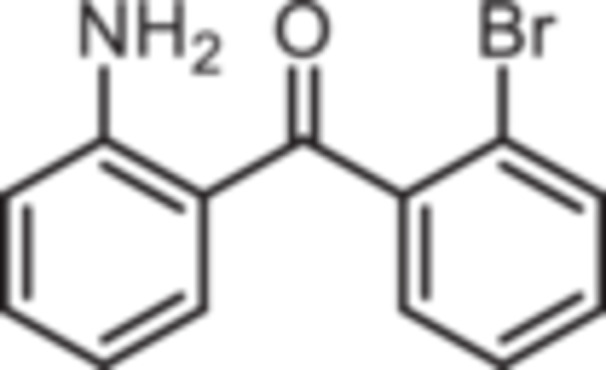


(2‐Aminophenyl)‐(2‐bromophenyl)methanone (**4d**): At 0°C a solution of BCl_3_ in p‐xylene (1 M, 11 mL, 11 mmol, 1.1 eq.) was added to a solution of aniline (0.92 mL, 931 mg, 10 mmol, 1.0 eq.) in xylene (2 mL). Then, 2‐bromobenzonitrile (3.64 g, 20 mmol, 2.0 eq.), AlCl_3_ (1.47 g, 11 mmol, 1.1 eq.) and additional xylene (2 mL) were added. The ice bath was removed and the mixture was stirred in a preheated (144°C) oil bath for 16 h. After cooling to room temperature, water (22 mL) was added and the mixture was stirred at 100°C for 40 min. Ethyl acetate (35 mL) and water (28 mL) were added and the aqueous layer was extracted with ethyl acetate (3 × 50 mL). The organic layer was dried (Na_2_SO_4_), filtered and concentrated in vacuo. The residue was purified by flash chromatography (SiO_2_, cyclohexane/ethyl acetate, gradient 100:0 → 0:100). Slightly yellow solid, mp 69°C–70°C, yield 2.26 g (8.2 mmol, 82%). Purity (HPLC): 88.4% (t_R_ = 21.5 min). Chemical formula: C_13_H_11_BrNO (276.1 g/mol). TLC: R_f_ = 0.28 (cyclohexane/ethyl acetate 90:10). ^1^H NMR (600 MHz, DMSO‐d_6_): δ (ppm) = 6.43 (ddd, *J* = 8.1/6.9/1.1 Hz, 1H, 5‐*H*_PhNH__2_), 6.85 (dd, *J* = 8.5/1.1 Hz, 1H, 3‐*H*_PhNH__2_), 6.92 (dd, *J* = 8.2/1.5 Hz, 1H, 6‐*H*_PhNH__2_), 7.28 (ddd, *J* = 8.5/6.9/1.6 Hz, 1H, 4‐*H*_PhNH__2_), 7.37 (dd, *J* = 7.5/1.7 Hz, 1H, 3‐*H*_BrPhe_), 7.42 (td, *J* = 7.7/1.7 Hz, 1H, 4‐*H*_BrPhe_), 7.46–7.53 (m, 3H, 5‐*H*_BrPhe_, N*H*_2_), 7.71 (dd, *J* = 8.0/1.1 Hz, 1H, 6‐*H*_BrPhe_). ^13^C NMR (151 MHz, DMSO‐d_6_): δ (ppm) = 114.4 (C‐5_PhNH__2_), 115.4 (C‐1_PhNH__2_), 117.0 (C‐3_PhNH__2_), 118.3 (C‐1_BrPhe_), 127.7 (C‐5_BrPhe_), 128.2 (C‐3_BrPhe_), 130.7 (C‐4_BrPhe_), 132.6 (C‐6_BrPhe_), 133.9 (C‐6_PhNH__2_) 135.1 (C‐4_PhNH__2_), 141.7 (C‐2_BrPhe_), 152.4 (C‐2_PhNH__2_), 196.7 (C=O). IR (neat): ν̃ (cm^−1^) = 3441 and 3325 (NH_2_), 1612 (C=O). Exact mass (APCI): *m/z* = 276.0008 (calcd 276.0019 for C_13_H_11_BrNO [M+H]^+^).


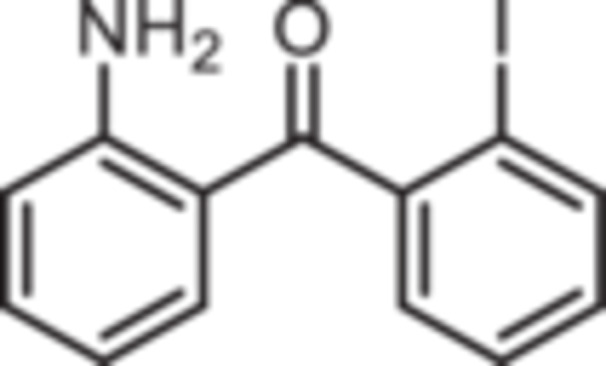


(2‐Aminophenyl)‐(2‐iodophenyl)methanone (**4e**): At 0°C a solution of BCl_3_ in p‐xylene (1 M, 11 mL, 11 mmol, 1.1 eq.) was added to a solution of aniline (0.92 mL, 931 mg, 10 mmol, 1.0 eq.) in xylene (2 mL). Then, 2‐iodobenzonitrile (4.58 g 20 mmol, 2.0 eq.), AlCl_3_ (1.47 g, 11 mmol, 1.1 eq.) and additional xylene (2 mL) were added. The ice bath was removed and the mixture was stirred in a preheated (144°C) oil bath for 16 h. After cooling to room temperature, water (22 mL) was added and the mixture was stirred at 100°C for 40 min. Ethyl acetate (35 mL) and water (28 mL) were added and the aqueous layer was extracted with ethyl acetate (3 × 50 mL). The organic layer was dried (Na_2_SO_4_), filtered and concentrated in vacuo. The residue was purified by flash chromatography (SiO_2_, cyclohexane/ethyl acetate, gradient 100:0 → 0:100). Slightly yellow solid, mp 113°C, yield 2.94 g (9.1 mmol, 91%). Purity (HPLC): 99.2% (t_R_ = 21.2 min). Chemical formula: C_13_H_11_INO (323.1 g/mol). TLC: R_f_ = 0.30 (cyclohexane/ethyl acetate 90:10). ^1^H NMR (600 MHz, DMSO‐d_6_): δ (ppm) = 6.43 (ddd, *J* = 8.1/6.9/1.1 Hz, 1H, 5‐*H*_PhNH__2_), 6.85 (dd, *J* = 8.5/1.1 Hz, 1H, 3‐*H*_PhNH__2_), 6.90 (dd, *J* = 8.2/1.6 Hz, 1H, 6‐*H*_PhNH__2_), 7.23 (td, *J* = 7.7/1.7 Hz, 1H, 4‐*H*_PhNH__2_), 7.28 (m, 2 H, 4‐*H*_IPhe_, 5‐*H*_IPhe_), 7.45–7.50 (m, 3H, N*H*_2,_ 6‐*H*_IPhe_), 7.93 (dd, *J* = 8.0/1.1 Hz, 1H, 3‐*H*_IPhe_). ^13^C NMR (151 MHz, DMSO‐d_6_): δ (ppm) = 92.9 (C‐2_IPhe_), 114.4 (C‐5_PhNH__2_), 114.9 (C‐1_PhNH__2_), 116.9 (C‐3_PhNH__2_), 127.4 (C‐5_IPhe_), 128.0 (C‐6_IPhe_), 130.5 (C‐4_PhNH__2_), 134.0 (C‐6_PhNH__2_), 135.0 (C‐4_IPhe_), 138.8 (C‐3_IPhe_), 145.7 (C‐1_IPhe_), 152.5 (C‐2_PhNH__2_), 198.6 (C=O). IR (neat): ν̃ (cm^−1^) = 3441 and 3325 (NH_2_), 1622 (C=O). Exact mass (APCI): *m/z* = 323.9879 (calcd 323.9880 for C_13_H_11_INO [M+H]^+^).


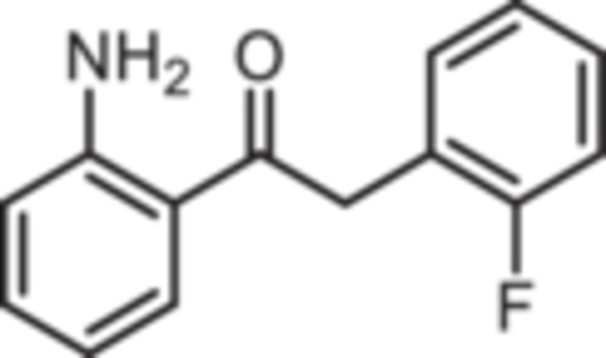


1‐(2‐Aminophenyl)−2‐(2‐fluorophenyl)ethan−1‐one (**4f**): At 0°C a solution of BCl_3_ in p‐xylene (1 M, 11 mL, 11 mmol, 1.1 eq.) was added to a solution of aniline (0.92 mL, 931 mg, 10 mmol, 1.0 eq.) in xylene (2 mL). Then, (2‐fluorphenyl)acetonitrile (2.40 mL, 2.70 g 20 mmol, 2.0 eq.), AlCl_3_ (1.47 g, 11 mmol, 1.1 eq.) and additional xylene (2 mL) were added. The ice bath was removed and the mixture was stirred in a preheated (144°C) oil bath for 16 h. After cooling to room temperature, water (22 mL) was added and the mixture was stirred at 100°C for 40 min. Ethyl acetate (35 mL) and water (28 mL) were added and the aqueous layer was extracted with ethyl acetate (3 × 50 mL). The organic layer was dried (Na_2_SO_4_), filtered and concentrated in vacuo. The residue was purified by flash chromatography (SiO_2_, cyclohexane/ethyl acetate, gradient 100:0 → 0:100). Slightly yellow solid, mp 90°C–91°C, yield 1.78 g (7.8 mmol, 78%). Purity (HPLC): 97.7% (t_R_ = 20.6 min). Chemical formula: C_14_H_12_FNO (229.3 g/mol). TLC: R_f_ = 0.28 (cyclohexane/ethyl acetate 90:10). ^1^H NMR (600 MHz, DMSO‐d_6_): δ (ppm) = 4.37 (s, 2H, C*H*_2_), 6.58 (ddd, *J* = 8.1/6.9/1.2 Hz, 1H, 5‐*H*_PhNH__2_), 6.78 (dd, *J* = 8.4/1.2 Hz, 1H, 3‐*H*_PhNH__2_), 7.12–7.20 (m, 4H, N*H*_2_, 3‐*H*_FPhe_, 5‐*H*_FPhe_), 7.23–7.34 (m, 3H, 4‐*H*_FPhe_, 6‐*H*_FPhe_, 4‐*H*_PhNH__2_), 7.92 (dd, *J* = 8.2/1.5 Hz, 1H, 6‐*H*_PhNH__2_). ^13^C NMR (151 MHz, DMSO‐d_6_): δ (ppm) = 39.1 (d, *J* = 1.6 Hz, *C*H_2_), 114.5 (C‐5_PhNH__2_), 114.9 (d, *J* = 21.5 Hz, C‐3_FPhe_), 116.0 (C‐1_PhNH__2_), 117.0 (C‐3_PhNH__2_), 123.3 (d, *J* = 16.5 Hz, C‐1_FPhe_), 124.1 (d, *J* = 3.4 Hz C‐5_FPhe_), 128.6 (d, *J* = 8.1 Hz, C‐4_FPhe_), 131.4 (C‐6_PhNH__2_), 132.3 (d, *J* = 4.7 Hz, C‐6_FPhe_), 134.3 (C‐4_PhNH__2_), 151.3 (C‐2_PhNH__2_), 160.8 (d, *J* = 243.5 Hz, C‐2_FPhe_), 197.8 (C=O). IR (neat): ν̃ (cm^−1^) = 3441 and 3325 (NH_2_), 1622 (C=O). Exact mass (APCI): *m/z* = 323.9879 (calcd 323.9880 for C_13_H_11_INO [M+H]^+^).


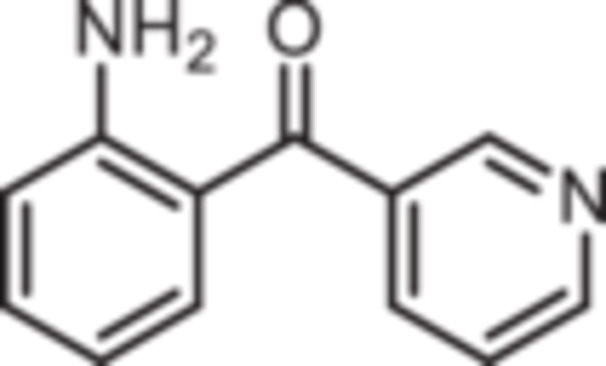


(2‐Aminophenyl)‐(pyridin‐3‐yl)methanone (**4g**): At 0°C a solution of BCl_3_ in p‐xylene (1 M, 26 mL, 26 mmol, 2.6 eq.) was added to a solution of aniline (1.19 mL, 1.21 g, 13 mmol, 1.3 eq.) in xylene (14 mL). Then, pyridine‐3‐carbonitrile (0.90 mL, 1.04 g, 10 mmol, 1.0 eq.), AlCl_3_ (3.47 g, 26 mmol, 2.6 eq.) and additional xylene (15 mL) were added. The ice bath was removed and the mixture was stirred in a preheated (144°C) oil bath for 6 h. After cooling down to room temperature, 3 M HCl (25 mL) was added and the mixture was stirred at 100°C for 1 h. After cooling down to room temperature 3 M NaOH (400 mL) and CH_2_Cl_2_ (400 mL) were added. The mixture was filtered and the aqueous layer was extracted with CH_2_Cl_2_ (3 × 400 mL). The organic layer was dried (Na_2_SO_4_), filtered and concentrated in vacuo. The residue was purified by flash chromatography (SiO_2_, cyclohexane/ethyl acetate, gradient 100:0 → 0:100). Yellow solid, mp 83°C–84°C, yield 0.26 g (1.3 mmol, 13%). Purity (HPLC): 98.5% (t_R_ = 11.6 min). Chemical formula: C_12_H_10_N_2_O (198.2 g/mol). TLC: R_f_ = 0.27 (cyclohexane/ethyl acetate 70:30). ^1^H NMR (600 MHz, DMSO‐d_6_): δ (ppm) = 6.52 (ddd, *J* = 8.1/6.9/1.2 Hz, 1H, 5‐*H*_PhNH__2_), 6.87 (dd, *J* = 8.4/1.2 Hz, 1H, 3‐*H*_PhNH__2_), 7.23–7.28 (m, 3H, 6‐*H*_PhNH__2_, NH_2_), 7.31 (ddd, *J* = 8.5/6.9/1.6 Hz, 1H, 4‐*H*_PhNH__2_), 7.54 (ddd, *J* = 7.8/4.9/0.9 Hz, 1H, 5‐*H*_pyridine_), 7.94 (m, 1H, 4‐H_pyridine_), 8.71 (dd, *J* = 2.2/0.9 Hz, 1H, 2‐*H*_pyridine_), 8.74 (dd, *J* = 4.9/1.7 Hz, 1H, 6‐*H*_pyridine_). ^13^C NMR (151 MHz, DMSO‐d_6_): δ (ppm) = 114.4 (C‐5_PhNH2_), 116.0 (C‐1_PhNH2_), 117.0 (C‐3_PhNH2_), 123.4 (C‐5_pyridine_), 133.8 (C‐6_PhNH2_), 134.8 (C‐4_PhNH2_), 135.6 (C‐3_pyridine_), 136.1 (C‐4_pyridine_), 148.8 (C‐2_pyridine_), 151.4 (C‐6_pyridine_), 152.2 (C‐2_PhNH2_), 195.9 (C=O). IR (neat): ν̃ (cm^−1^) = 3360 and 3186 (NH_2_), 1667 (C=O). Exact mass (APCI): *m/z* = 199.0871 (calcd 199.0866 for C_12_H_11_N_2_O [M+H]^+^).


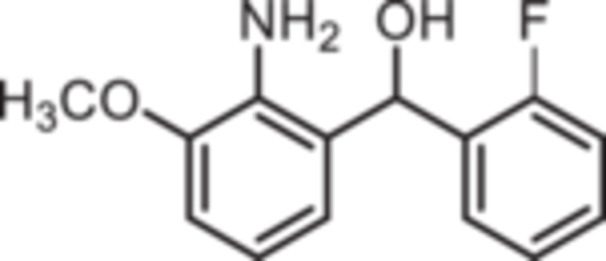


(2‐Amino‐3‐methoxyphenyl)‐(2‐fluorophenyl)methanol (**5**): At −20°C 2‐methoxyaniline (1.65 mL, 1.72 g, 14 mmol, 1.0 eq.) was added to a solution of phenyldichloroboran (1.80 mL, 2.22 g, 14 mmol, 1.0 eq.) in CH_2_Cl_2_ (42 mL). Et_3_N (4.88 mL, 3.54 g, 35 mmol, 2.5 eq.) was added. Stirring was continued at −20°C for 30 min. A solution of 2‐fluorbenzaldehyde (0.65 mL, 1.74 g, 14 mmol) in CH_2_Cl_2_ (56 mL) was added. The solution was stirred for 5 h at room temperature. Ice water (100 mL) was added and the aqueous layer was extracted with CH_2_Cl_2_ (3 × 100 mL). The combined organic layer was washed with brine (100 mL), dried (Na_2_SO_4_), filtered and concentrated in vacuo. The residue was dissolved in THF (9 mL). 2 M NaOH (28 mL) was added and the solution was stirred at room temperature for 1 h. The aqueous layer was extracted with CH_2_Cl_2_ (3 × 50 mL). Then the organic layer was dried (Na_2_SO_4_), filtered and concentrated in vacuo. The residue was purified by flash chromatography (SiO_2_, cyclohexane/ethyl acetate, gradient 100:0 → 0:100). Colorless solid, mp 142°C–143°C, yield 1.83 g (7.4 mmol, 53%). Purity (HPLC): 99.2% (t_R_ = 14.2 min). Chemical formula: C_14_H_14_FNO_2_ (247.3 g/mol). TLC: R_f_ = 0.25 (cyclohexane/ethyl acetate 80:20). ^1^H NMR (600 MHz, DMSO‐d6): δ (ppm) = 3.76 (s, 3H, OC*H*_3_), 4.63 (s, 2H, N*H*_2_), 5.94 (dd, *J* = 5.2/1.6 Hz, 1H, O*H*), 5.98 (dd, 1H, *J* = 5.2/1.6 Hz, C*H*), 6.47–6.53 (m, 2H, 4‐*H*_PhNH2_, 6‐*H*_PhNH2_), 6.75 (dd, *J* = 6.4/3.0 Hz, 1H, 5‐*H*_PhNH2_), 7.11 (m, 1H, 3‐*H*_FPhe_), 7.20 (td, *J* = 7.5/1.2 Hz, 1H, 5‐*H*_FPhe_), 7.31 (m, 1H, 4‐*H*_FPhe_), 7.56 (m, 1H, 6‐*H*_FPhe_). ^13^C NMR (151 MHz, DMSO‐d6): δ (ppm) = 55.5 (O*C*H_3_), 65.9 (d, *J* = 2.8 Hz, *C*OH), 109.5 (C‐5_PhNH2_), 114.9 (d, *J* = 21.5 Hz, C‐3_FPhe_), 115.6 (C‐4_PhNH2_), 119.6 (C‐6_PhNH2_), 124.0 (d, *J* = 3.4 Hz, C‐5_FPhe_), 126.7 (C‐1_PhNH2_), 128.5 (d, *J* = 4.4 Hz, C‐6_FPhe_), 128.8 (d, *J* = 8.1 Hz, C‐4_FPhe_), 130.8 (d, *J* = 13.5 Hz, C‐1_FPhe_), 135.1 (C‐2_PhNH2_), 146.7 (C‐3_PhNH__2_), 159.3 (d, *J* = 244.5 Hz, C‐2_FPhe_). IR (neat): ν̃ (cm^−1^) = 3406 and 3325 (OH, NH_2_), 1612 (C=C). Exact mass (APCI): *m/z* = 248.1087 (calcd 248.1081 for C_14_H_15_FNO_2_ [M+H]^+^).


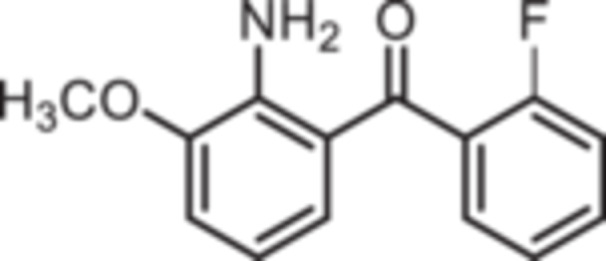


(2‐Amino‐3‐methoxyphenyl)‐(2‐fluorophenyl)methanone (**4h**): A mixture of **5** (2.47 g, 10 mmol, 1.0 eq.) and MnO_2_ (4.00 g, 46 mmol, 46.0 eq.) in CH_2_Cl_2_ (75 mL) and THF (15 mL) was stirred at 40°C. After 16 h, the mixture was filtered and concentrated in vacuo. The residue was purified by flash chromatography (SiO_2_, cyclohexane/ethyl acetate, gradient 100:0 → 0:100). Yellow solid, mp 56°C–57°C, yield 2.28 g (9.3 mmol, 93%). Purity (HPLC): 100.0% (t_R_ = 20.9 min). Chemical formula: C_14_H_12_FNO_2_ (245.3 g/mol). TLC: R_f_ = 0.20 (cyclohexane/ethyl acetate 95:5). ^1^H NMR (600 MHz, DMSO‐d_6_): δ (ppm) = 3.86 (s, 3H, OC*H*_3_), 6.47 (dd, *J* = 8.3/7.7 Hz, 1H, 5‐*H*_PhNH2_), 6.74 (dd, *J* = 8.4/1.2 Hz, 1H, 6‐*H*_PhNH2_), 7.02 (dd, *J* = 7.8/1.2 Hz, 1H, 4‐*H*_PhNH2_), 7.14 (s, 2H, N*H*_2_), 7.30–7.37 (m, 2H, 3‐*H*_FPhe_, 5‐*H*_FPhe_), 7.41–7.46 (m, 1H, 6‐*H*_FPhe_), 7.57 (m, 1H, 4‐*H*_FPhe_). ^13^C NMR (151 MHz, DMSO‐d_6_): δ (ppm) = 55.8 (*CH*_3_), 113.7 (C‐5_PhNH2_), 114.0 (C‐4_PhNH2_), 115.8 (d, *J* = 21.4 Hz, C‐3_FPhe_), 116.0 (C‐1_PhNH2_), 124.6 (d, *J* = 3.3 Hz, C‐5_FPhe_), 124.9 (C‐6_PhNH2_), 128.6 (d, *J* = 17.0 Hz, C‐1_FPhe_), 129.3 (d, *J* = 3.5 Hz, C‐6_FPhe_), 131.9 (d, *J* = 8.1 Hz, C‐4_FPhe_), 142.5 (C‐2_PhNH2_), 146.8 (C‐3_PhNH2_), 158.1 (d, *J* = 245.7 Hz, C‐2_FPhe_), 194.3 (C=O). IR (neat): ν̃ (cm^−1^) = 3441 and 3333 (NH_2_), 1620 (C=O). Exact mass (APCI): *m/z* = 246.0928 (calcd 246.0925 for C_14_H_12_FNO_2_ [M+H]^+^).

#### 4.1.4 General Procedure A for the Synthesis of 3‐Substituted 1,4‐Benzodiazepines **8a‐h**, *ent‐* **8b**, **12a**, **12b**, *ent‐* **12a**

N‐Boc‐protected amino acid (1.5 eq) was stirred in anhydrous CH_2_Cl_2_ (5 mL) at 0°C. PCl_3_ (1.8 eq) was added, the ice bath was removed and the mixture was stirred for 16 h. The mixture was heated to 40°C and the volatiles were removed in vacuo to yield the crude oxazolidinedione. The respective 2‐aminobenzophenone (1.0 eq.) was added, followed by anhydrous toluene (5 mL) and trifluoracetic acid (2.0 eq.). The mixture was stirred for 1 h at 60°C. During this step, the flask was connected to a bubbler filled with toluene to allow CO_2_ to leave the reaction. The end of CO_2_ production indicated the end of the transformation. Then, Et_3_N (2.0 eq.) was added. The mixture was stirred at 80°C for an additional hour. After cooling to room temperature, the mixture was concentrated in vacuo. The residue was purified by flash chromatography (SiO_2_, cyclohexane/ethyl acetate, gradient 100:0 → 0:100).


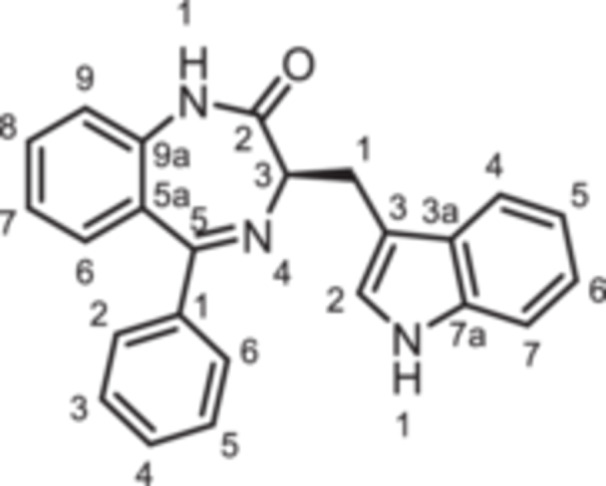


(3*R*)−3‐[(1*H*‐Indol‐3‐yl)methyl]−5‐phenyl‐1,3‐dihydro‐1,4‐benzodiazepin‐2‐one (**8a**): 1,4‐Benzodiazepine **8a** was prepared according to the General Procedure A. N‐Boc‐(*R*)‐tryptophane (**6**, 457 mg, 1.50 mmol) was reacted with PCl_3_ (0.16 mL, 247 mg, 1.80 mmol) and subsequently with 2‐aminobenzophenone **4a** (197 mg,1.00 mmol). Colorless solid, mp > 300°C, yield 256 mg (0.5 mmol, 50%). Purity (HPLC): 95.3% (t_R_ = 17.9 min). Chemical formula: C_24_H_19_N_3_O (365.4 g/mol). TLC: R_f_ = 0.25 (cyclohexane/ethyl acetate 60:40). ^1^H NMR (600 MHz, DMSO‐d_6_): δ (ppm) = 3.46 (dd, *J* = 14.5/8.1 Hz, 1H, C*H*_2_), 3.54 (dd, *J* = 14.5/5.4 Hz, 1H, C*H*_2_), 3.66 (dd, *J* = 8.1/5.4 Hz, 1H, 3‐*H*_bdz_), 6.95 (ddd, *J* = 8.1/7.0/1.1 Hz, 1H, 5‐*H*_indole_), 7.04 (ddd, *J* = 8.0/6.9/1.2 Hz, 1H, 6‐*H*_indole_), 7.12 (td, *J* = 7.6/1.2 Hz, 1H, 7‐*H*_bdz_), 7.18 (m, 2H, 6‐*H*_bdz,_ 2‐*H*_indole_), 7.24 (dd, *J* = 8.2/1.2 Hz, 1H, 9‐*H*_bdz_), 7.32 (d, *J* = 8.1 Hz, 1H, 7‐*H*_indole_), 7.37 – 7.44 (m, 4H, 2‐*H*_Phe,_ 3‐*H*_Phe,_ 5‐*H*_Phe,_ 6‐*H*_Phe_), 7.48 (m, 1H, 4‐*H*_Phe_), 7.54 (ddd, *J* = 8.5/7.2/1.6 Hz, 1H, 8‐*H*_bdz_), 7.58 (d, *J* = 7.9 Hz, 1H, 4‐*H*_indole_), 10.63 (s, 1H, N*H*_bdz_), 10.80 (s, 1H, N*H*_indole_). ^13^C NMR (151 MHz, DMSO‐d_6_): δ (ppm) = 26.7 (C*H*_2_), 64.5 (C‐3_bdz_), 111.3 (C‐7_indole_), 111.4 (C‐3_indole_), 118.1 (C‐5_indole_), 118.6 (C‐4_indole_), 120.8 (C‐6_indole_), 121.1 (C‐9_bdz_), 122.7 (C‐7_bdz_), 123.6 (C‐2_indole_), 126.3 (C‐5a_bdz_), 127.5 (C‐3a_indole_), 128.2 (2C, C‐2_Phe_, C‐6_Phe_), 129.4 (2 C, C‐3_Phe_, C‐5_Phe_), 130.2 (C‐4_Phe_), 130.4 (C‐6_bdz_), 131.6 (C‐8_bdz_), 136.0 (C‐7a_indole_), 138.7 (C‐1_Phe_), 139.2 (C‐9a_bdz_), 167.7 (C‐5_bdz_), 170.4 (C = O). IR (neat): ν̃ (cm^−1^) = 3051 (C‐H, aryl), 1678 (C = O, amide), 1601 (C = C, aryl), 1199 (C‐N, amide). Exact mass (APCI): *m/z* = 366.1601 (calcd 366.1601 for C_24_H_20_N_3_O [M+H]^+^). Specific rotation: ${\left[\alpha \right]}_{20}^{\text{D}}$ = −52.9 (c = 0.09; CH_3_OH).


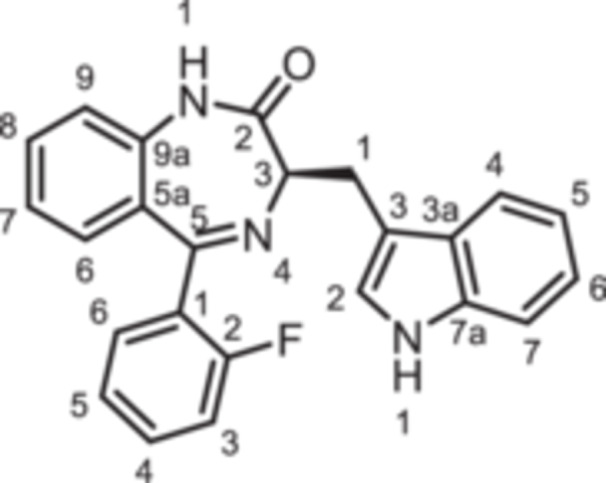


(3*R*)−5‐(2‐Fluorophenyl)−3‐[(1*H*‐indol‐3‐yl)methyl]−1,3‐dihydro‐1,4‐benzodiazepin‐2‐one (**8b**): 1,4‐Benzodiazepine **8b** was prepared according to the General Procedure A. N‐Boc‐(*R*)‐tryptophane (**6**, 457 mg, 1.50 mmol) was reacted with PCl_3_ (0.16 mL, 247 mg, 1.80 mmol) and subsequently with 2‐aminobenzophenone **4b** (215 mg,1.00 mmol). Colorless solid, mp 234°C, yield 203 mg (0.53 mmol, 53%). Purity (HPLC): 99.3% (t_R_ = 19.0 min). Chemical formula: C_24_H_18_FN_3_O (383.4 g/mol). TLC: R_f_ = 0.25 (cyclohexane/ethyl acetate 60:40). ^1^H NMR (600 MHz, DMSO‐d_6_): δ (ppm) = 3.43 (dd, *J* = 14.6/7.9, Hz 1H, C*H*_2_), 3.56 (dd, *J* = 14.6/5.6 Hz, 1H, C*H*_2_), 3.66 (dd, *J* = 7.9/5.6 Hz, 1H, 3‐*H*_bdz_), 6.94 (ddd, *J* = 7.9/7.0/1.0 Hz, 1H, 5‐*H*_indole_), 7.04 (ddd, *J* = 8.2/6.9/1.2 Hz, 1H, 6‐*H*_indole_), 7.06–7.09 (m, 2H, 8‐*H*_bdz_, 7‐*H*_bdz_), 7.16 (d, *J* = 2.3 Hz, 1H, 2‐*H*_indole_), 7.18–7.23 (m, 2H, 9‐*H*_bdz_, 3‐*H*_FPhe_), 7.27 (td, *J* = 7.5/1.1 Hz, 1H, 5‐*H*_FPhe_), 7.32 (dt, *J* = 8.1/1.0 Hz, 1H, 7‐*H*_indole_), 7.44 (td, *J* = 7.6/1.9 Hz, 1H, 6‐*H*_FPhe_), 7.51 (m, 2H, 4‐*H*_FPhe_, 6‐*H*_bdz_), 7.56 (d, *J* = 7.9 Hz, 1H, 4‐*H*_indole_), 10.68 (s, 1H, N*H*_bdz_), 10.81 (s, 1H, N*H*_indole_). ^13^C NMR (151 MHz, DMSO‐d_6_): δ (ppm) = 26.7 (C*H*_2_), 64.6 (C‐3_bdz_), 111.2 (C‐7_indole_), 111.3 (C‐3_indole_), 115.9 (d, *J* = 21.5 Hz, C‐3_FPhe_), 118.1 (C‐5_indole_), 118.6 (C‐4_indole_), 120.8 (C‐6_indole_), 121.1 (C‐9_bdz_), 123.1 (C‐7_bdz_), 123.6 (C‐2_indole_), 124.4 (d, *J* = 3.3 Hz, C‐5_FPhe_), 127.4 (d, *J* = 12.7 Hz, C‐1_FPhe_), 127.4 (C‐3a_indole_), 127.6 (C‐5a_bdz_), 129.1 (C‐8_bdz_), 131.6 (2 C, C‐6_bdz_, C‐4_FPhe_), 132.1 (d, *J* = 8.2 Hz, C‐6_FPhe_), 136.0 (C‐7a_indole_), 138.0 (C‐9a_bdz_), 159.7 (d, *J* = 248.9 Hz, C‐2_FPhe_), 164.5 (C‐5_bdz_), 169.7 (C=O). IR (neat): ν̃ (cm^−1^) = 3051 (C–H, aryl), 1667 (C=O, amide), 1605 (C=C, aryl), 1196 (C–N, amide). Exact mass (APCI): *m/z* = 384.1503 (calcd 384.1507 for C_24_H_19_FN_3_O [M+H]^+^). Specific rotation: ${\left[\alpha \right]}_{20}^{\text{D}}$ = +36.8 (c = 0.12; CH_3_OH).


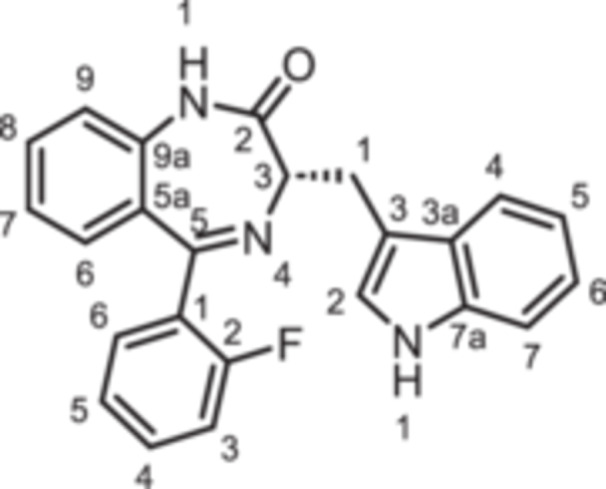


(3*S*)−5‐(2‐Fluorophenyl)−3‐[(1*H*‐indol‐3‐yl)methyl]−1,3‐dihydro‐1,4‐benzodiazepin‐2‐one (*ent*‐**8b**): 1,4‐Benzodiazepine *ent‐* **8b** was prepared according to the General Procedure A. N‐Boc‐(*S*)‐tryptophane (*ent‐* **6**, 457 mg, 1.50 mmol) was reacted with PCl_3_ (0.16 mL, 247 mg, 1.80 mmol) and subsequently with 2‐aminobenzophenone **4b** (215 mg, 1.00 mmol). Colorless solid, mp 234°C, yield 268 mg (0.70 mmol, 70%). Purity (HPLC): 98.3% (t_R_ = 19.0 min). Chemical formula: C_24_H_18_FN_3_O (383.4 g/mol). TLC: R_f_ = 0.25 (cyclohexane/ethyl acetate 60:40). ^1^H NMR, ^13^C NMR, and IR spectra are identical with the spectra of enantiomer **8b**. Exact mass (APCI): *m/z* = 384.1506 (calcd 384.1507 for C_24_H_19_FN_3_O [M+H]^+^). Specific rotation: ${\left[\alpha \right]}_{20}^{\text{D}}$ = −39.0 (c = 0.24; CH_3_OH).


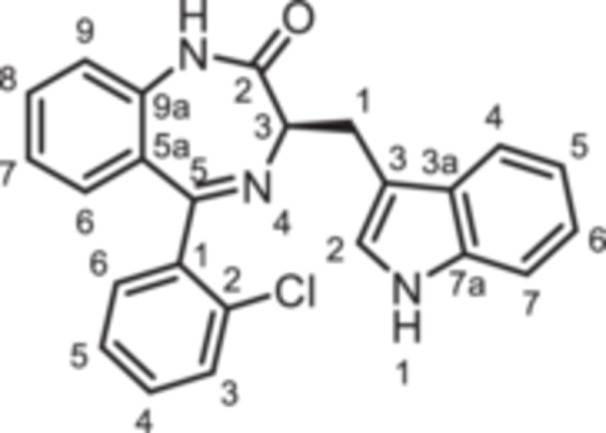


(3*R*)−5‐(2‐Chlorophenyl)−3‐[(1*H*‐indol‐3‐yl)methyl]−1,3‐dihydro‐1,4‐benzodiazepin‐2‐one (**8c**): 1,4‐Benzodiazepine **8c** was prepared according to the General Procedure A. N‐Boc‐(*R*)‐tryptophane (**6**, 1.83 g, 6.01 mmol) was reacted with PCl_3_ (0.63 mL, 991 mg, 7.22 mmol) and subsequently with 2‐aminobenzophenone **4c** (0.93 g, 4.00 mmol). Colorless solid, mp 247°C, yield 240 mg (0.60 mmol, 15%). Purity (HPLC): 98.4% (t_R_ = 20.0 min). Chemical formula: C_24_H_18_ClN_3_O (399.9 g/mol). TLC: R_f_ = 0.22 (cyclohexane/ethyl acetate 60:40). ^1^H NMR (600 MHz, DMSO‐d_6_): δ (ppm) = 3.40 (dd, *J* = 14.6/7.7 Hz, 1H, C*H*_2_), 3.57 (dd, *J* = 14.6/5.9 Hz, 1H, C*H*_2_), 3.70 (t, *J* = 6.7 Hz, 1H, 3‐*H*,_bdz_), 6.91–6.94 (m, 2H, 5‐*H*_ClPhe_, 5‐*H*_indole_), 7.02–7.06 (m, 2H, 7‐*H*_bdz_, 6‐*H*_indole_), 7.14 (d, *J* = 2.3 Hz, 1H, 2‐*H*_indole_), 7.19 (dd, *J* = 8.2/1.1 Hz, 1H, 9‐*H*_bdz_), 7.32 (dt, *J* = 8.1/0.9 Hz, 1H, 7‐*H*_indole_), 7.38 (dd, *J* = 7.6/1.8 Hz, 1H, 4‐*H*_ClPhe_), 7.41–7.51 (m, 4H, 6‐*H*_bdz_, 8‐*H*_bdz_, 3‐*H*_ClPhe_, 6‐*H*_ClPhe_), 7.55 (m, 1H, 4‐*H*_indole_), 10.72 (s, 1H, N*H*_bdz_), 10.80 (s, 1H, N*H*_indole_). ^13^C NMR (151 MHz, DMSO‐d_6_): δ (ppm) = 26.3 (C*H*_2_), 64.5 (C‐3_bdz_), 111.3 (C‐3_indol_), 111.3 (C‐7_indol_), 118.1 (C‐5_indol_), 118.6 (C‐4_indol_), 120.8 (C‐6_indol_), 121.0 (C‐9_bdz_), 123.0 (C‐7_bdz_), 123.7 (C‐2_indole_, 127.1 (C‐5a_bdz_), 127.3 (C‐2_ClPhe_), 127.4 (C‐3a_indole_), 129.0 (C‐5_ClPhe_), 129.6 (C‐6_ClPhe_), 130.9 (C‐6_bdz_), 131.4 (C‐4_ClPhe_), 131.6 (C‐8_bdz_), 132.0 (C‐3_ClPhe_), 136.0 (C‐7a_indole_), 138.5 (C‐9a_bdz),_ 138.8 (C‐1_ClPhe_), 167.3 (C‐5_bdz_), 169.6 (C=O). IR (neat): ν̃ (cm^−1^) = 3059 (C–H, aryl), 1674 (C=O, amide), 1609 (C=C, aryl), 1161 (C–N, amide). Exact mass (APCI): *m/z* = 400.1208 (calcd 400.1211 for C_24_H_19_ClN_3_O [M+H]^+^). Specific rotation: ${\left[\alpha \right]}_{20}^{\text{D}}$ = +93.9 (c = 0.17; CH_3_OH).


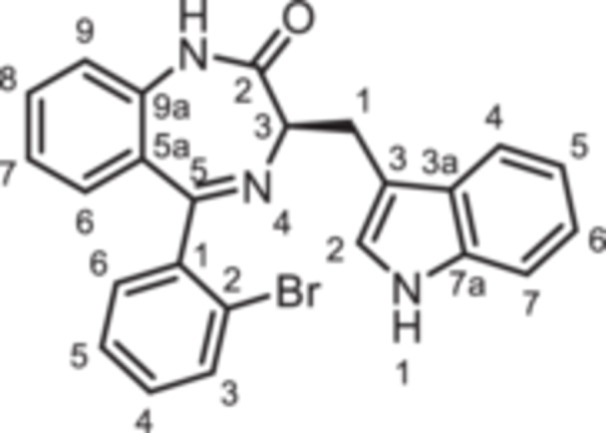


(3*R*)−5‐(2‐Bromophenyl)−3‐[(1*H*‐indol‐3‐yl)methyl]−1,3‐dihydro‐1,4‐benzodiazepin‐2‐one (**8d**): 1,4‐Benzodiazepine **8d** was prepared according to the General Procedure A. N‐Boc‐(*R*)‐tryptophane (**6**, 1.83 g, 6.01 mmol) was reacted with PCl_3_ (0.63 mL, 991 mg, 7.22 mmol) and subsequently with 2‐aminobenzophenone **4d** (1.11 g,4.01 mmol). Colorless solid, mp 238°C, yield 231 mg (0.52 mmol, 13%). Purity (HPLC): 98.4% (t_R_ = 20.2 min). Chemical formula: C_24_H_18_BrN_3_O (444.3 g/mol). TLC: R_f_ = 0.18 (cyclohexane/ethyl acetate 60:40). ^1^H NMR (600 MHz, DMSO‐d_6_): δ (ppm) = 3.38 (dd, *J* = 14.6/7.7 Hz, 1H, C*H*_2_), 3.57 (dd, *J* = 14.6/6.1 Hz, 1H, C*H*_2_), 3.69 (t, *J* = 6.7 Hz, 1H, 3‐*H*_bdz_), 6.95–6.88 (m, 2H, 5‐*H*_indole_, 6‐*H*_BrPhe_), 7.02–7.05 (m, 2H, 7‐*H*_bdz_, 6‐*H*_indole_), 7.14 (d, *J* = 2.3 Hz, 1H, 2‐*H*_indole_), 7.18 (dd, *J* = 8.3/1.2 Hz, 1H, 9‐*H*_bdz_), 7.32 (dt, *J* = 8.3/0.9 Hz, 1H, 7‐*H*_indole_), 7.34–7.41 (m, 2H, 4‐*H*_BrPhe_, 6‐*H*_bdz_), 7.46–7.50 (m, 2H, 5‐*H*_BrPhe_, 8‐*H*_bdz_), 7.54 (dd, *J* = 7.9/1.1 Hz, 1H, 4‐*H*_indole_), 7.62 (dd, *J* = 7.9/1.1 Hz, 1H, 3‐*H*_BrPhe_), 10.74 (s, 1H, N*H*_bdz_), 10.80 (s, 1H, N*H*_indole_). ^13^C NMR (151 MHz, DMSO‐d_6_): δ (ppm) = 26.7 (C*H*_2_), 64.4 (C‐3_bdz_), 111.3 (C‐3_indole_), 111.3 (C‐7_indole_), 118.1 (C‐5_indole_), 118.6 (C‐4_indole_), 120.8 (C‐6_indole_), 121.0 (C‐9_bdz_), 121.7 (C‐2_BrPhe_), 123.0 (C‐7_bdz_), 123.7 (C‐2_indole_), 127.1 (C‐5a_bdz_), 127.4 (C‐3a_indole_), 127.6 (C‐5_BrPhe_), 129.1 (C‐6_BrPhe_), 131.0 (C‐6_bdz_), 131.4 (C‐4_BrPhe_), 131.6 (C‐8_bdz_), 132.8 (C‐3_BrPhe_), 136.0 (C‐7a_indole_), 138.8 (C‐1_BrPhe_), 140.7 (C‐9a_bdz_), 168.4 (C‐5_bdz_), 169.5 (C=O). IR (neat): ν̃ (cm^−1^) = 3055 (C–H, aryl), 1678 (C=O, amide), 1605 (C=C, aryl), 1161 (C–N, amide). Exact mass (APCI): *m/z* = 444.0710 (calcd 444.0706 for C_24_H_19_^79^BrN_3_O [M+H]^+^). Specific rotation: ${\left[\alpha \right]}_{20}^{\text{D}}$ = +97.0 (c = 0.14; CH_3_OH).


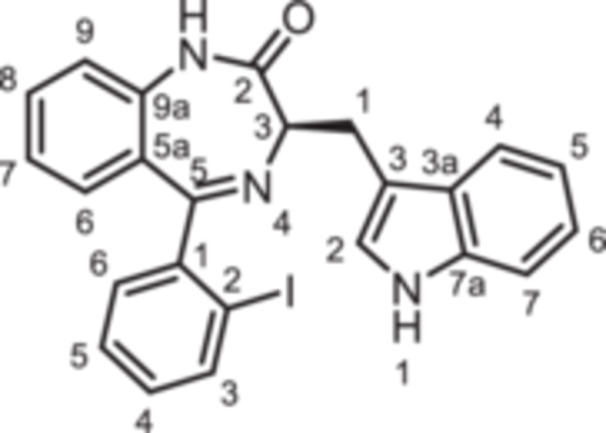


(3*R*)−3‐[(1*H*‐Indol‐3‐yl)methyl]−5‐(2‐iodophenyl)−1,3‐dihydro‐1,4‐benzodiazepin‐2‐one (**8e**): 1,4‐Benzodiazepine **8e** was prepared according to the General Procedure A. N‐Boc‐(*R*)‐tryptophane (**6**, 1.83 g, 6.01 mmol) was reacted with PCl_3_ (0.63 mL, 991 mg, 7.22 mmol) and subsequently with 2‐aminobenzophenone **4e** (1.29 g, 4.00 mmol). Colorless solid, mp 248°C, yield 138 mg (0.28 mmol, 7%). Purity (HPLC): 99.8% (t_R_ = 20.3 min). Chemical formula: C_24_H_18_IN_3_O (491.3 g/mol). TLC: R_f_ = 0.17 (cyclohexane/ethyl acetate 70:30). ^1^H NMR (600 MHz, DMSO‐d_6_): δ (ppm) = 3.38 (dd, *J* = 13.6/6.8 Hz, 1H, C*H*_2_), 3.60 (dd, *J* = 14.5/6.5, 1H, C*H*_2_), 3.68 (t, *J* = 6.8 Hz, 1H, 3‐*H*_bdz_), 6.87 (dd, *J* = 7.9/1.5 Hz, 1H, 8‐*H*_bdz_), 6.92 (ddd, *J* = 8.0/7.0/1.0 Hz, 1H, 5‐*H*_indole_), 7.00 – 7.07 (m, 2H, 7‐*H*_bdz_, 6‐*H*_indole_), 7.15 (d, *J* = 2.3 Hz, 1H, 2‐*H*_indole_), 7.17–7.21 (m, 2H, 9‐*H*,_bdz_, 4‐*H*_IPhe_), 7.28 (dd, *J* = 7.6/1.7 Hz, 1H, 6‐*H*_IPhe_), 7.31 (dd, *J* = 8.1/0.9 Hz, 1H, 7‐*H*_indole_), 7.45–7.51 (m, 2H, 6‐*H*_bdz_, 5‐*H*_IPhe_), 7.54 (d, *J* = 7.9 Hz, 1H, 4‐*H*_indole_), 7.87 (dd, *J* = 7.9/1.1 Hz, 1H, 3‐*H*_IPhe_), 10.78 (s, 1H, N*H*_bdz_), 10.80 (s, 1H, N*H*_indole_). ^13^C NMR (151 MHz, DMSO‐d_6_): δ (ppm) = 26.7 (C*H*_2_), 64.5 (C‐3_bdz_), 97.3 (C‐2_IPhe_), 111.3 (C‐7_indole_), 111.3 (C‐3_indole_), 118.1 (C‐5_indole_), 118.6 (C‐4_indole_), 120.8 (C‐6_indole_), 120.9 (C‐9_bdz_), 122.9 (C‐7_bdz_), 123.7 (C‐2_indole_), 126.6 (C‐5a_bdz_), 127.4 (C‐3a_indole_), 128.0 (C‐5_IPhe_), 129.4 (C‐8_bdz_), 130.7 (C‐4_IPhe_), 130.9 (C‐6_IPhe_), 131.7 (C‐6_bdz_), 136.0 (C‐7a_indole_), 139.2 (C‐3_IPhe_), 139.5 (C‐9a_bdz_), 144.3 (C‐1_IPhe_), 169.3 (C‐5_bdz_), 170.4 (C=O). IR (neat): ν̃ (cm^−1^) = 3051 (C–H, aryl), 1678 (C=O, amide), 1605 (C=C, aryl), 1161 (C–N, amide). Exact mass (APCI): *m/z* = 492.0543 (calcd 492.0567 for C_24_H_19_IN_3_O [M+H]^+^). Specific rotation: ${\left[\alpha \right]}_{20}^{\text{D}}$ = +97.9 (c = 0.19; CH_3_OH).


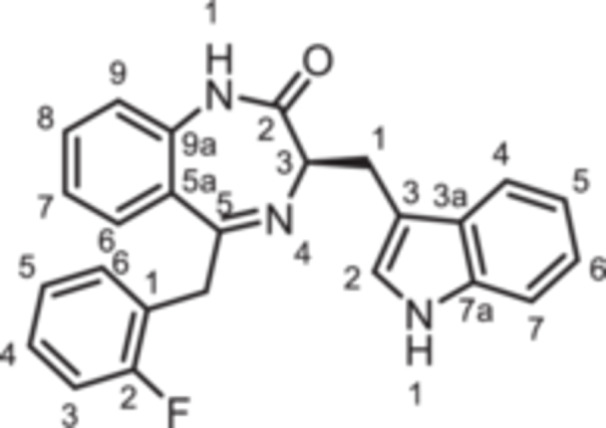


(3*R*)−5‐(2‐Fluorobenzyl)−3‐[(1*H*‐indol‐3‐yl)methyl]−1,3‐dihydro‐1,4‐benzodiazepin‐2‐one (**8f**): 1,4‐Benzodiazepine **8f** was prepared according to the General Procedure A. N‐Boc‐(*R*)‐tryptophane (**6**, 913 mg, 3.00 mmol) was reacted with PCl_3_ (0.31 mL, 494 mg, 3.60 mmol) and subsequently with 2‐aminobenzophenone **4 f** (459 mg, 2.00 mmol). Colorless solid, mp 129°C–130°C, yield 167 mg (0.42 mmol, 21%). Purity (HPLC): 97.4% (t_R_ = 19.0 min). Chemical formula: C_25_H_20_FN_3_O (397.5 g/mol). TLC: R_f_ = 0.28 (cyclohexane/ethyl acetate 60:40). ^1^H NMR (600 MHz, DMSO‐d_6_): δ (ppm) = 3.23 (dd, *J* = 14.5/7.7 Hz, 1H, C*H*_2indole_), 3.43 (dd, *J* = 14.5/5.7 Hz, 1H, C*H*_2indole_), 3.50 (t, *J* = 6.8 Hz, 1H, 3‐*H*_bdz_), 4.03 (d, *J* = 15.8 Hz, 1H, C*H*_2benzyl_), 4.18 (d, *J* = 15.8 Hz, 1H, C*H*_2benzyl_), 6.88 (ddd, *J* = 8.0/7.0/1.0 Hz, 1H, 5‐*H*_indole_), 6.95–7.14 (m, 7H, 2‐*H*_indole_, 6‐*H*_indole_, 3‐*H*_FPhe_, 5‐*H*_FPhe_, 7‐*H*_bdz_, 8‐*H*_bdz_, 9‐*H*_bdz_), 7.18–7.25 (m, 1H, 4‐*H*_FPhe_), 7.29 (dt, *J* = 8.1/0.9 Hz, 1H, 7‐*H*_indole_), 7.40 (ddd, *J* = 8.2/7.2/1.4 Hz, 1H, 6‐*H*_FPhe_), 7.44 (dd, *J* = 7.9/1.1 Hz, 1H, 4‐*H*_indole_), 7.71 (dd, *J* = 8.0/1.5 Hz, 1H, 6‐*H*_bdz_), 10.42 (s, 1H, N*H*_bdz_), 10.75 (s, 1H, N*H*_indole_). ^13^C NMR (151 MHz, DMSO‐d_6_): δ (ppm) = 26.6 (C*H*_2indole_), 37.3 (C*H*_2benzyl_), 63.8 (C‐3_bdz_), 111.2 (C‐7_indole_), 111.3 (C‐3_indole_), 115.1 (d, *J* = 21.9 Hz, C‐3_FPhe_), 118.1 (C‐5_indole_), 118.4 (C‐4_indole_), 120.7 (C‐6_indole_), 121.0 (C‐9_bdz_), 123.1 (C‐7_bdz_), 123.7 (C‐2_indole_), 124.1 (d, *J* = 3.4 Hz, C‐5_FPhe_), 124.6 (d, *J* = 15.6 Hz, C‐1_FPhe_), 127.2 (C‐3a_indole_), 127.4 (C‐5a_bdz_), 128.1 (C‐6_bdz_), 128.4 (d, *J* = 8.1 Hz, C‐4_FPhe_), 131.1 (d, *J* = 3.9 Hz, C‐6_FPhe_), 131.1 (C‐8_bdz_), 135.9 (C‐7a_indole_), 137.9 (C‐9a_bdz_), 160.3 (d, *J* = 244.1 Hz, C‐2_FPhe_), 167.5 (C‐5_bdz_), 170.1 (C=O). IR (neat): ν̃ (cm^−1^) = 3058 (C‐H, aryl), 1678 (C=O, amide), 1620 (C=C, aryl), 1184 (C–N, amide). Exact mass (APCI): *m/z* = 398.1666 (calcd 398.1663 for C_25_H_21_FN_3_O [M+H]^+^). Specific rotation: ${\left[\alpha \right]}_{20}^{\text{D}}$ = +61.7 (c = 0.26; CH_3_OH).


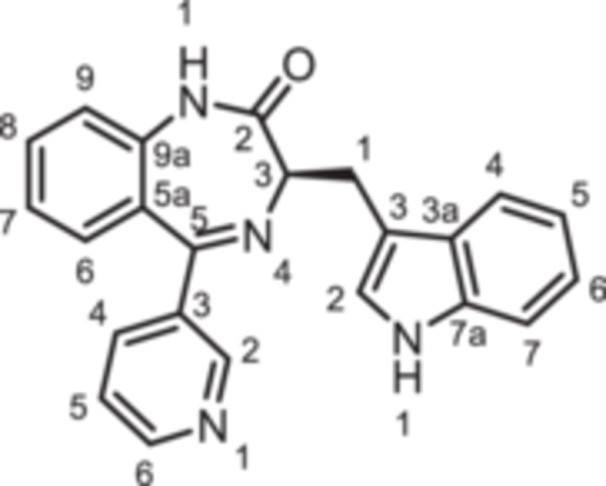


(3*R*)−3‐[(1*H*‐Indol‐3‐yl)methyl]−5‐(pyridin‐3‐yl)−1,3‐dihydro‐1,4‐benzodiazepin‐2‐one (**8g**): 1,4‐Benzodiazepine **8g** was prepared according to the General Procedure A. N‐Boc‐(*R*)‐tryptophane (**6**, 913 mg, 3.00 mmol) was reacted with PCl_3_ (0.31 mL, 494 mg, 3.60 mmol) and subsequently with 2‐aminobenzophenone **4 g** (396.4 mg, 2.00 mmol). Yellow solid, mp 222°C–223°C, yield 169 mg (0.46 mmol, 23%). Purity (HPLC): 97.6% (t_R_ = 15.5 min). Chemical formula: C_23_H_18_N_4_O (366.4 g/mol). TLC: R_f_ = 0.14 (cyclohexane/ethyl acetate 10:90). ^1^H NMR (600 MHz, DMSO‐d_6_): δ (ppm) = 3.50 (dd, *J* = 14.5/7.9, 1H, C*H*_2_), 3.53 (dd, *J* = 14.5/5.3 Hz, 1H, C*H*_2_), 3.67 (dd, *J* = 7.9/5.3 Hz, 1H, 3‐*H*_bdz_), 6.94 (ddd, *J* = 8.0/7.0/1.1 Hz, 1H, 5‐*H*_indole_), 7.04 (ddd, *J* = 8.1/6.9/1.2 Hz, 1H, 6‐*H*_indole_), 7.14 (ddd, *J* = 8.0/7.2/1.2 Hz, 1H, 7‐*H*_bdz_), 7.18–7.23 (m, 2H, 2‐*H*_indole_, 6‐*H*_bdz_), 7.25 (dd, *J* = 8.2/1.3 Hz, 1H, 9‐*H*_bdz_), 7.31 (dt, *J* = 8.1/1.0 Hz, 1H, 7‐*H*_indole_), 7.43 (ddd, *J* = 7.9/4.8/0.9 Hz, 1H, 5‐*H*_pyridine_), 7.53–7.60 (m, 2H, 8‐*H*_bdz_, 4‐*H*_indole_), 7.75 (dt, *J* = 8.0/1.9 Hz, 1H, 4‐*H*_pyridine_), 8.58 (dd, *J* = 2.3/0.8 Hz, 1H, 2‐*H*_pyridine_), 8.65 (dd, *J* = 4.8/1.7 Hz, 1H, 6‐*H*_pyridine_), 10.69 (s, 1H, N*H*_bdz_), 10.81 (s, 1H, N*H*_indole_). ^13^C NMR (151 MHz, DMSO‐d_6_): δ (ppm) = 26.8 (C*H*_2_), 64.8 (C‐3_bdz_), 111.3 (C‐3_indole_), 111.3 (C‐7_indole_), 118.1 (C‐5_indole_), 118.6 (C‐4_indole_), 120.8 (C‐6_indole_), 121.3 (C‐9_bdz_), 122.9 (C‐7_bdz_), 123.3 (C‐5_pyridine_), 123.7 (C‐2_indole_), 125.7 (C‐5a_bdz_), 127.5 (C‐3a_indole_), 130.2 (C‐6_bdz_), 131.9 (C‐8_bdz_), 134.4 (C‐3_pyridine_), 136.0 (C‐7a_indole_), 136.7 (C‐4_pyridine_), 139.3 (C‐9a_bdz_), 149.8 (C‐2_pyridine_), 150.9 (C‐6_pyridine_), 165.7 (C‐5_bdz_), 170.2 (C=O). IR (neat): ν̃ (cm^−1^) = 3051 (C–H, aryl), 1666 (C=O, amide), 1601 (C=C, aryl), 1195 (C–N, amide). Exact mass (APCI): *m/z* = 367.1551 (calcd 367.1553 for C_23_H_19_N_4_O [M+H]^+^). Specific rotation: ${\left[\alpha \right]}_{20}^{\text{D}}$ = −60.8 (c = 0.17; CH_3_OH).


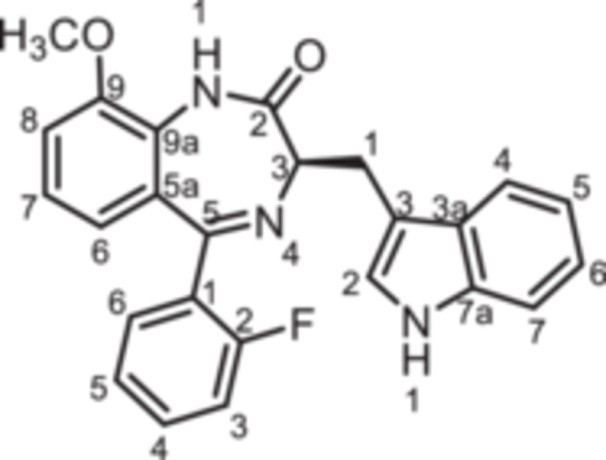


(3*R*)−5‐(2‐Fluorophenyl)−3‐[(1*H*‐indol‐3‐yl)methyl]−9‐methoxy‐1,3‐dihydro‐1,4‐benzodiazepin‐2‐one (**8h**): 1,4‐Benzodiazepine **8h** was prepared according to the General Procedure A. N‐Boc‐(*R*)‐tryptophane (**6**, 4.57 g, 15.00 mmol) was reacted with PCl_3_ (1.57 mL, 2.47 g, 18.00 mmol) and subsequently with 2‐aminobenzophenone **4 h** (2.45 g, 10.00 mmol). Colorless solid, mp 189°C–190°C, yield 1.78 g (4.30 mmol, 43%). Purity (HPLC): 97.9% (t_R_ = 19.9 min). Chemical formula: C_25_H_20_FN_3_O_2_ (413.5 g/mol). TLC: R_f_ = 0.34 (cyclohexane/ethyl acetate 50:50). ^1^H NMR (600 MHz, DMSO‐d_6_): δ (ppm) = 3.42 (dd, *J* = 14.4/7.5 Hz, 1H, C*H*_2_), 3.57 (dd, *J* = 14.4/6.0 Hz, 1H, C*H*_2_), 3.63 (t, *J* = 6.7 Hz, 1H, 3‐*H*_bdz_), 3.85 (s, 3H, OC*H*_3_), 6.63 (dd, *J* = 8.1/1.2 Hz, 1H, 8‐*H*_bdz_), 6.93 (td, *J* = 7.9/1.0 Hz, 1H, 5‐*H*_indole_), 7.01–7.10 (m, 2H, 6‐*H*_indole_, 7‐*H*_bdz_), 7.12–7.18 (m, 2H, 6‐*H*_bdz_, 2‐*H*_indole_), 7.21 (ddd, *J* = 10.7/8.3/1.1 Hz, 1H, 3‐*H*_FPhe_), 7.28 (td, *J* = 7.5/1.1 Hz, 1H, 5‐*H*_FPhe_), 7.32 (dt, *J* = 8.2/0.9 Hz, 1H, 7‐*H*_indole_), 7.46 (td, *J* = 7.6/1.9 Hz, 1H, 6‐*H*_FPhe_), 7.51–7.59 (m, 2H, 4‐*H*_FPhe_, 4‐*H*_indole_), 9.83 (s, 1H, N*H*_bdz_), 10.82 (d, *J* = 2.4 Hz, 1H, N*H*_indole_). ^13^C NMR (151 MHz, DMSO‐d_6_): δ (ppm) = 26.6 (C*H*_2_), 55.9 (O*C*H_3_), 64.9 (C‐3_bdz_), 111.3 (C‐3 _indole_, C‐7_indole_), 112.8 (C‐6_bdz_), 115.9 (d, *J* = 21.4 Hz, C‐3_FPhe_), 118.1 (C‐5_indole_), 118.6 (C‐4_indole_), 120.0 (C‐8_bdz_), 120.8 (C‐6_indole_), 123.7 (C‐2_indole_), 124.0 (C‐7_bdz_), 124.5 (d, *J* = 3.3 Hz, C‐5_FPhe_), 127.1 (C‐9a_bdz_), 127.3 (d, *J* = 12.3 Hz, C‐1_FPhe_), 127.4 (C‐3a_indole_), 129.0 (C‐5a_bdz_), 131.5 (d, *J* = 2.6 Hz, C‐6_FPhe_), 132.2 (d, *J* = 8.4 Hz, C‐4_FPhe_), 136.0 (C‐7a_indole_), 149.6 (C‐9_bdz_), 159.8 (d, *J* = 249.1 Hz, C‐2_FPhe_), 164.3 (C‐5_bdz_), 169 (C = O). IR (neat): ν̃ (cm^−1^) = 3055 (C‐H, aryl), 1662 (C=O, amide), 1605 (C=C, aryl), 1237 (C–O), 1188 (C–N, amide). Exact mass (APCI): *m/z* = 414.1611 (calcd 414.1612 for C_25_H_21_FN_3_O_2_ [M+H]^+^). Specific rotation: ${\left[\alpha \right]}_{20}^{\text{D}}$ = +17.2 (c = 0.20; CH_3_OH).


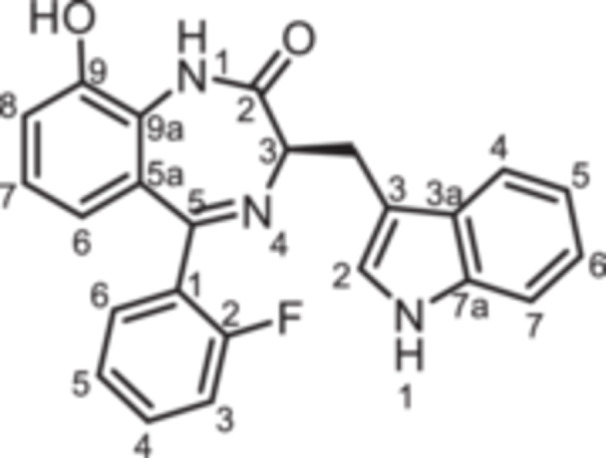


(3*R*)−5‐(2‐Fluorophenyl)−9‐hydroxy‐3‐[(1*H*‐indol‐3‐yl)methyl]−1,3‐dihydro‐1,4‐benzodiazepin‐2‐one (**8i**): At 0°C a solution of BBr_3_ in CH_2_Cl_2_ (1 M, 1.50 mL, 1.50 mmol, 3.13 eq.) was added to a solution of **8h** (200 mg, 0.48 mmol, 1.00 eq.) in CH_2_Cl_2_ (2.00 mL). The ice bath was removed and the mixture was stirred for 3 h at room temperature. Then, CH_3_OH (6.00 mL) was added and the mixture was stirred for 1 h. CH_2_Cl_2_ (30 mL) and water (30 mL) were added and the aqueous layer was extracted with CH_2_Cl_2_ (3 × 30 mL). The organic layer was dried (Na_2_SO_4_), filtered and concentrated in vacuo. The residue was purified by flash chromatography (SiO_2_, cyclohexane/ethyl acetate, gradient 100:0 → 0:100). Colorless solid, mp 184°C–185°C, yield 125 mg (0.31 mmol, 65%). Purity (HPLC): 98.0% (t_R_ = 17.8 min). Chemical formula: C_24_H_18_FN_3_O_2_ (399.4 g/mol). TLC: R_f_ = 0.19 (cyclohexane/ethyl acetate 50:50). ^1^H NMR (600 MHz, DMSO‐d_6_): δ (ppm) = 3.40 (dd, *J* = 14.5/7.6 Hz, 1H, C*H*_2_), 3.56 (dd, *J* = 14.5/5.9 Hz, 1H, C*H*_2_), 3.63 (dd, *J* = 7.6/5.9 Hz, 1H, 3‐*H*_bdz_), 6.50 (dd, *J* = 7.6 Hz, 1H, 8‐*H*_bdz_), 6.88–6.96 (m, 2H, 7‐*H*_bdz_, 5‐*H*_indole_), 6.98 (dd, *J* = 7.9/1.3 Hz, 1H, 6‐*H*_bdz_), 7.04 (ddd, *J* = 8.2/6.9/1.2 Hz, 1H, 6‐*H*_indole_), 7.15 (d, *J* = 2.3 Hz, 1H, 2‐*H*_indole_), 7.20 (ddd, *J* = 10.7/8.3/1.1 Hz, 1H, 3‐*H*_FPhe_), 7.27 (td, *J* = 7.6/1.2 Hz, 1H, 5‐*H*_FPhe_), 7.32 (dt, *J* = 8.1/1.0 Hz, 1H, 7‐*H*_indole_), 7.44 (td, *J* = 7.6/1.9 Hz, 1H, 6‐*H*_FPhe_), 7.49–7.54 (m, 1H, 4‐*H*_FPhe_), 7.56 (dd, *J* = 8.0/1.0 Hz, 1H, 4‐*H*_indole_), 9.59 (s, 1H, O*H*), 10.27 (s, 1H, N*H*_bdz_), 10.81 (d, *J* = 2.3 Hz, 1H, N*H*_indole_). ^13^C NMR (151 MHz, DMSO‐d_6_): δ (ppm) = 26.7 (C*H*_2_), 64.8 (C‐3_bdz_), 111.3 (C‐7_indole_), 111.4 (C‐3_indole_), 115.8 (d, *J* = 21.5 Hz, C‐3_FPhe_), 116.7 (C‐6_bdz_), 118.1 (C‐5_indole_), 118.6 (C‐4_indole_), 118.9 (C‐8_bdz_), 120.8 (C‐6_indole_), 123.7 (C‐2_indole_), 123.9 (C‐7_bdz_), 124.4 (d, *J* = 3.2 Hz, C‐5_FPhe_), 126.3 (C‐9a_bdz_), 127.4 (C‐3a_indole_), 127.6 (d, *J* = 12.5 Hz, C‐1_FPhe_), 129.3 (C‐5a_bdz_), 131.5 (d, *J* = 2.7 Hz, C‐6_FPhe_), 132.0 (d, *J* = 8.3 Hz, C‐4_FPhe_), 136.0 (C‐7a_indole_), 147.9 (C‐9_bdz_), 159.8 (d, *J* = 249.1 Hz, C‐2_FPhe_), 164.5 (C‐5_bdz_), 169.2 (C=O). IR (neat): ν̃ (cm^−1^) = 3206 (O–H), 3055 (C–H, aryl), 1670 (C=O, amide), 1601 (C=C, aryl), 1237 (C–N, amide). Exact mass (APCI): *m/z* = 400.1467 (calcd 400.1456 for C_24_H_19_FN_3_O_2_ [M+H]^+^). Specific rotation: ${\left[\alpha \right]}_{20}^{\text{D}}$ = +7.3 (c = 0.19; CH_3_OH).


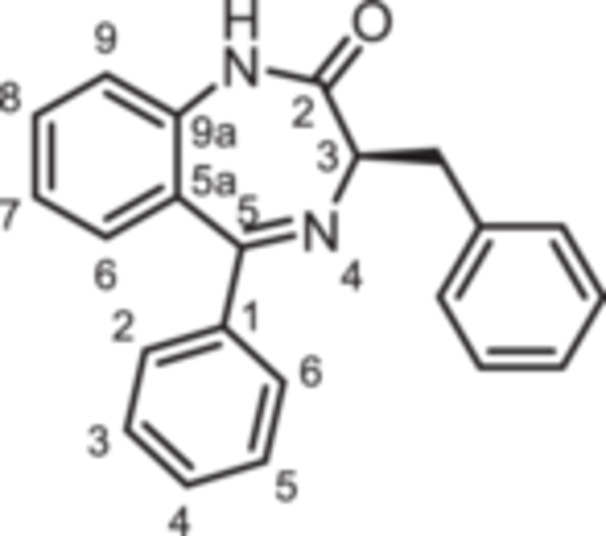


(3*R*)−3‐Benzyl‐5‐phenyl‐1,3‐dihydro‐1,4‐benzodiazepin‐2‐one (**12a**): 1,4‐Benzodiazepine **12a** was prepared according to the General Procedure A. N‐Boc‐(*R*)‐phenylalanine (**10**, 1.60 g, 6.03 mmol) was reacted with PCl_3_ (0.63 mL, 994 mg, 7.24 mmol) and subsequently with 2‐aminobenzophenone **4a** (789 mg, 4.00 mmol). Colorless solid, mp 193°C, yield 878 mg (2.69 mmol, 67%). Purity (HPLC): 98.5% (t_R_ = 18.6 min). Chemical formula: C_22_H_18_N_2_O (326.4 g/mol). TLC: R_f_ = 0.36 (cyclohexane/ethyl acetate 70:30). ^1^H NMR (600 MHz, DMSO‐d_6_): δ (ppm) = 3.36 (dd, *J* = 13.7/8.4 Hz, 1H, C*H*_2_), 3.40 (dd, *J* = 13.7/5.2 Hz, 1H, C*H*_2_), 3.66 (dd, *J* = 8.4/5.2 Hz, 1H, 3‐*H*_bdz_), 7.11–7.22 (m, 3H, 6‐*H*_bdz_, 7‐*H*_bdz_, 4‐*H*_benzyl_), 7.23–7.29 (m, 3H, 9‐*H*_bdz_, 3‐*H*_benzyl_, 5‐*H*_benzyl_), 7.32–.34 (m, 2H, 2‐*H*_benzyl_, 6‐*H*_benzyl_), 7.37–7.44 (m, 4H, 2‐*H*_Phe_, 6‐*H*_Phe_, 3‐*H*_Phe_, 5‐*H*_Phe_), 7.47 (m, 1H, 4‐*H*_Phe_), 7.55 (ddd, *J* = 8.6/7.2/1.6 Hz, 1H, 8‐*H*_bdz_), 10.62 (s, 1H, N*H*). ^13^C NMR (151 MHz, DMSO‐d_6_): δ (ppm) = 37.1 (C*H*_2_), 64.9 (C‐3_bdz_), 121.2 (C‐9_bdz_), 122.7 (C‐7_bdz_), 126.0 (C‐4_benzyl_), 126.4 (C‐5a_bdz_), 128.0 (2 C, C‐3_benzyl_, C‐5_benzyl_), 128.2 (2C, C‐2_Phe_, C‐6_Phe_), 129.3 (2 C, C‐3_Phe_, C‐5_Phe_), 129.7 (2 C, C‐2_benzyl_, C‐6_benzyl_), 130.2 (C‐4_Phe_), 130.4 (C‐6_bdz_), 131.6 (C‐8_bdz_), 138.9 (C‐1_Phe_), 139.1 (C‐9a_bdz_), 139.3 (C‐1_benzyl_), 167.7 (C‐5_bdz_), 170.2 (C=O). IR (neat): ν̃ (cm^−1^) = 3055 (C‐H, aryl), 1678 (C=O, amide), 1601 (C=C, aryl), 1161 (C–N, amide). Exact mass (APCI): *m/z* = 327.1496 (calcd 327.1492 for C_22_H_19_N_2_O [M+H]^+^). Specific rotation: ${\left[\alpha \right]}_{20}^{\text{D}}$ = −44.6 (c = 0.08; CH_3_OH).


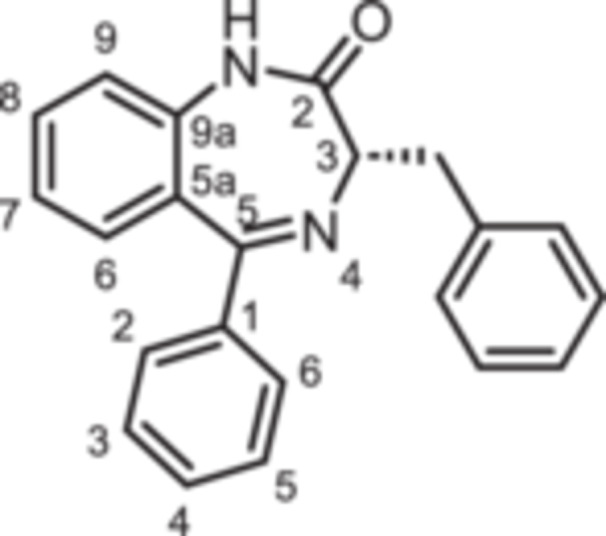


(3*S*)−3‐Benzyl‐5‐phenyl‐1,3‐dihydro‐1,4‐benzodiazepin‐2‐ (e*nt‐* **12a**): 1,4‐Benzodiazepine *ent‐* **12a** was prepared according to the General Procedure A. N‐Boc‐(*S*)‐phenylalanine (*ent*‐**10**, 1.60 g, 6.03 mmol) was reacted with PCl_3_ (0.63 mL, 994 mg, 7.24 mmol) and subsequently with 2‐aminobenzophenone **4a** (789 mg, 4.00 mmol). Colorless solid, mp 193°C, yield 679 mg (2.08 mmol, 52%). Purity (HPLC): 99.7% (t_R_ = 18.6 min). Chemical formula: C_22_H_18_N_2_O (326.4 g/mol). TLC: R_f_ = 0.36 (cyclohexane/ethyl acetate 70:30). ^1^H NMR, ^13^C NMR and IR spectra are identical with the spectra of enantiomer **12a**. Exact mass (APCI): *m/z* = 327.1483 (calcd 327.1492 for C_22_H_19_N_2_O [M+H]^+^). Specific rotation: ${\left[\alpha \right]}_{20}^{\text{D}}$ = +45.7 (c = 0.07; CH_3_OH).


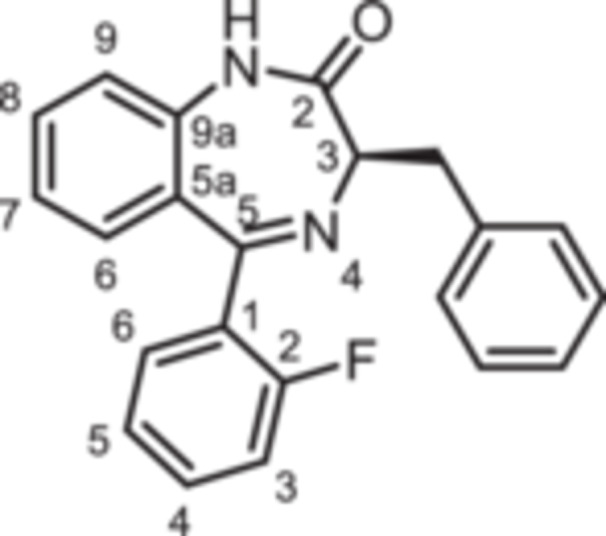


(3*R*)−3‐Benzyl‐5‐(2‐fluorophenyl)−1,3‐dihydro‐1,4‐benzodiazepin‐2‐one (**12b**): 1,4‐Benzodiazepine **12b** was prepared according to the General Procedure A. N‐Boc‐(*R*)‐phenylalanine (**10**, 398 mg, 1.50 mmol) was reacted with PCl_3_ (0.16 mL, 247 mg, 1.80 mmol) and subsequently with 2‐aminobenzophenone **4a** (215 mg,1.00 mmol). Colorless solid, mp 85°C, yield 227 mg (0.66 mmol, 66%). Purity (HPLC): 99.3% (t_R_ = 20.2 min). Chemical formula: C_22_H_17_FN_2_O (344.4 g/mol). TLC: R_f_ = 0.14 (cyclohexane/ethyl acetate 80:20). ^1^H NMR (600 MHz, DMSO‐d_6_): δ (ppm) = 3.34 (dd, *J* = 13.7/8.4 Hz, 1H, C*H*), 3.41 (dd, *J* = 13.7/5.2 Hz, 1H, C*H*), 3.68 (dd, *J* = 8.4/5.2 Hz, 1H, 3‐*H*_bdz_), 7.05–7.12 (m, 2H, 6‐*H*_bdz_, 7‐*H*_bdz_), 7.16–7.24 (m, 3H, 9‐*H*_bdz_, 3‐*H*_FPhe_, 4‐*H*_benzyl_), 7.24–7.33 (m, 5H, 5‐*H*_FPhe_, 2‐*H*_benzyl_, 6‐*H*_benzyl_, 3‐*H*_benzyl_, 5‐*H*_benzyl_), 7.36 (td, *J* = 7.5/1.9 Hz, 1H, 6‐*H*_FPhe_), 7.48–7.56 (m, 2H, 4‐*H*_FPhe_, 8‐*H*_bdz_), 10.70 (s, 1H, N*H*). ^13^C NMR (151 MHz, DMSO‐d_6_): δ (ppm) = 37.0 (C*H*_2_), 65.0 (C‐3_bdz_), 116.0 (d, *J* = 21.4 Hz, C‐3_FPhe_), 121.1 (C‐9_bdz_), 123.1 (C‐7_bdz_), 124.5 (d, *J* = 3.3 Hz, C‐5_FPhe_), 126.0 (C‐4_benzyl_), 127.4 (d, *J* = 12.3 Hz, C‐1_FPhe_), 127.6 (C‐5a_bdz_), 128.0 (2 C, C‐3_benzyl_, C‐5_benzyl_), 129.1 (C‐6_bdz_), 129.6 (2 C, C‐2_benzyl_, C‐6_benzyl_), 131.5 (d, *J* = 2.6 Hz, C‐6_FPhe_), 131.7 (C‐8_bdz_), 132.2 (d, *J* = 8.2 Hz, C‐4_FPhe_), 138.0 (C‐9a_bdz_), 139.2 (C‐1_benzyl_), 159.7 (d, *J* = 249.1 Hz, C‐2_FPhe_), 164.7 (C‐5_bdz_), 169.6 (C=O). IR (neat): ν̃ (cm^−1^) = 3059 (C–H, aryl), 1678 (C=O, amide), 1605 (C=C, aryl), 1103 (C–N, amide). Exact mass (APCI): *m/z* = 345.1382 (calcd 345.1398 for C_22_H_18_FN_2_O [M+H]^+^). Specific rotation: ${\left[\alpha \right]}_{20}^{\text{D}}$ = +34.9 (c = 0.19; CH_3_OH).

#### 4.1.5 General Procedure B for the N‐Methylation of 1,4‐Benzodiazepines **9b‐e**, *ent‐* **9b**, **9h**, **13a,** and *ent*‐**13a**

The respective 1,4‐benzodiazepine **8** or **12** (1.00 eq.) was stirred in anhydrous DMF (4.5 mL) at 0°C. NaH (1.05 eq. as a 60% dispersion in paraffin oil) was added and stirring was continued for 1 h. Then CH_3_I (1.03 eq.) was added and stirring was continued at room temperature for 1 h. The mixture was poured into a saturated solution of NH_4_Cl (10 mL). CH_2_Cl_2_ (30 mL) and H_2_O (20 mL) were added and the product was extracted with CH_2_Cl_2_ (3 × 30 mL). The combined organic layers were dried (Na_2_SO_4_), filtered and concentrated in vacuo. The residue was purified by flash chromatography (SiO_2_, CH_2_Cl_2_/CH_3_OH, gradient 100:0 → 90:10).


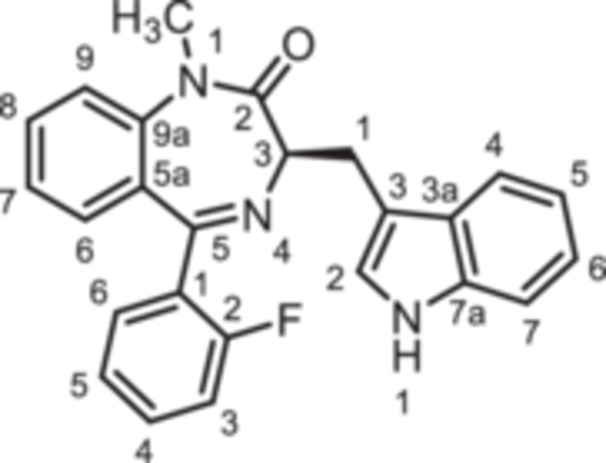


(3*R*)−5‐(2‐Fluorophenyl)−3‐[(1*H*‐indol‐3‐yl)methyl]−1‐methyl‐1,3‐dihydro‐1,4‐benzodiazepin‐2‐one (**9b**): Product **9b** was prepared according to the General Procedure B. 1,4‐Benzodiazepine **8b** (100 mg, 0.26 mmol, 1.00 eq.) was deprotonated with NaH (60% dispersion in paraffine, 11.0 mg, 0.27 mmol, 1.05 eq.) and subsequently treated with CH_3_I (16 µL, 37.2 mg, 0.27 mmol, 1.05 eq.). Colorless solid, mp 115°C, yield 80 mg (0.20 mmol, 78%). Purity (HPLC): 100.0% (t_R_ = 21.0 min). Chemical formula: C_25_H_20_FN_3_O (397.5 g/mol). TLC: R_f_ = 0.33 (cyclohexane/ethyl acetate 60:40). ^1^H NMR (600 MHz, DMSO‐d_6_): δ (ppm) = 3.38 (s, 3H, C*H*_3_), 3.46 (dd, *J* = 14.5/7.7 Hz, 1H, C*H*_2_), 3.58 (dd, *J* = 14.5/5.8 Hz, 1H, C*H*_2_), 3.71 (dd, *J* = 7.7/5.8 Hz, 1H, 3‐*H*_bdz_), 6.93 (ddd, *J* = 7.9/7.0/1.1 Hz, 1H, 5‐*H*_indole_), 7.03 (ddd, *J* = 8.1/7.0/1.2 Hz, 1H, 6‐*H*_indole_), 7.10 (dd, *J* = 7.9/1.6 Hz, 1H, 9‐*H*_bdz_), 7.13 (d, *J* = 2.3 Hz, 1H, 2‐*H*_indole_), 7.17 (ddd, *J* = 8.2/7.0/1.3 Hz, 1H, 7‐*H*_bdz_), 7.21 (ddd, *J* = 10.8/8.2/1.0 Hz, 1H, 3‐*H*_FPhe_), 7.27–7.33 (m, 2H, 4‐*H*_FPhe_, 7‐*H*_indole_), 7.48–7.61 (m, 5H, 5‐*H*_FPhe_, 6‐*H*_FPhe_, 6‐*H*_bdz_, 8‐*H*_bdz_, 4‐*H*_indole_), 10.80 (s, 1H, N*H*). ^13^C NMR (151 MHz, DMSO‐d_6_): δ (ppm) = 27.1 (C*H*_2_), 34.8 (*C*H_3_), 64.4 (C‐3_bdz_), 111.2 (C‐7_indole_), 111.2 (C‐3_indole_), 115.9 (d, *J* = 21.5 Hz, (C‐3_FPhe_), 118.1 (C‐5_indole_), 118.5 (C‐4_indole_), 120.8 (C‐6_indole_), 121.9 (C‐8_bdz_), 123.6 (C‐2_indole_), 124.3 (C‐7_bdz_), 124.5 (d, *J* = 3.3 Hz, C‐5_FPhe_), 126.8 (d, *J* = 12.6 Hz, C‐1_FPhe_), 127.4 (C‐3a_indole_), 128.2 (C‐9_bdz_), 129.4 (C‐5a_bdz_), 131.4 (d, *J* = 2.6 Hz, C‐6_FPhe_), 131.7 (C‐6_bdz_), 132.3 (d, *J* = 8.3 Hz, C‐4_FPhe_), 135.9 (C‐7a_indole_), 142.1 (C‐9a_bdz_), 159.8 (d, *J* = 249.5 Hz, C‐2_FPhe_), 164.2 (C‐5_bdz_), 169.2 (C=O). IR (neat): ν̃ (cm^−1^) = 3059 (C‐H, aryl), 1663 (C=O, amide), 1605 (C=C, aryl), 1331 (C‐N, amide). Exact mass (APCI): *m/z* = 398.1673 (calcd 398.1663 for C_25_H_21_FN_3_O [M+H]^+^). Specific rotation: ${\left[\alpha \right]}_{20}^{\text{D}}$ = +28.3 (c = 0.22; CH_3_OH).


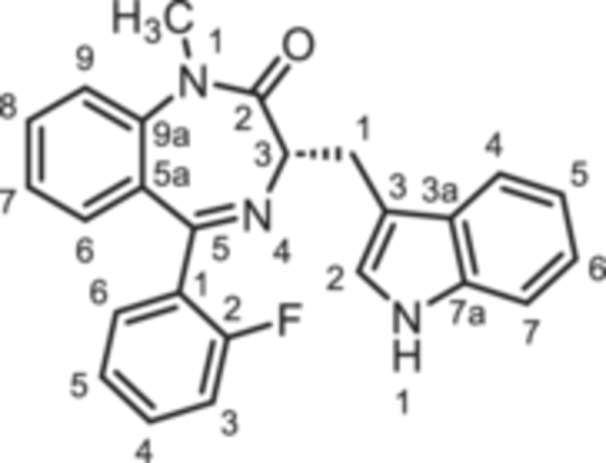


(3*S*)−5‐(2‐Fluorophenyl)−3‐[(1*H*‐indol‐3‐yl)methyl]−1‐methyl‐1,3‐dihydro‐1,4‐benzodiazepin‐2‐one (*ent‐* **9b**): Product *ent**‐*** **9b** was prepared according to the General Procedure B. 1,4‐Benzodiazepine *ent‐* **8b** (100 mg, 0.26 mmol, 1.00 eq.) was deprotonated with NaH (60% dispersion in paraffine, 11.0 mg, 0.27 mmol, 1.05 eq.) and subsequently treated with CH_3_I (16 µL, 37.2 mg, 0.27 mmol, 1.05 eq.). Colorless solid, mp 115°C, yield 65 mg (0.16 mmol, 63%). Purity (HPLC): 100.0% (t_R_ = 20.5 min). Chemical formula: C_25_H_20_FN_3_O (397.5 g/mol). TLC: R_f_ = 0.34 (cyclohexane/ethyl acetate 60:40). ^1^H NMR, ^13^C NMR, and IR sepctra are identical with the spectra of enantiomer **9b**. Exact mass (APCI): *m/z* = 398.1677 (calcd 398.1663 for C_25_H_21_FN_3_O [M+H]^+^). Specific rotation: ${\left[\alpha \right]}_{20}^{\text{D}}$ = −30.3 (c = 0.12; CH_3_OH).


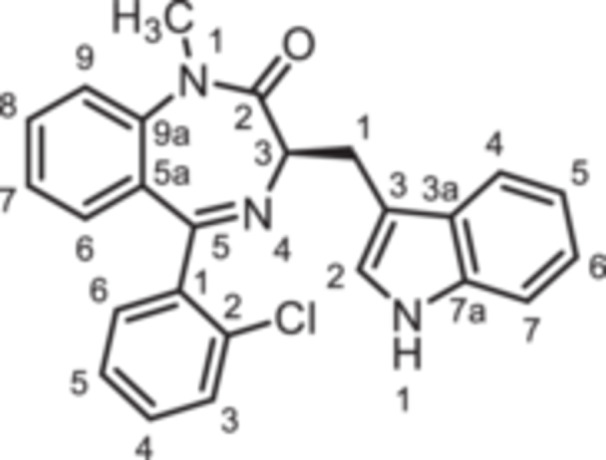


(3*R*)−5‐(2‐Chlorphenyl)−3‐[(1*H*‐indol‐3‐yl)methyl]−1‐methyl‐1,3‐dihydro‐1,4‐benzodiazepin‐2‐one (**9c**): Product **9c** was prepared according to the General Procedure B. 1,4‐Benzodiazepine **8c** (100 mg, 0.25 mmol, 1.00 eq.) was deprotonated with NaH (60% dispersion in paraffine, 10.5 mg, 0.26 mmol, 1.05 eq.) and subsequently treated with CH_3_I (16 µL, 36.6 mg, 0.26 mmol, 1.05 eq.). Colorless solid, mp 185°C−186°C, yield 54 mg (0.13 mmol, 52%). Purity (HPLC): 98.4% (t_R_ = 21.3 min). Chemical formula: C_25_H_20_ClN_3_O (413.9 g/mol). TLC: R_f_ = 0.28 (cyclohexane/ethyl acetate 60:40). ^1^H NMR (600 MHz, DMSO‐d_6_): δ (ppm) = 3.38 (s, 3H, C*H*_3_), 3.42 (dd, *J* = 14.6/7.4 Hz, 1H, C*H*_2_), 3.58 (dd, *J* = 14.6/6.2 Hz, 1H, C*H*_2_), 3.75 (dd, *J* = 7.4/6.2 Hz, 1H, 3‐*H*_bdz_), 6.92 (ddd, *J* = 7.9/6.9/1.0 Hz, 1H, 5‐*H*_indole_), 6.95 (dd, *J* = 7.9/1.5 Hz, 1H, 9‐*H*_bdz_), 7.03 (ddd, *J* = 8.1/6.9/1.1 Hz, 1H, 6‐*H*_indole_), 7.12 (d, *J* = 2.4 Hz, 1H, 2‐*H*_indole_), 7.14 (ddd, *J* = 8.1/6.8/1.5 Hz, 1H, 7‐*H*_bdz_), 7.31 (dd, *J* = 7.9/1.0 Hz, 1H, 7‐*H*_indole_), 7.52–7.61 (m, 7H, 6‐*H*_bdz_, 8‐*H*_bdz_, 3‐*H*_ClPhe_, 4‐*H*_ClPhe_, 5‐*H*_ClPhe_, 6‐*H*_ClPhe_, 4‐*H*_indole_), 10.79 (s, N*H*). ^13^C NMR (151 MHz, DMSO‐d_6_): δ (ppm) = 27.1 (C*H*_2_), 34.7 (*C*H_3_), 64.3 (C‐3_bdz_), 111.2 (C‐7_indole_), 111.3 (C‐3_indole_), 118.1 (C‐5_indole_), 118.6 (C‐4_indole_), 120.8 (C‐6_indole_), 122.1 (C‐8_bdz_), 123.7 (C‐2_indole_), 124.3 (C‐7_bdz_), 127.2 (C‐5_ClPhe_), 127.4 (C‐3a_indole_), 127.8 (C‐9_bdz_), 129.4 (C‐5a_bdz_), 129.7 (C‐4_ClPhe_), 131.1 (C‐6_ClPhe_), 131.2 (C‐3_ClPhe_), 131.7 (C‐6_bdz_), 131.8 (C‐2_ClPhe_), 136.0 (C‐7a_indole_), 138.2 (C‐1_ClPhe_), 142.5 (C‐9a_bdz_), 167.0 (C‐5_bdz_), 169.0 (C=O). IR (neat): ν̃ (cm^−1^) = 3055 (C–H, aryl), 1662 (C=O, amide), 1601 (C=C, aryl), 1335 (C–N, amide). Exact mass (APCI): *m/z* = 414.1364 (calcd 414.1368 for C_25_H_21_ClN_3_O [M+H]^+^). Specific rotation: ${\left[\alpha \right]}_{20}^{\text{D}}$ = +83.1 (c = 0.10; CH_3_OH).


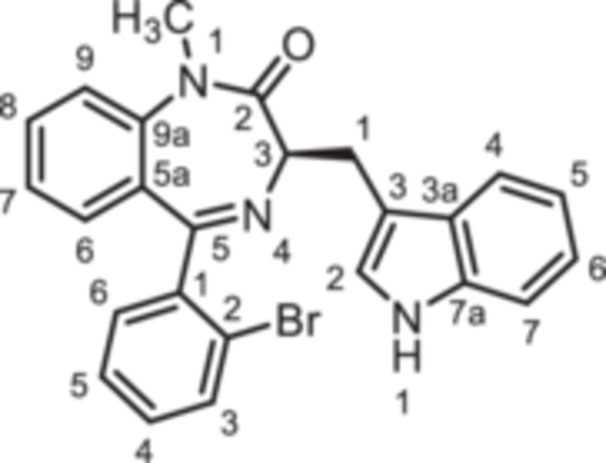


(3*R*)−5‐(2‐Bromophenyl)−3‐[(1*H*‐indol‐3‐yl)methyl]−1‐methyl‐1,3‐dihydro‐1,4‐benzodiazepin‐2‐one (**9d**): Product **9d** was prepared according to the General Procedure B. 1,4‐Benzodiazepine **8d** (100 mg, 0.23 mmol, 1.00 eq.) was deprotonated with NaH (60% dispersion in paraffine, 9.5 mg, 0.24 mmol, 1.05 eq.) and subsequently treated with CH_3_I (14 µL, 32.9 mg, 0.23 mmol, 1.00 eq.). Colorless solid, mp 240°C, yield 43 mg (0.09 mmol, 42%). Purity (HPLC): 98.8% (t_R_ = 21.6 min). Chemical formula: C_25_H_20_BrN_3_O (458.4 g/mol). TLC: R_f_ = 0.27 (cyclohexane/ethyl acetate 60:40). ^1^H NMR (600 MHz, DMSO‐d_6_): δ (ppm) = 3.39 (s, 3H, C*H*_3_), 3.42 (dd, *J* = 14.5/7.7 Hz, 1H, C*H*_2_), 3,59 (dd, *J* = 14.5/6.4 Hz, C*H*_2_), 3.75 (dd, *J* = 7.7/6.4 Hz, 1H, 3‐*H*_bdz_), 6.89–6.96 (m, 2H, 9‐*H*_bdz_, 5‐*H*_indole_), 7.03 (ddd, *J* = 8.1/7.0/1.1 Hz, 1H, 6‐*H*_indole_), 7.10–7.16 (m, 2H, 7‐*H*_bdz_, 2‐*H*_indole_), 7.31 (d, *J* = 8.2, 7‐*H*_indole_), 7.41 (td, *J* = 7.5/1.6 Hz, 1H, 4‐*H*_BrPhe_), 7.45 (dd, *J* = 7.5/1.8 Hz, 1H, 6‐*H*_BrPhe_), 7.48–7.60 (m, 4H, 5‐*H*_BrPhe_, 6‐*H*_bdz_, 8‐*H*_bdz,_ 4‐*H*_indole_), 7.63 (dd, *J* = 8.0/1.1 Hz, 1H, 3‐*H*_BrPhe_), 10.79 (s, 1H, N*H*). ^13^C NMR (151 MHz, DMSO‐d_6_): δ (ppm) = 27.1 (C*H*_2_), 34.6 (*C*H_3_), 64.3 (C‐3_bdz_), 111.2 (C‐7_indole_, 111.3 (C‐3_indole_), 118.1 (C‐5_indole_), 118.6 (C‐4_indole_), 120.8 (C‐6_indole_), 121.5 (C‐2_BrPhe_), 122.1 (C‐8_bdz_), 123.7 (C‐2_indole_), 124.2 (C‐7_bdz_), 127.4 (C‐5_BrPhe_), 127.7 (C‐3a_indole_), 127.8 (C‐9_bdz_), 129.2 (C‐5a_bdz_), 131.2 (C‐4_BrPhe_), 131.3 (C‐6_BrPhe_), 131.7 (C‐6_bdz_), 132.8 (C‐3_BrPhe_), 136.0 (C‐7a_indole_), 140.1 (C‐1_BrPhe_), 142.9 (C‐9a_bdz_), 168.1 (C‐5_bdz_), 168.9 (C=O). IR (neat): ν̃ (cm^−1^) = 3055 (C–H, aryl), 1663 (C=O, amide), 1601 (C=C, aryl), 1335 (C–N, amide). Exact mass (APCI): *m/z* = 458.0884 (calcd 458.0863 for C_25_H_21_^79^BrN_3_O [M+H]^+^). Specific rotation: ${\left[\alpha \right]}_{20}^{\text{D}}$ = +56.5 (c = 0.17; CH_3_OH).


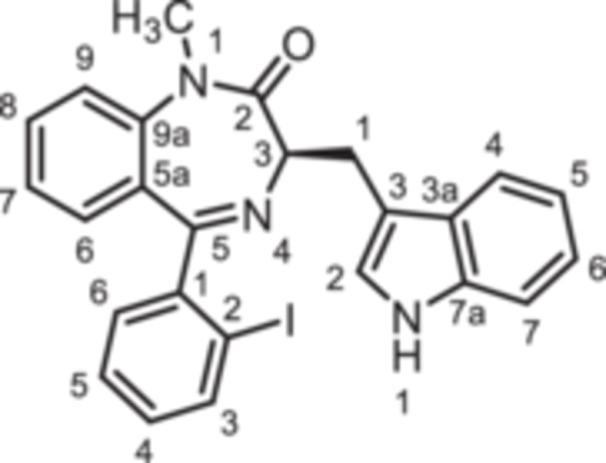


(3*R*)−3‐[(1*H*‐Indol‐3‐yl)methyl]−5‐(2‐iodphenyl)−1‐methyl‐1,3‐dihydro‐1,4‐benzodiazepin‐2‐one (**9e**): Product **9e** was prepared according to the General Procedure B. 1,4‐Benzodiazepine **8e** (100 mg, 0.20 mmol, 1.00 eq.) was deprotonated with NaH (60% dispersion in paraffine, 8.6 mg, 0.21 mmol, 1.05 eq.) and subsequently treated with CH_3_I (13 µL, 29.8 mg, 0.21 mmol, 1.05 eq.). Colorless solid, mp 228°C, yield 92 mg (0.18 mmol, 89%). Purity (HPLC): 98.1% (t_R_ = 21.7 min). Chemical formula: C_25_H_20_IN_3_O (505.4 g/mol). TLC: R_f_ = 0.26 (cyclohexane/ethyl acetate 70:30). ^1^H NMR (600 MHz, DMSO‐d_6_): δ (ppm) = 3.39 (dd, *J* = 14.5/6.9 Hz, 1H, C*H*_2_), 3.43 (s, 1H, C*H*_3_), 3.60 (dd, *J* = 14.5/6.5 Hz, C*H*_2_), 3.73 (dd, *J* = 6.9/6.5 Hz, 1H, 3‐*H*_bdz_), 6.87–6.95 (m, 2H, 9‐*H*_bdz_, 5‐*H*_indole_), 7.03 (ddd, *J* = 8.1/7.0/1.2 Hz, 1H, 6‐*H*_indole_), 7.09–7.16 (m, 2H, 2‐*H*_indole_, 7‐*H*_bdz_), 7.21 (td, *J* = 7.6/1.7 Hz, 1H, 4‐*H*_IPhe_), 7.31 (dt, *J* = 8.1/0.9 Hz, 1H, 7‐*H*_indole_), 7.39 (dd, *J* = 7.6/1.7 Hz, 1H, 6‐*H*_IPhe_), 7.48 – 7.57 (m, 4H, 6‐*H*_bdz_, 8‐*H*_bdz_, 5‐*H*_IPhe_, 4‐*H*_indole_), 7.86 (dd, *J* = 7.9/1.2 Hz, 1H, 3‐*H*_IPhe_), 10.79 (s, 1H, N*H*). ^13^C NMR (151 MHz, DMSO‐d_6_): δ (ppm) = 27.1 (C*H*_2_), 34.6 (*C*H_3_), 64.3 (C‐3_bdz_), 97.0 (C‐2_IPhe_), 111.2 (C‐7_indole_), 111.3 (C‐3_indole_), 118.1 (C‐5_indole_), 118.6 (C‐4_indole_), 120.8 (C‐6_indole_), 122.0 (C‐8_bdz_), 123.7 (C‐2_indole_), 124.2 (C‐7_bdz_), 127.4 (C‐3a_indole_), 128.1 (2 C, C‐9_bdz,_ C‐5_IPhe_), 128.9 (C‐5a_bdz_), 130.7 (C‐6_IPhe_), 130.8 (C‐4_IPhe_), 131.7 (C‐6_bdz_), 136.0 (C‐7a_indole_), 139.1 (C‐3_IPhe_), 143.6 (C‐1_IPhe_), 143.7 (C‐9a_bdz_), 168.8 (C‐5_bdz_), 170.1 (C=O). IR (neat): ν̃ (cm^−1^) = 3059 (C‐H, aryl), 1674 (C=O, amide), 1609 (C=C, aryl), 1335 (C‐N, amide). Exact mass (APCI): *m/z* = 506.0719 (calcd 506.0724 for C_25_H_21_IN_3_O [M+H]^+^). Specific rotation: ${\left[\alpha \right]}_{20}^{\text{D}}$ = +22.6 (c = 0.14; CH_3_OH).


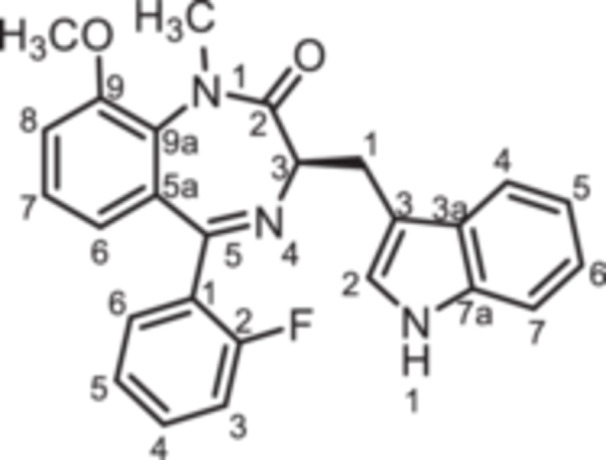


(3*R*)−5‐(2‐Fluorphenyl)−3‐[(1*H*‐indol‐3‐yl)methyl]−9‐methoxy‐1‐methyl‐1,3‐dihydro‐1,4‐benzodiazepin‐2‐one (**9h**): Product **9h** was prepared according to the General Procedure B. 1,4‐Benzodiazepine **8 h** (200 mg, 0.48 mmol, 1.00 eq.) was deprotonated with NaH (60% dispersion in paraffine, 20.3 mg, 0.51 mmol, 1.05 eq.) and subsequently treated with CH_3_I (31 µL, 70.8 mg, 0.50 mmol, 1.03 eq.). Colorless solid, mp 137°C–138°C, yield 120 mg (0.28 mmol, 58%). Purity (HPLC): 99.6% (t_R_ = 21.2 min). Chemical formula: C_26_H_22_FN_3_O (427.5 g/mol). TLC: R_f_ = 0.49 (cyclohexane/ethyl acetate 50:50). ^1^H NMR (600 MHz, DMSO‐d_6_): δ (ppm) = 3.14 (s, 3H, NC*H*_3_), 3.42 (dd, *J* = 14.4/7.7 Hz, 1H, C*H*_2_), 3.59 (dd, *J* = 14.4/6.4 Hz, 1H, C*H*_2_), 3.72 (dd, *J* = 7.7/6.4 Hz, 1H 3‐*H*_bdz_), 3.89 (s, 3H, OC*H*_3_), 6.66 (dd, *J* = 7.6/1.5 Hz, 1H, 8‐*H*_bdz_), 6.93 (ddd, *J* = 7.9/6.9/1.0 Hz, 1H, 5‐*H*_indole_), 7.03 (ddd, *J* = 8.1/7.0/1.2 Hz, 1H, 6‐*H*_indole_), 7.11 (d, *J* = 2.3 Hz, 1H, 2‐*H*_indole_), 7.19–7.27 (m, 3H, 6‐*H*_bdz_, 7‐*H*_bdz_, 3‐*H*_FPhe_), 7.29–7.33 (m, 2H, 5‐*H*_FPhe_, 7‐*H*_indole_), 7.52–7.59 (m, 3H, 4‐*H*_FPhe_, 6‐*H*_FPhe_, 4‐*H*_indole_), 10.80 (s, 1H, N*H*). ^13^C NMR (151 MHz, DMSO‐d_6_): δ (ppm) = 26.8 (C*H*_2_), 35.4 (N*C*H_3_), 56.0 (OCH_3_), 64.6 (C‐3_bdz_), 111.2 (C‐3_indole_), 111.3 (C‐7_indole_), 114.4 (C‐6_bdz_), 116.0 (d, *J* = 21.4 Hz, C‐3_FPhe_), 118.1 (C‐5_indole_), 118.6 (C‐4_indole_), 119.0 (C‐8_bdz_), 120.8 (C‐6_indole_), 123.7 (C‐2_indole_), 124.6 (d, *J* = 3.3 Hz, C‐5_FPhe_), 126.6 (C‐7_bdz_), 126.8 (d, *J* = 12.1 Hz, C‐1_FPhe_), 127.4 (C‐3a_indole_), 130.5 (C‐9a_bdz_), 131.2 (d, *J* = 2.3 Hz, C‐6_FPhe_), 131.6 (C‐5a_bdz_), 132.4 (d, *J* = 8.2 Hz, C‐4_FPhe_), 135.9 (C‐7a_indole_), 152.1 (C‐9_bdz_), 159.8 (d, *J* = 249.2 Hz, C‐2_FPhe_), 163.7 (C‐5_bdz_), 169.2 (C=O). IR (neat): ν̃ (cm^−1^) = 3063 (C–H, aryl), 1659 (C=O, amide), 1601 (C=C, aryl), 1323 (C–N, amide). Exact mass (APCI): *m/z* = 428.1783 (calcd 428.1769 for C_26_H_23_FN_3_O [M+H]^+^). Specific rotation: ${\left[\alpha \right]}_{20}^{\text{D}}$ = +55.0 (c = 0.33; CH_3_OH).


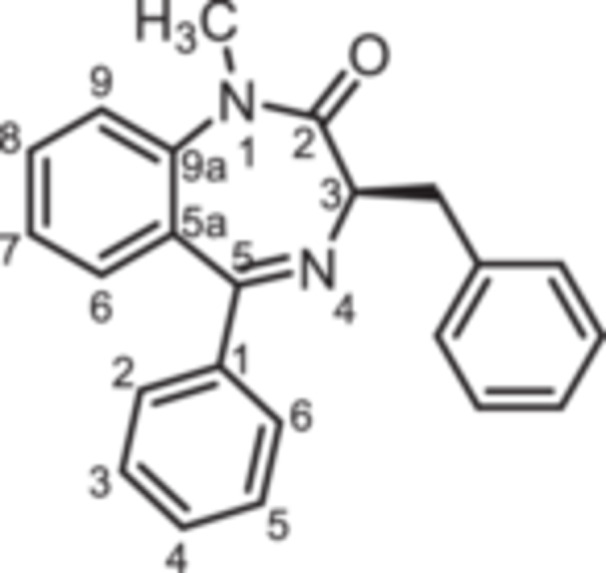


(3*R*)−3‐Benzyl‐1‐methyl‐5‐phenyl‐1,3‐dihydro‐1,4‐benzodiazepin‐2‐one (**13a**): Product **13a** was prepared according to the General Procedure B. 1,4‐Benzodiazepine **12a** (326 mg, 1.00 mmol, 1.00 eq.) was deprotonated with NaH (60% dispersion in paraffine, 42.0 mg, 1.05 mmol, 1.05 eq.) and subsequently treated with CH_3_I (64 µL, 146.2 mg, 1.03 mmol, 1.03 eq.). Colorless solid, mp 199°C–200°C, yield 249 mg (0.73 mmol, 73%). Purity (HPLC): 98.8% (t_R_ = 20.4 min). Chemical formula: C_23_H_20_N_2_O (340.4 g/mol). TLC: R_f_ = 0.27 (cyclohexane/ethyl acetate 80:20). ^1^H NMR (600 MHz, DMSO‐d_6_): δ (ppm) = 3.35 (s, 3H, C*H*_3_), 3.36–3.44 (m, 2H, CH_2_), 3.71 (dd, *J* = 8.0/5.7 Hz, 1H, 3‐*H*_bdz_), 7.14–7.18 (m, 1H, 4‐*H*_benzyl_), 7.20–7.27 (m, 4H, 6‐*H*_bdz_, 7‐*H*_bdz_, 3‐*H*_benzyl_, 5‐*H*_benzyl_), 7.28–7.33 (m, 2H, 2‐*H*_benzyl_, 6‐*H*_benzyl_), 7.41–7.45 (m, 2H, 2‐*H*_Phe_, 6‐*H*_Phe_), 7.47–7.51 (m, 3H, 3‐*H*_Phe,_ 4‐*H*_Phe_ 5‐H_Phe_), 7.58 (dd, *J* = 8.4/1.1 Hz, 1H, 9‐*H*_bdz_), 7.64 (ddd, *J* = 8.6/7.0/1.8 Hz, 1H, 8‐*H*_bdz_). ^13^C NMR (151 MHz, DMSO‐d_6_): δ (ppm) = 34.7 (*C*H_3_), 37.6 (C*H*_2_), 64.7 (C‐3_bdz_), 121.9 (C‐9_bdz_), 124.0 (C‐7_bdz_), 126.0 (C‐4_benzyl_), 128.0 (2C, C‐3_benzyl_, C‐5_benzyl_), 128.1 (C‐5a_bdz_), 128.3 (2C, C‐2_Phe_, C‐6_Phe_), 129.2 (2C, C‐3_Phe_, C‐5_Phe_), 129.5 (C‐6_bdz_), 129.6 (2 C, C‐2_benzyl_, C‐6_benzyl_), 130.4 (C‐4_Phe_), 131.7 (C‐8_bdz_), 138.2 (C‐1_Phe_), 139.2 (C‐1_benzyl_), 143.2 (C‐9a_bdz_), 167.3 (C‐5_bdz_), 169.5 (C=O). IR (neat): ν̃ (cm^−1^) = 3055 (C–H, aryl), 1681 (C=O, amide), 1601 (C=C, aryl), 1099 (C–N, amide). Exact mass (APCI): *m/z* = 341.1649 (calcd 341.1648 for C_23_H_21_N_2_O [M+H]^+^). Specific rotation: ${\left[\alpha \right]}_{20}^{\text{D}}$ = −38.4 (c = 0.12; CH_3_OH).


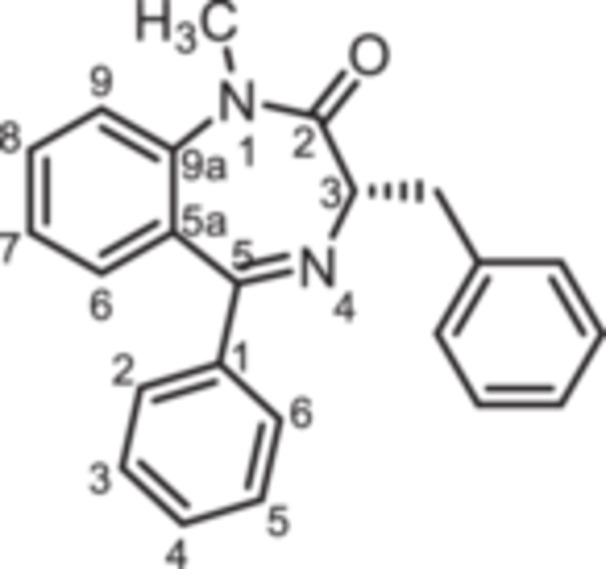


(3*S*)−3‐Benzyl‐1‐methyl‐5‐phenyl‐1,3‐dihydro‐1,4‐benzodiazepin‐2‐one (*ent‐* **13a**): Product *ent‐* **13a** was prepared according to the General Procedure B. 1,4‐Benzodiazepine *ent‐* **12a** (326.4 mg, 1.00 mmol, 1.00 eq.) was deprotonated with NaH (60% dispersion in paraffine, 42.0 mg, 1.05 mmol, 1.05 eq.) and subsequently treated with CH_3_I (64 µL, 146.2 mg, 1.03 mmol, 1.03 eq.). Colorless solid, mp 199°C–200°C, yield 276 mg (0.81 mmol, 81%). Purity (HPLC): 100.0% (t_R_ = 20.4 min). Chemical formula: C_23_H_20_N_2_O (340.4 g/mol). TLC: R_f_ = 0.27 (cyclohexane/ethyl acetate 80:20). ^1^H NMR, ^13^C NMR, and IR sepctra are identical with the spectra of enantiomer **12a**. Exact mass (APCI): *m/z* = 341.1653 (calcd 341.1648 for C_23_H_21_N_2_O [M+H]^+^). Specific rotation: ${\left[\alpha \right]}_{20}^{\text{D}}$ = +45.3 (c = 0.09; CH_3_OH).


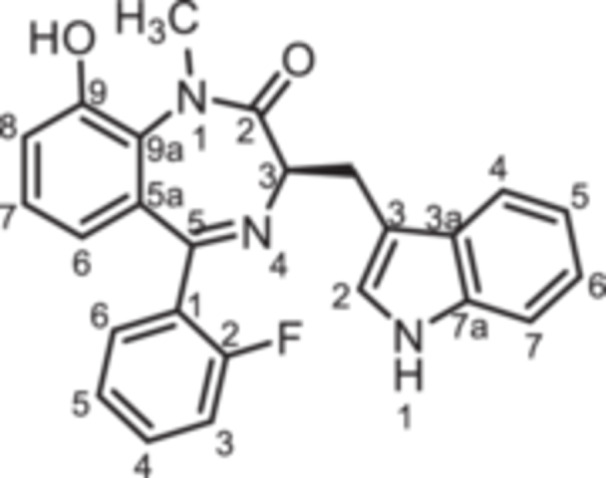


(3*R*)−5‐(2‐Fluorphenyl)−9‐hydroxy‐3‐[(1*H*‐indol‐3‐yl)methyl]−1,3‐dihydro‐1‐methyl‐1,4‐benzodiazepin‐2‐one (**9i**): At 0°C, a solution of BBr_3_ in CH_2_Cl_2_ (1 M, 0.60 mL, 0.60 mmol, 3.16 eq.) was added to a solution of **8h** (80 mg, 0.19 mmol, 1.00 eq.) in CH_2_Cl_2_ (1.00 mL). The ice bath was removed and the mixture was stirred at room temperature for 3 h. Then, CH_3_OH (3.00 mL) was added and the mixture was stirred for 1 h. CH_2_Cl_2_ (30 mL) and water (30 mL) were added and the aqueous layer was extracted with CH_2_Cl_2_ (3 × 30 mL). The organic layer was dried (Na_2_SO_4_), filtered and concentrated in vacuo. The residue was purified by flash chromatography (SiO_2_, cyclohexane/ethyl acetate, gradient 100:0 → 0:100). Colorless solid, mp 221°C, yield 43 mg (0.10 mmol, 55%). Purity (HPLC): 96.8% (t_R_ = 19.0 min). Chemical formula: C_25_H_20_FN_3_O_2_ (413.5 g/mol). TLC: R_f_ = 0.31 (cyclohexane/ethyl acetate 50:50). ^1^H NMR (600 MHz, DMSO‐d_6_): δ (ppm) = 3.17 (s, 3H, C*H*_3_), 3.41 (dd, *J* = 14.5/7.2 Hz, 1H, C*H*_2_), 3.58 (dd, *J* = 14.5/6.4 Hz, 1H, C*H*_2_), 3.74 (dd, *J* = 7.2/6.4 Hz, 1H, 3‐*H*_bdz_), 6.52 (dd, *J* = 5.3/3.8 Hz, 1H, 8‐*H*_bdz_), 6.93 (td, *J* = 7.0/1.0 Hz, 1H, 5‐*H*_indole_), 7.01–7.08 (m, 3H, 6‐*H*_indole_, 6‐*H*_bdz_, 7‐*H*_bdz_), 7.11 (d, *J* = 2.3 Hz, 1H, 2‐*H*_indole_), 7.22 (ddd, *J* = 9.5/8.3/1.1 Hz, 1H, 3‐*H*_FPhe_), 7.27–7.33 (m, 2H, 5‐*H*_FPhe_, 7‐*H*_indole_), 7.49–7.58 (m, 3H, 4‐*H*_FPhe_, 6‐*H*_FPhe_, 4‐*H*_indole_), 10.41 (s, 1H, OH), 10.80 (s, 1H, N*H*). ^13^C NMR (151 MHz, DMSO‐d_6_): δ (ppm) = 26.9 (C*H*_2_), 34.8 (*C*H_3_), 64.4 (C‐3_bdz_), 111.3 (C‐3_indole_), 111.3 (C‐7_indole_), 116.0 (d, *J* = 21.3 Hz, C‐3_FPhe_), 117.8 (C‐8_bdz_), 118.1 (C‐5_indole_), 118.5 (C‐6_bdz_), 118.6 (C‐4_indole_), 120.8 (C‐6_indole_), 123.6 (C‐2_indole_), 124.5 (d, *J* = 3.4 Hz, C‐5_FPhe_), 126.4 (C‐7_bdz_), 127.0 (d, *J* = 12.4 Hz, C‐1_FPhe_), 127.4 (C‐3a_indole_), 129.5 (C‐5a_bdz_), 131.2 (d, *J* = 2.5 Hz, C‐6_FPhe_), 131.9 (C‐9a_bdz_), 132.2 (d, *J* = 8.3 Hz, C‐4_FPhe_), 135.9 (C‐7a_indole_), 150.5 (C‐9_bdz_), 159.8 (d, *J* = 249.1 Hz, C‐2_FPhe_), 164.0 (C‐5_bdz_), 169.2 (C=O). IR (neat): ν̃ (cm^−1^) = 3124 (O–H, C–H, aryl), 1620 (C=O, amide), 1601 (C=C, aryl), 1254 (C–N, amide). Exact mass (APCI): *m/z* = 414.1609 (calcd 414.1612 for C_25_H_21_FN_3_O_2_ [M+H]^+^). Specific rotation: ${\left[\alpha \right]}_{20}^{\text{D}}$ = +85.5 (c = 0.09; CH_3_OH).


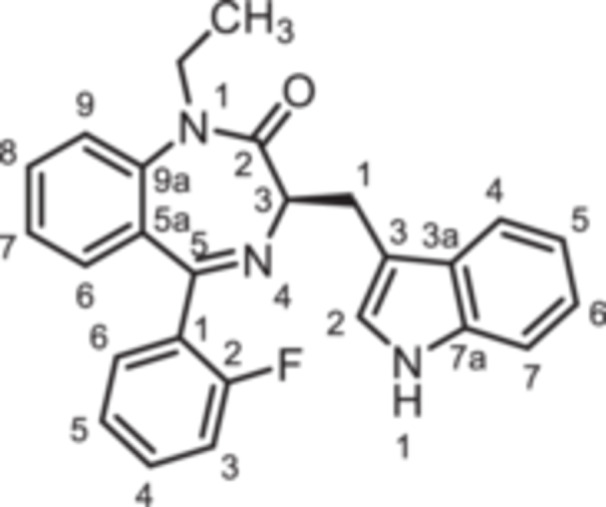


(3*R*)−1‐Ethyl‐5‐(2‐fluorphenyl)−3‐[(1*H*‐indol‐3‐yl)methyl]−1,3‐dihydro‐1,4‐benzodiazepin‐2‐one (**14**): At 0°C, 1,4‐benzodiazepine **8b** (92 mg, 0.50 mmol, 1.00 eq.) was deprotonated with NaH (60% dispersion in paraffine, 21.00 mg, 0.53 mmol, 1.05 eq.). The ice bath was removed and ethyl methanesulfonate (56 µL, 63.9 mg, 0.52 mmol, 1.03 eq.) was added. Stirring of the mixture was continued at 40°C for 1 h. The mixture was then poured into a saturated solution of NH_4_Cl (10 mL). CH_2_Cl_2_ (30 mL) and H_2_O (20 mL) were added and the product was extracted with CH_2_Cl_2_ (3 × 30 mL). The combined organic layers were dried (Na_2_SO_4_), filtered and concentrated in vacuo. The residue was purified by flash chromatography (SiO_2_, CH_2_Cl_2_/CH_3_OH, gradient 100:0 → 90:10). Colorless solid, mp 101°C, yield 169 mg (0.41 mmol, 82%). Purity (HPLC): 97.8% (t_R_ = 22.0 min). Chemical formula: C_26_H_22_FN_3_O (411.5 g/mol). TLC: R_f_ = 0.24 (cyclohexane/ethyl acetate 70:30). ^1^H NMR (600 MHz, DMSO‐d_6_): δ (ppm) = 0.94 (t, *J* = 7.0 Hz, 3H, CH_3_CH_2_N), 3.46 (dd, *J* = 14.5/7.2 Hz, 1H, C*H*_2_‐aryl), 3.59 (dd, *J* = 14.5/6.3 Hz, 1H, C*H*_2_‐aryl), 3.68–3.77 (m, 2H, 3‐*H*_bdz_, CH_3_C*H*_2_N), 4.31 (dq, *J* = 14.3/7.1 Hz, 1H, CH_3_C*H*_2_N), 6.93 (ddd, *J* = 8.0/6.9/1.0 Hz, 1H, 5‐*H*_indole_), 7.03 (ddd, *J* = 8.1/7.0/1.2 Hz, 1H, 6‐*H*_indole_), 7.09 (dd, *J* = 7.9/1.5 Hz, 1H, 9‐*H*_bdz_), 7.12 (d, *J* = 2.3 Hz, 1H, 2‐*H*_indole_), 7.15–7.25 (m, 2H, 7‐*H*_bdz_, 3‐*H*_FPhe_), 7.28–7.34 (m, 2H, 6‐*H*_FPhe_, 7‐*H*_indole_), 7.50–7.63 (m, 5H, 6‐*H*_bdz_, 8‐*H*_bdz_, 4‐*H*_FPhe_, 5‐*H*_FPhe_, 4‐*H*_indole_), 10.79 (s, 1H, N*H*). ^13^C NMR (151 MHz, DMSO‐d_6_): δ (ppm) = 12.7 (*C*H_3_C*H*_2_N), 27.0 (C*H*_2_‐aryl), 41.5 (CC*H*_2_CN), 64.6 (C‐3_bdz_), 111.2 (C‐7_indole_), 111.2 (C‐3_indole_), 116.0 (d, *J* = 21.3 Hz, C‐3_FPhe_), 118.1 (C‐5_indole_), 118.6 (C‐4_indole_), 120.8 (C‐6_indole_), 122.8 (C‐8_bdz_), 123.6 (C‐2_indole_), 124.6 (d, *J* = 3.1 Hz, C‐6_FPhe_), 124.8 (C‐7_bdz_), 126.8 (d, *J* = 11.9 Hz, C‐1_FPhe_), 127.4 (C‐3a_indole_), 128.0 (C‐9_bdz_), 131.0 (C‐5a_bdz_), 131.3 (d, *J* = 2.4 Hz, C‐5_FPhe_), 131.7 (C‐6_bdz_), 132.3 (d, *J* = 8.3 Hz, C‐4_FPhe_), 135.9 (C‐7a_indole_), 140.2 (C‐9a_bdz_), 159.9 (d, *J* = 249.2 Hz, C‐2_FPhe_), 164.1 (C‐5a_bdz_), 167.9 (C=O). IR (neat): ν̃ (cm^−1^) = 3059 (C‐H, aryl), 1662 (C=O, amide), 1601 (C=C, aryl), 1092 (C–N, amide). Exact mass (APCI): *m/z* = 412.1796 (calcd 412.1820 for C_26_H_23_FN_3_O [M+H]^+^). Specific rotation: ${\left[\alpha \right]}_{20}^{\text{D}}$ = +12.5 (c = 0.15; CH_3_OH).


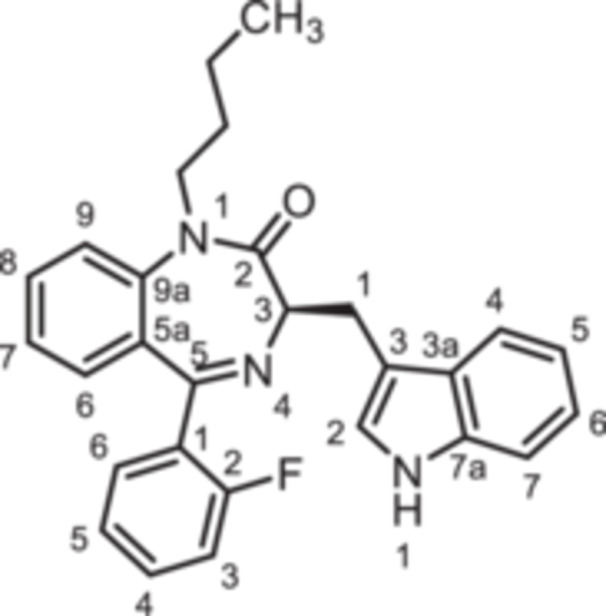


(3*R*)−1‐Butyl‐5‐(2‐fluorphenyl)−3‐[(1*H*‐indol‐3‐yl)methyl]−1,3‐dihydro‐1,4‐benzodiazepin‐2‐one (**15**): At 0°C, 1,4‐benzodiazepine **8b** (192 mg, 0.50 mmol, 1.00 eq.) was deprotonated with NaH (60% dispersion in paraffine, 21.0 mg, 0.53 mmol, 1.05 eq.). The ice bath was removed and butyl methanesulfonate (71 µL, 78.4 mg, 0.52 mmol, 1.03 eq.) was added. Stirring of the mixture was continued at 40°C for 1 h. The mixture was then poured into a saturated solution of NH_4_Cl (10 mL). CH_2_Cl_2_ (30 mL) and H_2_O (20 mL) were added and the product was extracted with CH_2_Cl_2_ (3 ×30 mL). The combined organic layers were dried (Na_2_SO_4_), filtered and concentrated in vacuo. The residue was purified by flash chromatography (SiO_2_, CH_2_Cl_2_/CH_3_OH, gradient 100:0 → 90:10). Colorless solid, mp 100°C–101°C, yield 119 mg (0.27 mmol, 54%). Purity (HPLC): 100.0% (t_R_ = 23.6 min). Chemical formula: C_28_H_26_FN_3_O (439.5 g/mol). TLC: R_f_ = 0.37 (cyclohexane/ethyl acetate 70:30). ^1^H NMR (600 MHz, DMSO‐d_6_): δ (ppm) = 0.74 (t, *J* = 7.3 Hz, 3H, CH_3_CH_2_CH_2_CH_2_N), 1.10 (sext, *J* = 7.1 Hz, 2H, CH_3_CH_2_CH_2_CH_2_N), 1.21–1.36 (m, 2H, CH_3_CH_2_CH_2_CH_2_N), 3.46 (dd, *J* = 14.5/7.6 Hz, 1H, C*H*_2_‐aryl), 3.58 (dd, *J* = 14.5/6.1 Hz, 1H, CH_2_‐aryl), 3.67–3.75 (m, 2H, CH_3_CH_2_CH_2_CH_2_N, 3‐H_bdz_), 4.35–4.42 (m, 1H, CH_3_CH_2_CH_2_CH_2_N), 6.93 (t, *J* = 7.4 Hz, 1H, 5‐*H*_indole_), 7.03 (t, *J* = 7.5 Hz, 1H, 6‐*H*_indole_), 7.08–7.14 (m, 2H, 2‐*H*_indole_, 9‐*H*_bdz_), 7.17 (t, *J* = 7.5 Hz, 1H, 7‐*H*_bdz_), 7.23 (dd, *J* = 10.8/8.2 Hz, 1H, 3‐*H*_FPhe_), 7.28–7.33 (m, 2H, 5‐*H*_FPhe_, 7‐*H*_indole_), 7.50–7.59 (m, 4H, 6‐*H*_bdz_, 4‐*H*_FPhe_, 6‐*H*_FPhe_, 4‐*H*_indole_), 7.63 (d, *J* = 8.3 Hz, 1H, 8‐*H*_bdz_), 10.79 (s, 1H, N*H*). ^13^C NMR (151 MHz, DMSO‐d_6_): δ (ppm) = 13.4 (CH_3_CH_2_CH_2_CH_2_N), 19.1 (CH_3_CH_2_CH_2_CH_2_N), 27.0 (CH_2_‐aryl), 29.3 (CH_3_CH_2_CH_2_CH_2_N), 45.3 (CH_3_CH_2_CH_2_CH_2_N), 64.6 (C‐3_bdz_), 111.2 (C‐7_indole_), 111.2 (C‐3_indole_), 116.0 (d, *J* = 21.5 Hz, C‐3_FPhe_), 118.1 (C‐5_indole_), 118.6 (C‐4_indole_), 120.8 (C‐6_indole_), 122.7 (C‐8_bdz_), 123.6 (C‐2_indole_), 124.5 (d, *J* = 3.2 Hz, C‐5_FPhe_), 124.7 (C‐7_bdz_), 126.6 (d, *J* = 12.0 Hz, C‐1_FPhe_), 127.4 (C‐3a_indole_), 128.1 (C‐9_bdz_), 130.9 (C‐5a_bdz_), 131.4 (d, *J* = 2.2 Hz, C‐6_FPhe_), 131.6 (C‐6_bdz_), 132.4 (d, *J* = 8.4 Hz, C‐4_FPhe_), 135.9 (C‐7a_indole_), 140.3 (C‐9a_bdz_), 159.9 (d, *J* = 249.8 Hz, C‐2_FPhe_), 164.0 (C‐5_bdz_), 168.5 (C=O). IR (neat): ν̃ (cm^−1^) = 3059 (C–H, aryl), 1659 (C=O, amide), 1601 (C=C, aryl), 1215 (C–N, amide). Exact mass (APCI): *m/z* = 440.2118 (calcd 440.2133 for C_28_H_27_FN_3_O [M+H]^+^). Specific rotation: ${\left[\alpha \right]}_{20}^{\text{D}}$ = +9.3 (c = 0.14; CH_3_OH).


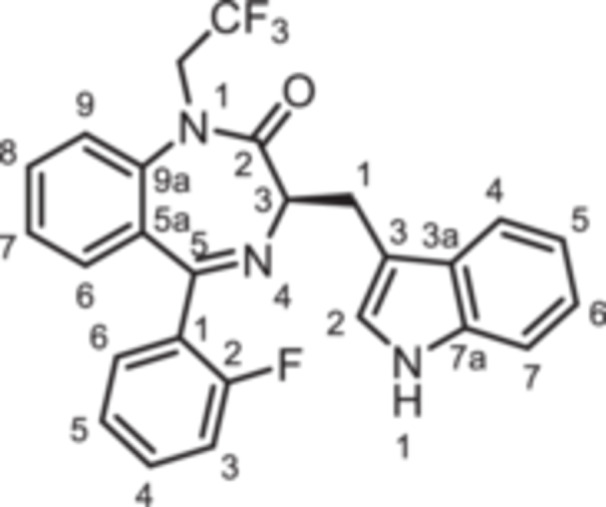


(3*R*)−5‐(2‐Fluorphenyl)−3‐[(1*H*‐indol‐3‐yl)methyl]−1‐(2,2,2‐trifluorethyl)−1,3‐dihydro‐1,4‐benzodiazepin‐2‐one (**16**): At 0°C, 1,4‐benzodiazepine **8b** (192 mg, 0.50 mmol, 1.00 eq.) was deprotonated with NaH (60% dispersion in paraffine, 21.0 mg, 0.53 mmol, 1.05 eq.). The ice bath was removed and 2,2,2‐trifluoroethyl trifluoromethanesulfonate (74 µL, 119.5 mg, 0.52 mmol, 1.03 eq.) was added. Stirring of the mixture was continued at room temperature for 40 min. The mixture was then poured into a saturated solution of NH_4_Cl (10 mL). CH_2_Cl_2_ (30 mL) and H_2_O (20 mL) were added and the product was extracted with CH_2_Cl_2_ (3 × 30 mL). The combined organic layers were dried (Na_2_SO_4_), filtered and concentrated in vacuo. The residue was purified by flash chromatography (SiO_2_, CH_2_Cl_2_/CH_3_OH, gradient 100:0 → 90:10). Colorless solid, mp 129°C–130°C, yield 212 mg (0.46 mmol, 91%). Purity (HPLC): 98.0% (t_R_ = 23.1 min). Chemical formula: C_26_H_19_F_4_N_3_O (465.5 g/mol). TLC: R_f_ = 0.30 (cyclohexane/ethyl acetate 70:30). ^1^H NMR (600 MHz, DMSO‐d_6_): δ (ppm) = 3.48 (dd, *J* = 14.4/7.6 Hz, 1H, CH_2_‐aryl), 3.57 3.57 (dd, *J* = 14.4/5.7 Hz, 1H, CH_2_‐aryl), 3.80–3.85 (dd, *J* = 7.6/5.7, Hz, 1H, 3‐*H*_bdz_), 4.71–4.81 (m, 1H, NCH_2_CF_3_), 5.13–5.23 (m, 1H, NCH_2_CF_3_), 6.93 (t, *J* = 7.4 Hz, 1H, 5‐*H*_indole_), 7.03 (t, *J* = 7.6 Hz, 1H, 6‐*H*_indole_), 7.10–7.15 (m, 2H, 2‐*H*_indole_, 9‐*H*_bdz_), 7.20–7.27 (m, 2H, 7‐*H*_bdz_, 3‐*H*_FPhe_), 7.27–7.33 (m, 2H, 5‐*H*_FPhe_, 7‐*H*_indole_), 7.49 (td, *J* = 7.6/1.7 Hz, 1H, 8‐*H*_bdz_), 7.52–7.58 (m, 2H, 4‐*H*_FPhe_, 4‐*H*_indole_), 7.60 (ddd, *J* = 8.7/7.3/1.6 Hz, 1H, 6‐*H*_bdz_), 7.78 (d, *J* = 8.4 Hz, 1H, 6‐*H*_FPhe_), 10.81 (s, 1H, N*H*). ^13^C NMR (151 MHz, DMSO‐d_6_): δ (ppm) = 26.9 (C*H*_2_‐aryl), 45.6 (q, *J* = 32.9 Hz, NC*H*_2_CF_3_), 63.9 (C‐3_bdz_), 110.8 (C‐7_indole_), 111.3 (C‐3_indole_), 116.1 (d, *J* = 21.2 Hz, C‐3_FPhe_), 118.1 (C‐5_indole_), 118.6 (C‐4_indole_), 120.8 (C‐6_indole_), 122.6 (C‐8_bdz_), 123.3 (q, *J* = 284 Hz NC*H*_2_ *C*F_3_), 123.7 (C‐2_indole_), 124.5 (d, *J* = 3.4 Hz, C‐5_FPhe_), 125.5 (C‐7_bdz_), 126.4 (d, *J* = 12.1 Hz, C‐1_FPhe_), 127.4 (C‐3a_indole_), 128.1 (C‐9_bdz_), 130.8 (C‐5a_bdz_), 131.3 (d, *J* = 2.4 Hz, C‐6_FPhe_), 131.8 (C‐6_bdz_), 132.5 (d, *J* = 8.2 Hz, C‐4_FPhe_), 135.9 (C‐7a_indole_), 139.6 (C‐9a_bdz_), 159.9 (d, *J* = 250.9 Hz, C‐2_FPhe_), 164.6 (C‐5_bdz_), 169.7 (C=O). IR (neat): ν̃ (cm^−1^) = 3059 (C–H, aryl), 1689 (C=O, amide), 1605 (C=C, aryl), 1327 (C–N, amide). Exact mass (APCI): *m/z* = 466.1536 (calcd 466.1537 for C_26_H_20_F_4_N_3_O [M+H]^+^). Specific rotation: ${\left[\alpha \right]}_{20}^{\text{D}}$ = +72.9 (c = 0.18; CH_3_OH).


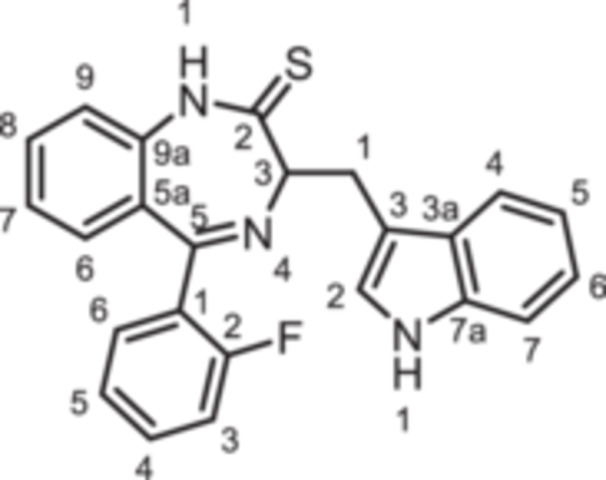


(*R*)−5‐(2‐Fluorophenyl)−3‐[(1*H*‐indol‐3‐yl)methyl]−1,3‐dihydro‐1,4‐benzodiazepin‐2‐thione ((*R*)‐**17**) and *(S)*−5‐(2‐fluorophenyl)−3‐[(1*H*‐indol‐3‐yl)methyl]−1,3‐dihydro‐1,4‐benzodiazepin‐2‐thione (*(S)*‐**17**): A solution of **8b** (200 mg, 0.52 mmol, 1.0 eq.) was stirred in THF (5 mL). Lawesson's Reagent (420 mg, 1.04 mmol, 1.0 eq.) was added and the mixture was heated to reflux over night. After cooling down to room temperature water (40 mL) was added and the aqueous layer was extracted with CH_2_Cl_2_ (3 × 40 mL). The organic layer was dried (Na_2_SO_4_), filtered and concentrated in vacuo. The residue was purified by flash chromatography (SiO_2_, cyclohexane/ethyl acetate, gradient 100:0 → 0:100). Slightly yellow solid, mp 200°C–201°C, yield 152 mg (0.38 mmol, 73%). Purity (HPLC): 98.3% (t_R_ = 22.5 min). Chemical formula: C_24_H_18_FN_3_S (399.5 g/mol). TLC: R_f_ = 0.30 (cyclohexane/ethyl acetate 70:30). ^1^H NMR (600 MHz, DMSO‐d_6_): δ (ppm) = 3.69 (d, *J* = 6.6 Hz, 2H, CH_2_), 3.87 (t, *J* = 6.6 Hz, 1H, 3‐*H*_bdz_), 6.90–6.96 (m, 1H, 5‐*H*_indole_), 7.04 (ddd, *J* = 8.2/6.9/1.2 Hz, 1H, 6‐*H*_indole_), 7.09 (d, *J* = 7.9 Hz, 1H, 9‐*H*_bdz_), 7.15–7.23 (m, 3H, 7‐*H*_bdz_, 3‐*H*_FPhe_, 2‐*H*_indole_), 7.26 (td, *J* = 7.6/1.1 Hz, 1H, 5‐*H*_FPhe_), 7.32 (d, *J* = 8.1 Hz, 1H, 7‐*H*_indole_), 7.34–7.39 (m, 2H, 6‐*H*_bdz_, 8‐*H*_bdz_), 7.50–7.58 (m, 3H, 4‐*H*_indole_, 4‐*H*_FPhe_, 6‐*H*_FPhe_), 10.81 (s, 1H, N*H*_indole_), 12.58 (s, 1H, N*H*_bdz_). ^13^C NMR (151 MHz, DMSO‐d_6_): δ (ppm) = 30.1 (C*H*_2_) 67.6 (C‐3_bdz_), 111.3 (C‐3_indole_), 111.3 (C‐7_indole_), 116.0 (d, *J* = 21.3 Hz, C‐3_FPhe_), 118.1 (C‐5_indole_), 118.5 (C‐4_indole_), 120.8 (C‐6_indole_), 121.6 (C‐8_bdz_), 123.8 (C‐7_bdz_), 124.5 (d, *J* = 3.2 Hz, C‐5_FPhe_), 124.9 (C‐2_indole_), 126.8 (d, *J* = 12.2 Hz, C‐1_FPhe_), 127.5 (C‐3a_indole_), 128.9 (C‐5a_bdz_), 129.2 (C‐9_bdz_), 131.6 (C‐6_bdz_), 131.7 (d, *J* = 3.4 Hz, C‐6_FPhe_), 132.4 (d, *J* = 8.4 Hz, C‐4_FPhe_), 136.0 (C‐7a_indole_), 138.2 (C‐9a_bdz_), 159.6 (d, *J* = 249.1 Hz, C‐2_FPhe_), 164.0 (C‐5_bdz_), 200.6 (C = S). IR (neat): ν̃ (cm^−1^) = 3059 (C‐H, aryl), 1605 (C = C, aryl), 1377 (C–N, thioamide), 1150 (C = S). Exact mass (APCI): *m/z* = 400.1270 (calcd 400.1278 for C_24_H_19_FN_3_S [M + H]^+^). 68 mg of the racemic mixture was separated by chiral HPLC (method 1, Supporting Information). *(S)*‐**17**: Colorless solid, yield 32 mg (0.08 mmol, 47%). Purity (HPLC method 1): 98.5%, t_R_ = 20.9 min. Specific rotation: ${\left[\alpha \right]}_{20}^{\text{D}}$ = −69.7 (c = 0.16; CH_3_OH). (*R*)*‐* **17:** Colorless solid, yield 29 mg (0.07 mmol, 43%). Purity (HPLC method 1): 97.8%, t_R_ = 20.9 min. Specific rotation: ${\left[\alpha \right]}_{20}^{\text{D}}$ = +63.9 (c = 0.15; CH_3_OH).


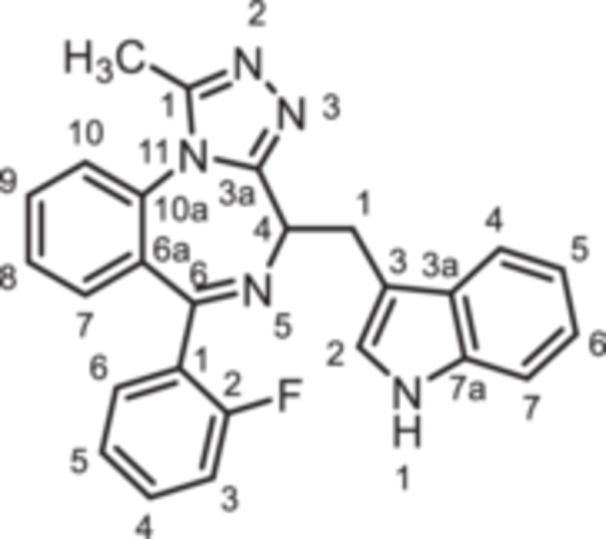


(*R*)*‐*6‐(2‐Fluorophenyl)−4‐[(1*H*‐indol‐3‐yl)methyl]−1‐methyl‐4*H*‐ [1,2,4]triazolo[4,3‐a]−1,4‐benzodiazepine ((*R*)‐**18**) and *(S)‐*6‐(2‐fluorophenyl)−4‐[(1*H*‐indol‐3‐yl)methyl]−1‐methyl‐4*H*‐ [1,2,4]triazolo[4,3‐a]−1,4‐benzodiazepine (*(S)*‐**18**): A solution of **18** (100 mg, 0.25 mmol, 1.0 eq.) was stirred in butan‐1‐ol (2.5 mL). Acetohydrazide (75 mg, 1.00 mmol, 4.0 eq.) was added and the mixture was heated to reflux over night. After cooling down to room temperature water (50 mL) was added and the aqueous layer was extracted with CH_2_Cl_2_ (3 × 50 mL). The organic layer was dried (Na_2_SO_4_), filtered and concentrated in vacuo. The residue was purified by flash chromatography (SiO_2_, CH_2_Cl_2_/MeOH, gradient 100:0 → 90:10). Colorless solid, mp 130°C–131°C, yield 37 mg (0.09 mmol, 35%). Purity (HPLC): 99.3% (t_R_ = 19.9 min). Chemical formula: C_26_H_20_FN_5_ (421.5 g/mol). TLC: R_f_ = 0.39 (CH_2_Cl_2_/methanol 95:5). ^1^H NMR (600 MHz, DMSO‐d_6_): δ (ppm) = 2.55 (s, 3H, C*H*_3_), 3.76 (dd, *J* = 14.5/8.3 Hz, 1H, C*H*_2_), 3.86 (dd, *J* = 14.5/5.9 Hz, 1H, C*H*_2_), 4.25 (dd, *J* = 8.3/5.9 Hz, 1H, 4‐*H*_bdz_), 6.95 (ddd, *J* = 7.9/7.0/1.0 Hz, 1H, 5‐*H*_indole_), 7.05 (ddd, *J* = 8.1/6.9/1.2 Hz, 1H, 6‐*H*_indole_), 7.18 (ddd, *J* = 10.7/8.3/1.1 Hz, 1H, 3‐*H*_FPhe_), 7.24–7.29 (m, 3H, 10‐*H*_bdz_, 5‐*H*_FPhe_, 2‐*H*_indole_), 7.33 (dt, *J* = 8.1/0.9 Hz, 1H, 7‐*H*_indole_), 7.45–7.56 (m, 3H, 9‐*H*_bdz_, 4‐*H*_FPhe_, 6‐*H*_FPhe_), 7.61 (d, *J* = 7.8 Hz, 1H, 4‐*H*_indole_), 7.70–7.75 (m, 1H, 8‐*H*_bdz_), 7.81 (dd, *J* = 8.2/1.2 Hz, 1H, 7‐*H*_bdz_), 10.85 (s, 1H, NH). ^13^C NMR (151 MHz, DMSO‐d_6_): δ (ppm) = 11.6 (*C*H_3_), 27.1 (C*H*_2_), 57.3 (C‐4_bdz_), 111.0 (C‐3_indole_), 111.3 (C‐7_indole_), 116.0 (d, *J* = 21.4 Hz, C‐3_FPhe_), 118.2 (C‐5_indole_), 118.6 (C‐4_indole_), 120.8 (C‐6_indole_), 123.9 (C‐7_bdz_), 124.1 (C‐2_indole_), 124.5 (d, *J* = 3.1 Hz, C‐5_FPhe_), 127.4 (C‐3a_indole_), 127.4 (d, *J* = 12.0 Hz, C‐1_FPhe_), 127.9 (C‐9_bdz_), 129.5 (C‐10a_bdz_), 129.6 (C‐10_bdz_), 131.3 (d, *J* = 2.4 Hz, C‐6_FPhe_), 131.8 (C‐8_bdz_), 131.8 (C‐6a_bdz_), 132.4 (d, *J* = 8.7 Hz, (C‐4_FPhe_), 136.0 (C‐7a_indole_), 150.5 (C‐1_triazole_), 156.4 (C‐3a_bdz_), 159.6 (d, *J* = 249.1 Hz, C‐2_FPhe_), 163.5 (C‐6_bdz_). IR (neat): ν̃ (cm^−1^) = 3059 (C–H, aryl), 2924 (C–H, CH_3_), 1605 (C=C, aryl), 1578 (C=N, triazole). Exact mass (APCI): *m/z* = 422.1746 (calcd 422.1776 for C_26_H_21_FN_5_ [M+H]^+^). 26 mg of the racemic mixture was separated by chiral HPLC (method 1, Supporting Information). *(S)‐* **18:** Colorless solid, yield 13 mg (0.03 mmol, 50%). Purity (HPLC method 1): 98.5%, t_R_ = 19.8 min. Specific rotation: ${\left[\alpha \right]}_{20}^{\text{D}}$ = −46.3 (c = 0.06; CH_3_OH). (*R*)*‐* **18:** Colorless solid, yield 12 mg (0.03 mmol, 46%). Purity (HPLC method 1): 98.6%, t_R_ = 19.8 min. Specific rotation: ${\left[\alpha \right]}_{20}^{\text{D}}$ = +43.2 (c = 0.09; CH_3_OH).

## Conflicts of Interest

The authors declare no conflicts of interest.

## Supporting Information

The Supporting Information contains some general information used for the synthesis, HPLC methods including chiral and preparative HPLC, HPLC chromatograms showing the enantiomeric purity, correlation of retention times, specific rotation and biological activity. experimental procedures of TEVC recordings, details of the pharmacokinetic assays including biotransformation studies, ^1^H and ^13^C NMR spectra and HPLC chromatograms of all synthesized compounds.

## Supporting information

Supporting File

## Data Availability

The data that support the findings of this study are available on request from the corresponding author. The data are not publicly available due to privacy or ethical restrictions.
