# Supplementary material for: Structure–Activity Relationship Study on Ligands Activating the Voltage‐Gated Potassium Channel KV7.1
Source: Arch Pharm (Weinheim). 2026 Jun 5;359(6):e70268. doi: 10.1002/ardp.70268 (PMC13238819; doi:10.1002/ardp.70268)
Supplement: Supplementary file 1 — Supporting File [file ARDP-359-e70268-s001.docx]

**Supporting information**

# Structure-activity relationship study on ligands activating the voltage-gated potassium channel K_V_7.1

Florian Roßner,^(a,b)^ Judith Schmidt,^(a)^ Guiscard Seebohm,^(a,c)^ * Bernhard Wünsch^(a,b)^ *

^[a]^ GRK 2515, Chemical biology of ion channels (Chembion), Universität Münster, Corrensstr. 48, D-48149 Münster, Germany

^[b]^ Universität Münster, Institut für Pharmazeutische und Medizinische Chemie, Corrensstraße 48, D-48149 Münster, Germany.

Tel.: +49-251-8333311; E-mail: [wuensch@uni-muenster.de](mailto:wuensch@uni-muenster.de)

^[c]^ University Hospital Münster, Institute for Genetics of Heart Diseases (IfGH), Department of Cardiovascular Medicine, D-48149 Münster, Germany.

Content: page

1. General methods used in the synthesis part S2

2. HPLC methods S3

3. HPLC chromatograms showing enantiomeric purity S4

4. Summary of retention times, specific rotation and activity of **9b**, *ent*-**9b**, S7

*(R)-***17**, *(S)-***17**, *(R)-***18** and *(S)*-**18**

5. Molecular biology and TEVC measurements S8

6. Determination of pharmacokinetic parameters in vitro S9

7. References S15

8. NMR spectra S16

9. HPLC chromatograms showing the purity of all synthesized compounds S55

**1. General methods used in the synthesis part**

Oxygen and moisture sensitive reactions were carried out under nitrogen, dried with silica gel with moisture indicator (orange gel, VWR, Darmstadt, Germany) and in dry glassware (Schlenk flask or Schlenk tube). Temperature was controlled with ice/water (0 °C), magnetic stirrer MR 3001 K (Heidolph, Schwalbach, Germany) or RCT CL (IKA, Staufen, Germany), together with temperature controller EKT HeiCon (Heidolph) or VT-5 (VWR) and silicone bath. All solvents were of analytical or technical grade quality. Demineralized water was used. CH_2_Cl_2_ was distilled from CaH_2_. Thin layer chromatography (tlc): tlc silica gel 60 F_254_ on aluminum sheets (VWR). Flash chromatography (fc): Silica gel 60, 40–63 µm (VWR); parentheses include: diameter of the column (∅), length of the stationary phase (l) and eluent. Automated flash chromatography: Isolera^TM^ Spektra One (Biotage^®^); parentheses include: cartridge size, eluent, fractions size was always 20 mL. Melting point: Melting point system MP50 (Mettler Toledo, Gießen, Germany), open capillary, uncorrected. MS: MicroTOFQII mass spectrometer (Bruker Daltonics, Bremen, Germany); deviations of the found exact masses from the calculated exact masses were 5 ppm or less; the data were analyzed with DataAnalysis^®^ (Bruker Daltonics). NMR: NMR spectra were recorded in deuterated solvents on Agilent DD2 400 MHz and 600 MHz spectrometers (Agilent, Santa Clara CA, USA); chemical shifts (*δ*) are reported in parts per million (ppm) against the reference substance tetramethylsilane and calculated using the solvent residual peak of the undeuterated solvent; coupling constants are given with 0.5 Hz resolution; assignment of ^1^H and ^13^C NMR signals was supported by 2-D NMR techniques where necessary.IR: FT/IR IR Affinity^®^-1 spectrometer (Shimadzu, Düsseldorf, Germany) using ATR technique. Optical rotation: Polarimeter 341 (Perkin Elmer); 1.0 dm tube; concentration *c* in g/100 mL; T = 20 °C; wavelength 589 nm (D-line of Na light); the unit of the specific rotation ([α]_D_^T^ grad^.^mL^.^dm^-1.^g^-1^) is omitted for clarity.

**2. HPLC methods**

**2.1. Preparative HPLC method 1 for the separation of *(R)*-17 and *(S)*-17 as well as *(R)*-18 and *(S)*-18**

Dionex Ultimate 3000 photodiode array detector controlled by Chromeleon software v. 6.80, Dionex Ultimate 3000 pump, column: Daicel Chiralpak AD, 10 µm, 250 mm / 20 mm, flow rate: 20.00 mL/min; injection: volume: 4.0 mL, detection λ = 254 nm; eluent: iso-hexane : iso-propanol = 80 : 20

**2.2. Analytical chiral HPLC method 2 to determine the enantiomeric purity**

Merck Hitachi equipment; DAD detector: L-7455; interface D-7000, Rheodyne 7725i; pump: L-6200A; data acquisition: HSM-software; guard column: Daicel Chiralpak IA, 5 µm, 10 mm / 4 mm; column: Daicel Chiralpak IA, 5 µm, 250 mm / 4.6 mm; flow rate: 1.00 mL/min; injection: volume: 10.0 µL; detection λ = 254 nm; eluent: *iso*‑hexane : *iso*-propanol. ^(a), (b), (c)^

(a) for **9b** and e*nt-***9b** eluent: *iso*‑hexane : *iso*-propanol 90 : 10

(b) for *(R)-***17** and *(S)*-**17** eluent: *iso*‑hexane : *iso*-propanol = 87 : 13

(c) for *(R)*-**18** and *(S)*-**18** eluent: *iso*‑hexane : *iso*-propanol = 87 : 13 + 0.05 % Et_2_NH

**3. HPLC chromatograms showing enantiomeric purity**


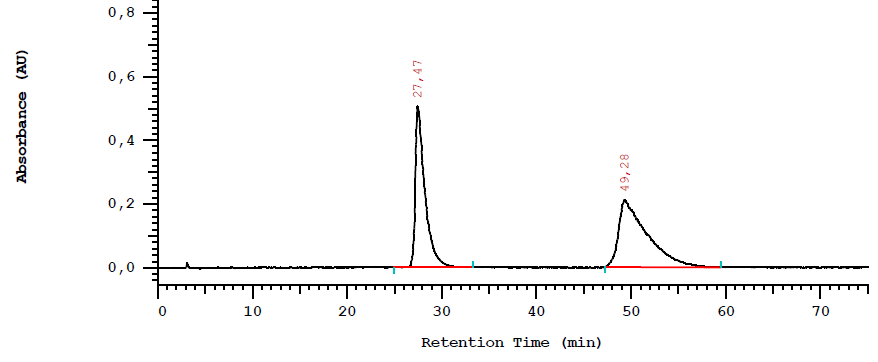

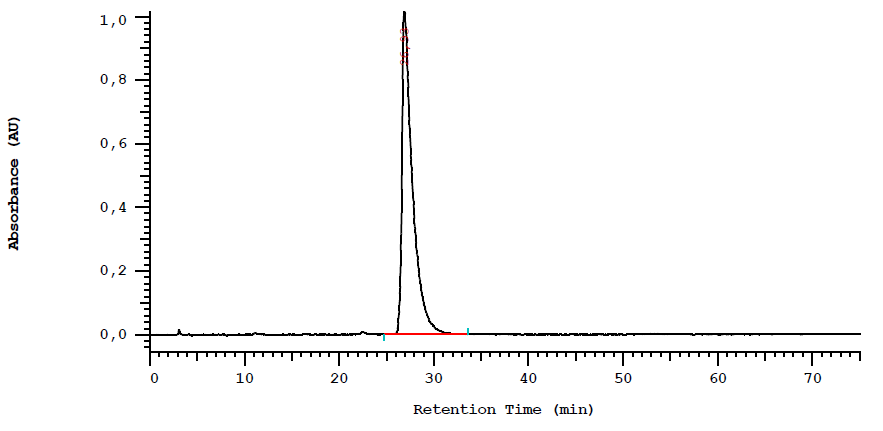

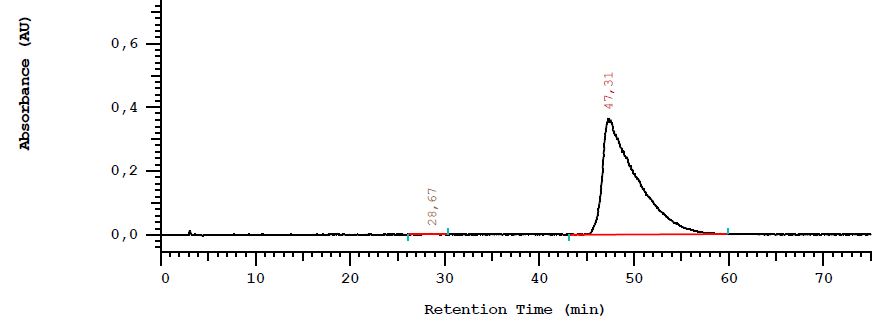


Figure S1: Analytical chiral HPLC (method 2) of enantiomers **9b** and e*nt-***9b**.

Column: Daicel Chiralpak IA (l = 250 mm, d = 4.6 mm); eluent: *iso*‑hexane : *iso*-propanol = 90 : 10; flow rate: 1.0 mL/min; detection λ = 254 nm. *ent-***9b**: 100 % ee; **9b**: 99.9 % ee.


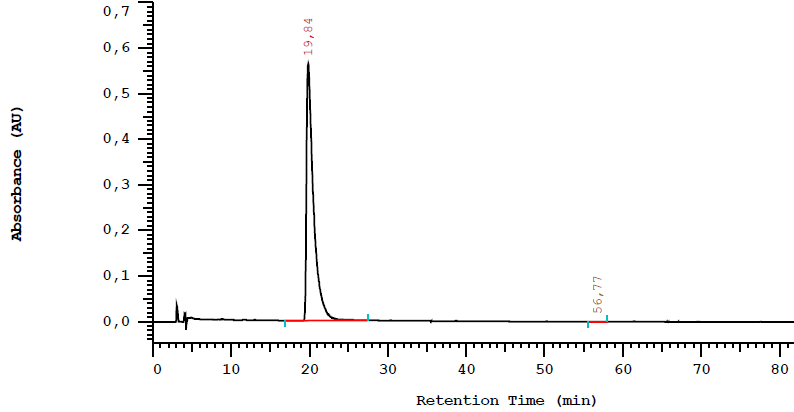

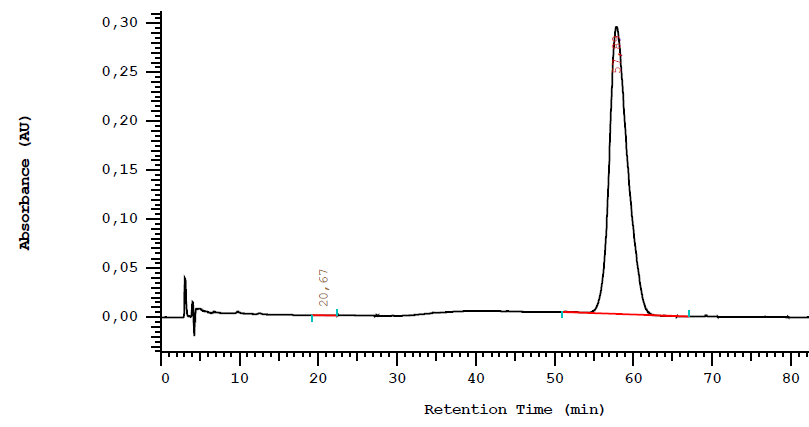

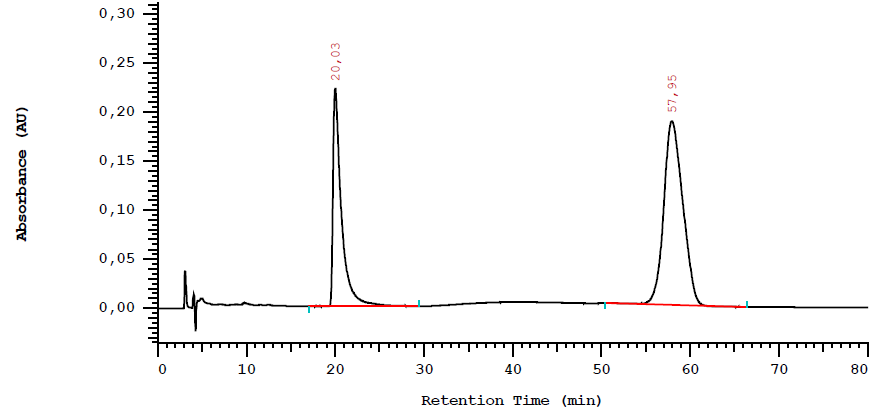


Figure S2: Analytical chiral HPLC (method 2) of enantiomers *(R)*-**17** and *(S)*-**17**.

Column: Daicel Chiralpak IA (l = 250 mm, d = 4.6 mm); eluent: *iso*‑hexane : *iso*-propanol = 87 : 13; flow rate: 1.0 mL/min; detection λ = 254 nm. (*S*)-**17**: > 99.9 % ee; (*R*)-**17**: > 99.9 % ee


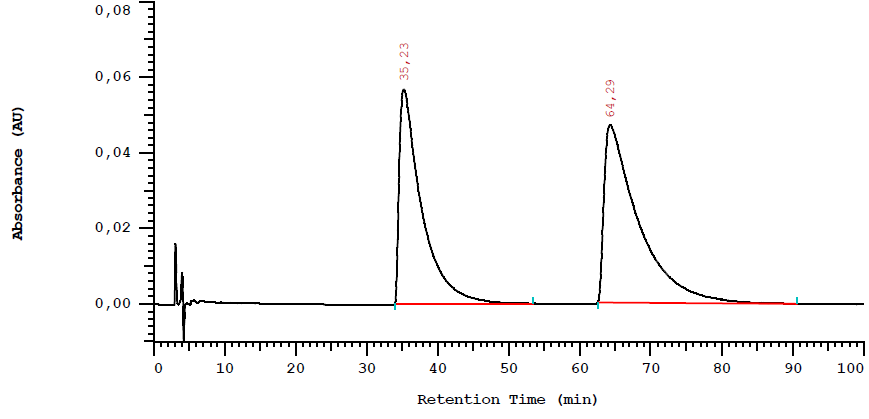

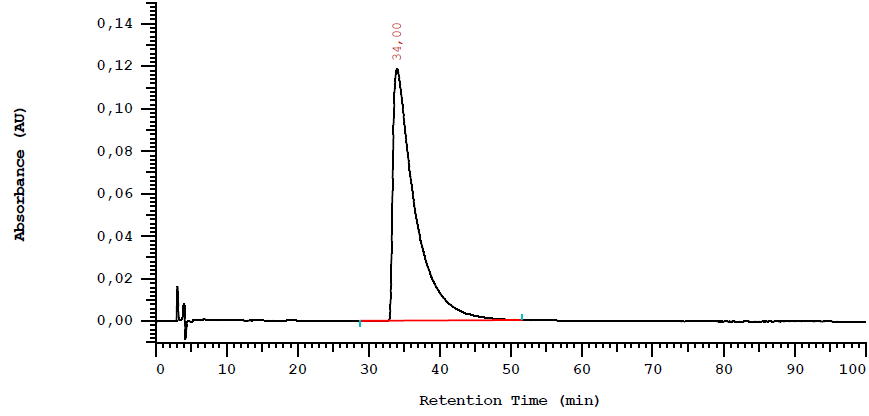

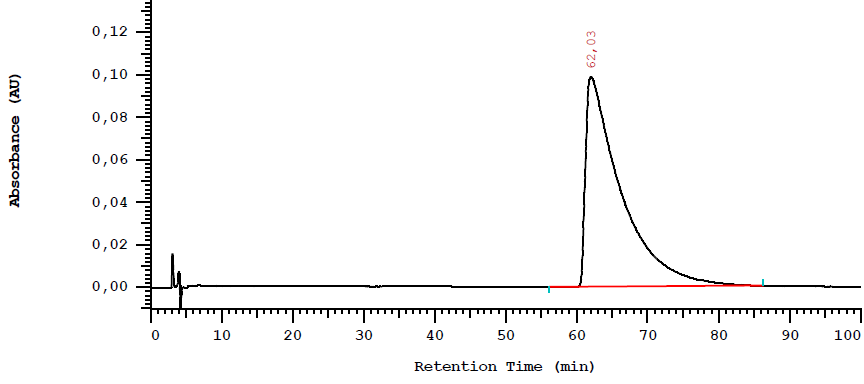


Figure S3: Analytical chiral HPLC (method 2) of enantiomers *(R)*-**18** and *(S)*-**18**.

Column: Daicel Chiralpak IA (l = 250 mm, d = 4.6 mm); eluent: *iso*‑hexane : *iso*-propanol = 87 : 13 + 0.05 % Et_2_NH; flow rate: 1.0 mL/min; detection λ = 254 nm. (*S*)-**18**: 100 % ee; (*R*)-**18**: 100 % ee

**4. Summary of retention times, specific rotation and activity of 9b, *ent*-9b, *(R)-*17, *(S)-*17, *(R)-*18 and *(S)*-18**

Table S1: Retention times on chiral HPLC, specific rotation and activity change of K_V_7.1 ion channels in TEVC-experiments at 10 µM concentration of **9b**, *ent-***9b**, *(R)*-**17**, *(S)*-**17**, *(R)*-**18** and *(S)*-**18**

| compd. | retention t_R_ [min]  on chiral HPLC | specific rotation: ${\text{[}\text{α}\text{]}}_{\text{20}}^{\text{D}}$ | TEVC ^[a]^ Activity change ± SEM [%] |
| --- | --- | --- | --- |
| **9b** | 47.3 | +28.3 | +84 ± 14 |
| *ent-***9b** | 26.4 | -30.3 | -17 ± 11 |
|  |  |  |  |
| *(R)-***17** | 57.9 | +63.9 | +13 ± 7 |
| *(S)-*1**7** | 19.9 | -69.7 | -24 ± 3 |
|  |  |  |  |
| *(R)-***18** | 62.0 | +43.2 | +24 ± 12 |
| *(S)*-**18** | 34.0 | -46.3 | -21 ± 3 |

independent experiments (n > 4).

^[a]^ Activity change on K_V_7.1-expressing oocytes at a test compound concentration of 10 µM compared to activity in buffer solution.

The summary in table 2 shows that all compounds with a longer retention time on chiral HPLC compared to their respective enantiomer are dextrorotatory and cause an activity increase of K_V_7.1 channels in TEVC-experiments. The enantiomers with shorter retention times on a chiral HPLC column are levorotatory and do not increase K_V_7.1 activity. Since the absolute configuration of **9b** *(R)* and *ent-***9b** *(S)* is already known because they are synthesized from (*R*)- or (*S*)-configured amino acids, we hypothesize that the thioamides **17** and triazoles **18** follow the same rules. That means the enantiomers with shorter retention times should be *(S)*-configured and those with longer retention times *(R)*-configured.

**5. Molecular biology and TEVC measurements**

The human KCNQ1 (GenBank^TM^ accession number NM_000218) was subcloned into vector pSGEM, linearized by NHE1 digestion and cRNA was generated using mMessage mMachine kit (Ambion, Austin, TX) according to the manufacturer's instructions as described earlier. ^[1]^

Oocyte preparation for TEVC recordings and the conduction of experiments were performed as described by Schreiber et al. ^[2]^ In brief, Oocytes were isolated from ovarian lobes of Xenopus laevis and enzymatically digested with collagenase (Type II, Worthington, 1 mg/ml in calcium-free Barth’s solution) for about 1.5–2 h. Stage IV or V oocytes were injected with 5 ng cRNA encoding K_V_7.1 subunits and stored for 2–4 days at 18 °C in Barth’s solution containing (in mmol L−1): 88 NaCl, 1.1 KCl, 2.4 NaHCO_3_, 0.3 Ca(NO_3_)_2_, 0.3 CaCl_2_, 0.8 MgSO_4_, 15 HEPES-NaOH, penicillin-G (31 mg L−1), gentamycin (50 mg L−1), streptomycin sulfate (20 mg L−1), pH 7.6 before electrophysiological recording. Whole-cell currents in oocytes were recorded at room temperature by two-electrode voltage-clamp (TEVC) using a Turbo Tec 10CD amplifier (NPI Electronic GmbH, Tamm, Germany) and an Ni-USB-6221 (National Instruments, Austin, Texas, USA) interface combined with GePulse software (Michael Pusch, Genova, Italy). Recording pipettes pulled from borosilicate glass were filled with 3 M KCl and had resistances of 0.5-1.5 MΩ. Recordings were performed in ND96 solution containing (in mmol L−1) 96 NaCl, 4 KCl, 1 CaCl_2_, 1.8 MgCl_2_, 5 HEPES, pH 7.2–7.4 with 0.1% dimethylsulfoxide. To determine the effects of the synthesized compounds on K_V_7.1 channels, currents were recorded by repetitive 2.5-second pulses from −80 mV to +40 mV, applied in 20 mV increments, from a holding potential of -80 mV, followed by a 4.5-second tail pulse at −120 mV. The effect of each compound on K_V_7.1 ion channels is always given as the change of channel activity with the compound in ND96 buffer solution compared to a recording of the same oocyte in only ND96 buffer. Activity was determined from peak tail currents measured at −120 mV after a pulse to +40 mV.

6. Determination of pharmacokinetic parameters in vitro

**6.1 Determination of log*D*_7.4_ value ^[3, 4]^**

*Instruments and parameters for LC-MS standard analysis (in general, if not stated otherwise)*

UPLC-UV/MS (Agilent Technologies): degasser: 1260 HiP (G4225A); pump: 1260 Bin Pump (G1212B); autosampler: 1260 HiP ALS (G1367E); column oven: 1290 TCC (G1316C), 30 °C; UV/Vis detector: 1260 VWD (G1314F); MS-detector: 6120 Quadrupole LC/MS (G1978B). MS source: multimode source (G1978B); ESI mode; SIM mode (*m*/*z* given for each compound). Data acquisition and settings were performed with OpenLab CDS (ChemStation Edition, Agilent). Guard column: Zorbax Eclipse Plus-C18 (Agilent, Waldbronn, Germany) (2.1 mm x 12.5 mm, 5.0 μm particle size). Main column: Zorbax SB-C18 (Agilent, Waldbronn, Germany) (2.1 mm x 50 mm, 1.8 μm particle size). Spray chamber: vaporizer temperature: 200 °C; drying gas: 12 L/min; nebulizer pressure: 40 psi; capillary voltage: 3000 V; corona current: 4 µA; charging voltage: 2000 V; fragmentor voltage: 100 V; drying gas temperature: 250 °C. 2 mL safe lock tubes (Eppendorf), 2 mL LC-MS vials (Agilent).

*LC-MS standard method (LC parameters in general, if not stated otherwise)*

Eluents: solvent A: H_2_O/CH_3_CN 95:5 + 0.1 % formic acid; solvent B: H_2_O/CH_3_CN 5:95 + 0.1 % formic acid; gradient elution (A %): 0 – 2.5 min: gradient from 100 % to 0 %, 2.5 – 3.5 min: 0 %, 3.5 – 4.0 min: gradient from 0 % to 100 %, 4.0 – 8.0 min: 100 %. Change valve position: after 1.0 min the valve was switched from ”waste“ to ”MS source“. Flow rate: 0.4 mL/min. Injection volume: 1.0 µL to 100 µL (given for each compound, 1.0 µL if not stated otherwise).

*Chemicals, solvents and stock solutions*

3-Morpholinopropanesulfonic acid (MOPS) (Fisher Chemical, 372.5 mg, 8.9 mM) and MOPS sodium salt (Sigma-Aldrich, 513.4 mg, 11.1 mM) were dissolved in dist. H_2_O (200 mL) to prepare a 20 mM buffer solution with pH 7.4. A mixture of *n*‑octanol (Sigma Aldrich) and MOPS buffer (20 mM, pH 7.4) in the ratio 1:1 was stirred overnight at room temperature (500 rpm) to saturate both liquids with each other. Afterwards, the aqueous and organic layers were separated.

10 mM stock solutions of the test compounds in DMSO (MERCK-Schuchardt, Hohenbrunn, Germany) were prepared by dissolving an exactly weighted amount of the test compound and adding the calculated amount of DMSO. Depending on the lipophilicity, either the 10 mM stock solution was used directly or the stock solution was diluted 1:100 with MOPS buffer to a concentration of 100 µM.

*General procedure*

In order to determine the log*D*_7.4_ value, the micro shake flask method was used. ^[3, 4]^ To create physiological conditions a buffer with pH 7.4 was used to analyze the lipophilicity (log*D*_7.4_). The log*D*_7.4_ value was determined by using three different volume ratios of buffer and *n*-octanol (1:1, 2:1, 1:2).

Method LA (standard procedure): The 10 mM DMSO stock solution of the test compound (7.5 µL) was added to three different volumes of MOPS buffer (750 µL, 1000 µL, 500 µL) in 2 mL Eppendorf tubes. Afterwards, the tubes were filled up to 1500 µL with *n*-octanol (750 µL, 500 µL, 1000 µL). Each ratio was produced as a triplicate. The tubes were vortexed at rt and centrifuged at 4 °C with 16,000 rpm for 2 min.

Method LB (for very hydrophilic compounds): The 100 µM MOPS solution of the test compound (75 µL) was added to three different volumes of MOPS buffer (675 µL, 925 µL, 425 µL) in 2 mL Eppendorf tubes. *n*-Octanol was added to fill up the tubes to a total volume of 1500 µL (750 µL, 500 µL, 1000 µL). Each ratio was produced as a triplicate. Afterwards, the tubes were vortexed at rt and centrifuged at 4 °C with 16,000 rpm for 2 min.

An aliquot of the aqueous layer was analyzed by LC-MS standard method. For matrix-matched calibration to calculate log*D*_7.4_ value, the samples were diluted with MOPS buffer within a range of 1.56 nM to 1.0 µM or 39 nM to 10 µM. All samples were measured once.

**6.2 Metabolic stability *in vitro* ^[3, 5]^**

*Preparation of mouse liver microsomes*

Frozen livers (-80 °C) from male C57BL/6 mice were received from *Prof. Dr. Martina Düfer* from the Institute of Pharmaceutical and Medicinal Chemistry (WWU Münster).

At first, the frozen livers were warmed up at 37 °C for a few min and washed with 1.15 % (*m*/*v*) KCl solution at 4 °C. After cutting the livers into small pieces, the livers were homogenized in an Elvehjem-Potter (10 strokes, 3 s, 800 rpm) with cold phosphate buffer (pH 7.4, 0.1 M, 1.0 mL PBS/g liver) containing sodium EDTA (0.5 mM). PBS (pH 7.4, 0.1 M, 3.0 mL PBS/g liver), cooled on ice, was added and the resulting suspension was centrifuged at 9,000 g for 20 min at 4 °C. The supernatant was centrifuged again at 40,000 g for 90 min at 4 °C. The obtaining microsome pellet was dissolved in PBS (pH 7.4, 0.1 M). Aliquots of 1.0 mL were filled in safe lock Eppendorf tubes and stored at -80 °C.

*Instruments and parameters for LC-MS standard analysis (in general, if not stated otherwise)*

UPLC-UV/MS (Agilent Technologies): degasser: 1260 HiP (G4225A); pump: 1260 Bin Pump (G1212B); autosampler: 1260 HiP ALS (G1367E); column oven: 1290 TCC (G1316C), 30 °C; UV/Vis detector: 1260 VWD (G1314F); MS-detector: 6120 Quadrupole LC/MS (G1978B). MS source: multimode source (G1978B); ESI mode; SIM mode (*m*/*z* given for each compound). Data acquisition and settings were performed with OpenLab CDS (ChemStation Edition, Agilent). Guard column: Zorbax Eclipse Plus-C18 (Agilent, Waldbronn, Germany) (2.1 mm x 12.5 mm, 5.0 μm particle size). Main column: Zorbax SB-C18 (Agilent, Waldbronn, Germany) (2.1 mm x 50 mm, 1.8 μm particle size). Spray chamber: vaporizer temperature: 200 °C; drying gas: 12 L/min; nebulizer pressure: 40 psi; capillary voltage: 3000 V; corona current: 4 µA; charging voltage: 2000 V; fragmentor voltage: 100 V; drying gas temperature: 250 °C. 2 mL safe lock tubes (Eppendorf), 2 mL LC-MS vials (Agilent).

*LC-MS standard method (LC parameters in general, if not stated otherwise)*

Eluents: solvent A: H_2_O/CH_3_CN 95:5 + 0.1 % formic acid; solvent B: H_2_O/CH_3_CN 5:95 + 0.1 % formic acid; gradient elution (A %): 0 – 2.5 min: gradient from 100 % to 0 %, 2.5 – 3.5 min: 0 %, 3.5 – 4.0 min: gradient from 0 % to 100 %, 4.0 – 8.0 min: 100 %. Change valve position: after 1.0 min the valve was switched from ”waste“ to ”MS source“. Flow rate: 0.4 mL/min. Injection volume: 1.0 µL to 100 µL (given for each compound, 1.0 µL if not stated otherwise).

*Chemicals, solvents and stock solutions*

NADPH Na_4_ (Carl Roth, Karlsruhe, Germany) and UDPGA Na_3_ (Sigma Aldrich) were dissolved in phosphate buffer (PBS, 0.1 M, pH 7.4, Sigma Aldrich, Darmstadt, Germany) to prepare a 2.0 mg/mL solution, respectively. MgCl_2_ (Honeywell Specialty Chemicals, Seelze, Germany) was dissolved in bidist. H_2_O to a 0.05 M solution. 1.0 mM solutions of the test compounds were prepared from the 10 mM DMSO stock solutions by diluting 1:10 with DMSO (MERCK-Schuchardt, Hohenbrunn, Germany).

*Phase I metabolism*

NADPHNa_4_ (2.0 mg/mL in 0.1 M PBS, 50 µL), MgCl_2_ (0.05 M in H_2_O, 50 µL) and phosphate buffer (PBS, 0.1 M, 76.8 µL) were mixed in an Eppendorf tube. The test compound (1.0 mM in DMSO, 1.2 µL) and mouse liver microsomes (MLM, 22 µL) were added. Instead of test compound, imipramine (1.0 mM in DMSO, 1.2 µL) was incubated with mouse liver microsomes as positive control. The metabolic stability of imipramine using this procedure is well known (20 % of parent compound after 90 min incubation). The prepared samples were incubated at 37 °C for 90 min at 900 rpm at the thermomixer (Eppendorf). The incubation was stopped by adding CH_3_CN/CH_3_OH 1:1 (400 µL) to the samples and ice-cooling for 10 min to precipitate the proteins. The samples were centrifuged at 4 °C for 15 min at 16,000 rpm. An aliquot of the supernatant was measured by LC-MS standard method. An “empty sample” (without test compound, PBS was added to replace the missing volume) was prepared in the same way. Additionally, “blanks” (without NADPH Na_4_, PBS was added to replace the missing volume) were prepared according to the same procedure. The test compound (10 mM in DMSO, 1.2 µL) was added after precipitating the proteins under ice-cooling for 10 min.

**6.3 Plasma protein binding (PPB) ^[3, 6, 7]^**

*Instruments and parameters for LC-MS PPB analysis (in general, if not stated otherwise)*

UPLC-UV/MS (Agilent Technologies): degasser: 1260 HiP (G4225A); pump: 1260 Bin Pump (G1212B); autosampler: 1260 HiP ALS (G1367E); column oven: 1290 TCC (G1316C), 30 °C; UV/Vis detector: 1260 VWD (G1314F); MS-detector: 6120 Quadrupole LC/MS (G1978B). MS source: multimode source (G1978B); ESI mode; SIM mode (*m*/*z* given for each compound). Data acquisition and settings were performed with OpenLab CDS (ChemStation Edition, Agilent). Guard column: Chiralpak^®^ HSA HPLC guard column (2.0 x 10 mm, 5.0 μm particle size). Main column: Chiralpak^®^ HSA HPLC column (Daicel, Eschborn, Germany) (2.0 x 50 mm, 5.0 μm particle size). Spray chamber: vaporizer temperature: 200 °C; drying gas: 12 L/min; nebulizer pressure: 40 psi; capillary voltage: 3000 V; corona current: 4 µA; charging voltage: 2000 V; fragmentor voltage: 100 V; drying gas temperature: 250 °C. 2 mL safe lock tubes (Eppendorf), 2 mL LC-MS vials (Agilent).

*LC-MS PPB method* *(LC parameters in general, if not stated otherwise)*

Eluents: solvent A: NH_4_OAc buffer (50 mM, pH 7.4); solvent B: *iso*propanol; isocratic elution (A %): 96 %. Flow rate: 0.3 mL/min. Stop time: 3.0 min (*D*‑glucose, metronidazole, paracetamol, ramipril, salbutamol, sulfamethoxazole), 8.0 min (propranolol), 10 min (phenytoin), 15 min (haloperidol, imipramine), 60 min (chlorpromazine, test compounds). Injection volume: 1.0 µL to 100 µL (given for each compound, 1.0 µL if not stated otherwise).

*Chemicals, solvents and stock solutions*

The pH value of an aqueous solution of NH_4_OAc (50 mM, Acros Organics, Schwerte, Germany) was adjusted to pH 7.4 with NH_3_. 2.0 mM solutions of the reference and the test compounds were prepared from the 10 mM DMSO stock solutions by diluting 1:5 with DMSO (MERCK-Schuchardt, Hohenbrunn, Germany). The final concentration of 20 µM was prepared by further dilution 1:100 with eluent NH_4_OAc (50 mM, pH 7.4) : *iso*propanol = 96:4.

*Method PA (general procedure)*

In order to determine the PPB, a high performance affinity chromatography (HPAC) was performed. The dead time was determined with *D*-glucose. The following compounds with known plasma protein binding were used as reference compounds: metronidazole, paracetamol, salbutamol, sulfamethoxazole, ramipril, propranolol, phenytoin, haloperidol, imipramine and chlorpromazine. The retention times of these compounds and the test compound were measured three times by LC-MS PPB method in SIM mode.

**7. References**

[1] K. Eckey, E. Wrobel, N. Strutz-Seebohm, L. Pott, N. Schmitt, G. Seebohm, *J. Biol. Chem.*, **2014**, 33, 22749–22758

[2] J. A. Schreiber, M. Möller, M. Zaydman, L. Zhao, Z. Beller, S. Becker, N. Ritter, P. Hou, J. Shi, J. Silva, E. Wrobel, N. Strutz-Seebohm, N. Decher, N. Schmitt, S. G. Meuth, M. Düfer, B. Wünsch, J, Cui, G. Seebohm, *Commun. Biol.*, **2022**, 5, 301–313

[3] F. Börgel, F. Galla, K. Lehmkuhl, D. Schepmann, S. M. Ametamey, B. Wünsch, *J. Pharm. Biomed. Anal.,* **2019**, 172, 214–222

[4] F. Galla, C. Bourgeois, K. Lehmkuhl, D. Schepmann, M. Soeberdt, T. Lotts, C. Abels, S. Ständer, B. Wünsch, *Med. Chem. Commun.,* **2016**, 7, 317–326

[5] C. Wiese, E. Große Maestrup, F. Galla, D. Schepmann, A. Hiller, S. Fischer, F.-A. Ludwig, W. Deuther-Conrad, C. K. Donat, P. Brust, L. Büter, U. Karst, B. Wünsch, *ChemMedChem*., **2016**, 11, 2445–2458

[6] V. Butsch, F. Börgel, F. Galla, K. Schwegmann, S. Hermann, M. Schäfers, B. Riemann, B. Wünsch, S. Wagner, *J. Med. Chem*., **2018**, 61, 4115–4134

[7] C. P. Konken, K. Heßling, I. Thale, S. Schelhaas, J. Dabel, S. Maskri, E. Bulk, T. Budde, O. Koch, A. Schwab, M. Schäfers, B. Wünsch, *Arch. Pharm. (Weinheim).,* **2022**, 355

**8. NMR spectra**

^1^H NMR spectrum of **4b** in DMSO-d6

^13^C NMR spectrum of **4b** in DMSO-d6

^1^H NMR spectrum of **4c** in DMSO-d6

^13^C NMR spectrum of **4c** in DMSO-d6

^1^H NMR spectrum of **4d** in DMSO-d6****

^13^C NMR spectrum of **4d** in DMSO-d6

^1^H NMR spectrum of **4e** in DMSO-d6

^13^C NMR spectrum of **4e** in DMSO-d6

^1^H NMR spectrum of **4f** in DMSO-d6

^13^C NMR spectrum of **4f** in DMSO-d6

^1^H NMR spectrum of **4g** in DMSO-d6

^13^C NMR spectrum of **4g** in DMSO-d6

^1^H NMR spectrum of **5** in DMSO-d6

^13^C NMR spectrum of **5** in DMSO-d6

^1^H NMR spectrum of **4h** in DMSO-d6

^13^C NMR spectrum of **4h** in DMSO-d6

^1^H NMR spectrum of **8a** in DMSO-d6

^13^C NMR spectrum of **8a** in DMSO-d6

^1^H NMR spectrum of **8b** in DMSO-d6

^13^C NMR spectrum of **8b** in DMSO-d6

^1^H NMR spectrum of *ent-***8b** in DMSO-d6

^13^C NMR spectrum of *ent-***8b** in DMSO-d6

^1^H NMR spectrum of **8c** in DMSO-d6

^13^C NMR spectrum of **8c** in DMSO-d6

^1^H NMR spectrum of **8d** in DMSO-d6

^13^C NMR spectrum of **8d** in DMSO-d6

^1^H NMR spectrum of **8e** in DMSO-d6

^13^C NMR spectrum of **8e** in DMSO-d6

^1^H NMR spectrum of **8f** in DMSO-d6

^13^C NMR spectrum of **8f** in DMSO-d6

^1^H NMR spectrum of **8g** in DMSO-d6

^13^C NMR spectrum of **8g** in DMSO-d6

^1^H NMR spectrum of **8h** in DMSO-d6

^13^C NMR spectrum of **8h** in DMSO-d6

^1^H NMR spectrum of **8i** in DMSO-d6

^13^C NMR spectrum of **8i** in DMSO-d6

^1^H NMR spectrum of **12a** in DMSO-d6

^13^C NMR spectrum of **12a** in DMSO-d6

^1^H NMR spectrum of *ent-*1**2a** in DMSO-d6

^13^C NMR spectrum of *ent-*1**2a** in DMSO-d6

**
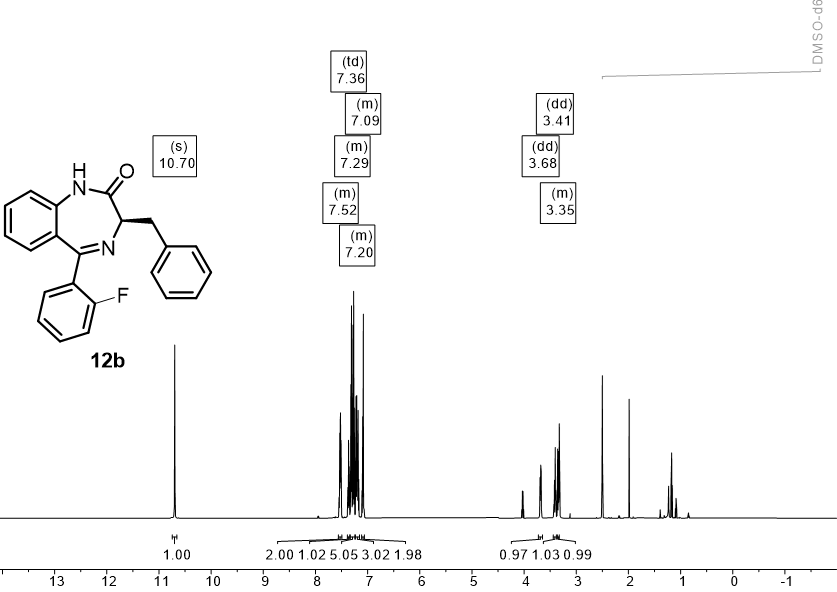
**

^1^H NMR spectrum of **12b** in DMSO-d6

^13^C NMR spectrum of **12b** in DMSO-d6

^1^H NMR spectrum of **9b** in DMSO-d6

^13^C NMR spectrum of **9b** in DMSO-d6

^1^H NMR spectrum of e*nt-***9b** in DMSO-d6

^13^C NMR spectrum of e*nt-***9b** in DMSO-d6

^1^H NMR spectrum of **9c** in DMSO-d6

^13^C NMR spectrum of **9c** in DMSO-d6

^1^H NMR spectrum of **9d** in DMSO-d6

^13^C NMR spectrum of **9d** in DMSO-d6

^1^H NMR spectrum of **9e** in DMSO-d6

^13^C NMR spectrum of **9e** in DMSO-d6

^1^H NMR spectrum of **9h** in DMSO-d6

^13^C NMR spectrum of **9h** in DMSO-d6

^1^H NMR spectrum of **13a** in DMSO-d6

^13^C NMR spectrum of **13a** in DMSO-d6

^1^H NMR spectrum of *ent-***13a** in DMSO-d6

^13^C NMR spectrum of *ent*-**13a** in DMSO-d6

^1^H NMR spectrum of **9i** in DMSO-d6

^13^C NMR spectrum of **9i** in DMSO-d6

^1^H NMR spectrum of **14** in DMSO-d6

^13^C NMR spectrum of **14** in DMSO-d6

^1^H NMR spectrum of **15** in DMSO-d6

^13^C NMR spectrum of **15** in DMSO-d6

^1^H NMR spectrum of **16** in DMSO-d6

^13^C NMR spectrum of **16** in DMSO-d6

^1^H NMR spectrum of *rac-***17** in DMSO-d6

^13^C NMR spectrum of *rac*-**17** in DMSO-d6

^1^H NMR spectrum of *(R)-***17** in DMSO-d6

^13^C NMR spectrum of *(R)*-**17** in DMSO-d6

^1^H NMR spectrum of *(S)-***17** in DMSO-d6

^13^C NMR spectrum of *(S)*-**17** in DMSO-d6

^1^H NMR spectrum of *rac-***18** in DMSO-d6

^13^C NMR spectrum of *rac*-**18** in DMSO-d6

^1^H NMR spectrum of *(R)-***18** in DMSO-d6

^13^C NMR spectrum of *(R)*-**18** in DMSO-d6

^1^H NMR spectrum of *(S)-***18** in DMSO-d6

^13^C NMR spectrum of *(S)*-**18** in DMSO-d6

**9. HPLC chromatograms showing the purity of all synthesized compounds**


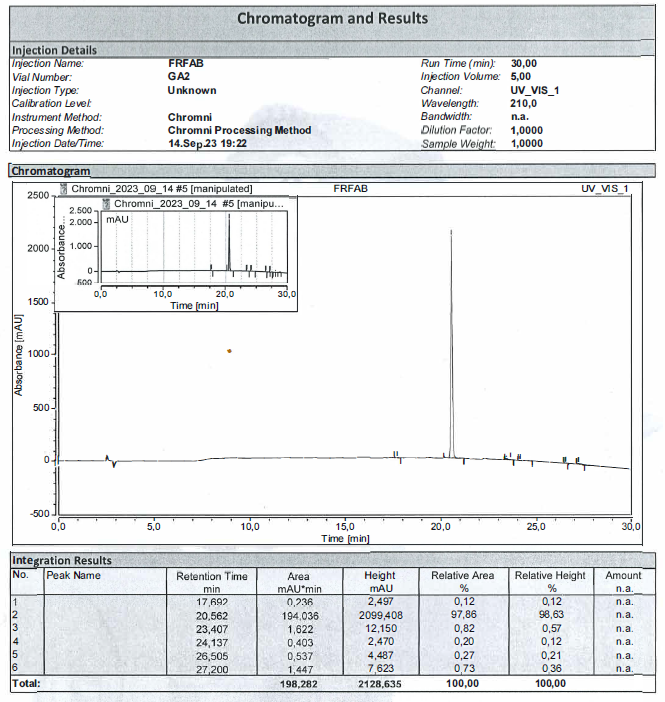


HPLC chromatogram of **4b** showing the purity of **4b** (t_R_ = 20.6 min, method 1).


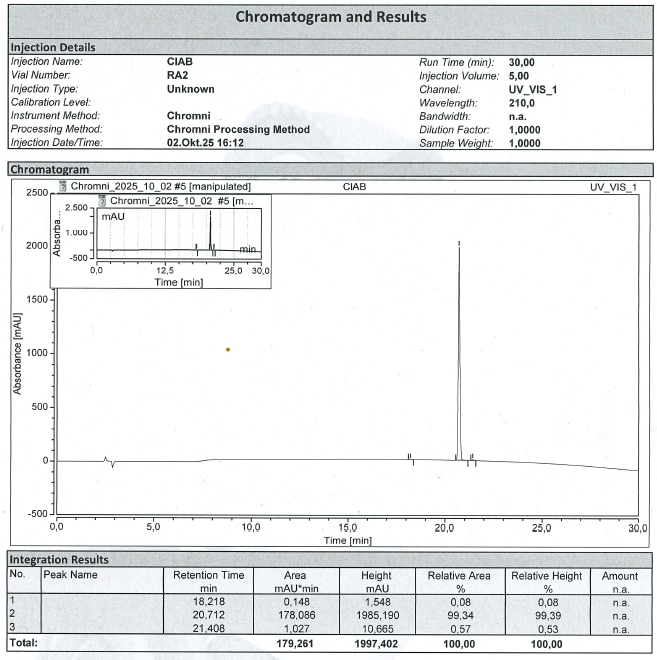


HPLC chromatogram of **4c** showing the purity of **4c** (t_R_ = 20.7 min, method 1).


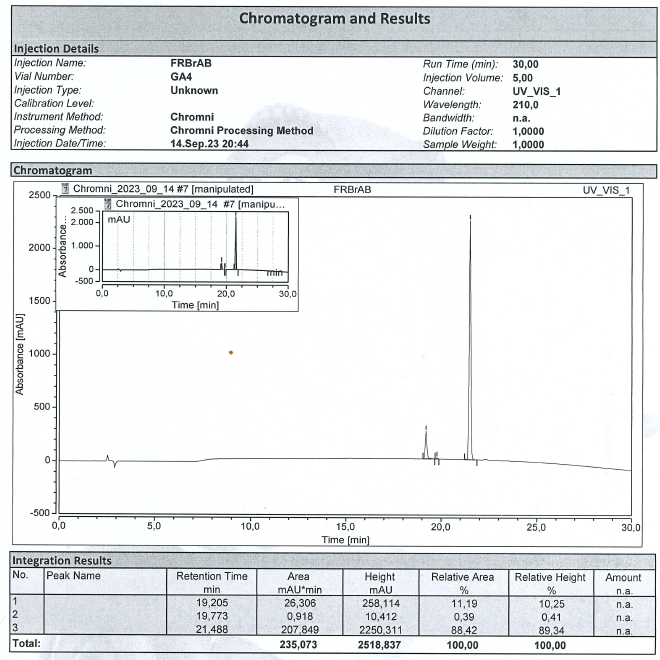


HPLC chromatogram of **4d** showing the purity of **4d** (t_R_ = 21.5 min, method 1).


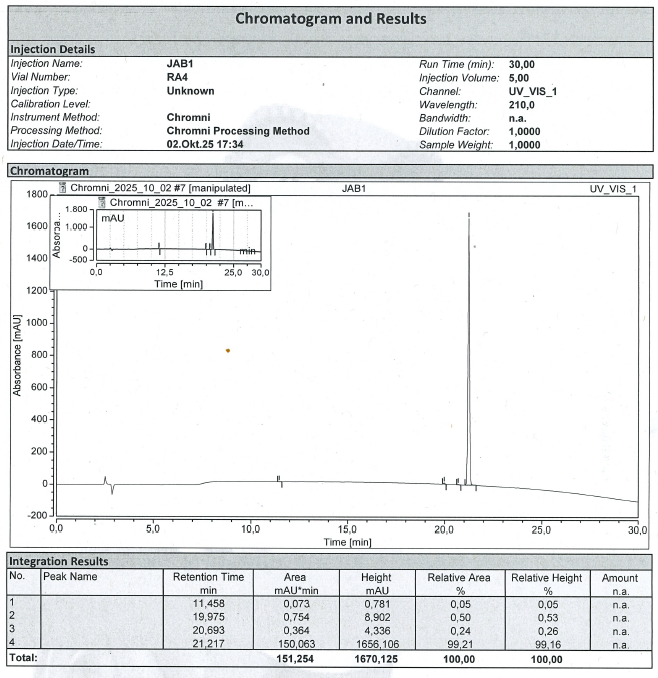


HPLC chromatogram of **4e** showing the purity of **4e** (t_R_ = 21.2 min, method 1).


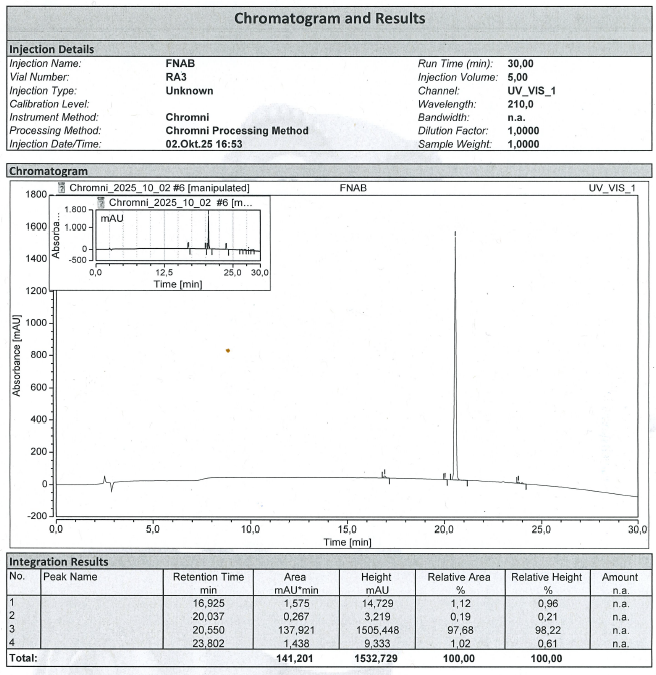


HPLC chromatogram of **4f** showing the purity of **4f** (t_R_ = 20.6 min, method 1).


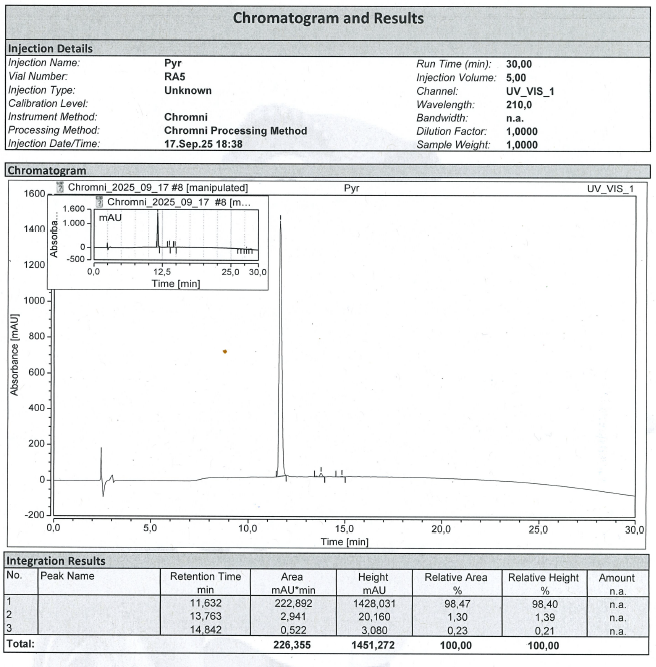


HPLC chromatogram of **4g** showing the purity of **4g** (t_R_ = 11.6 min, method 1).


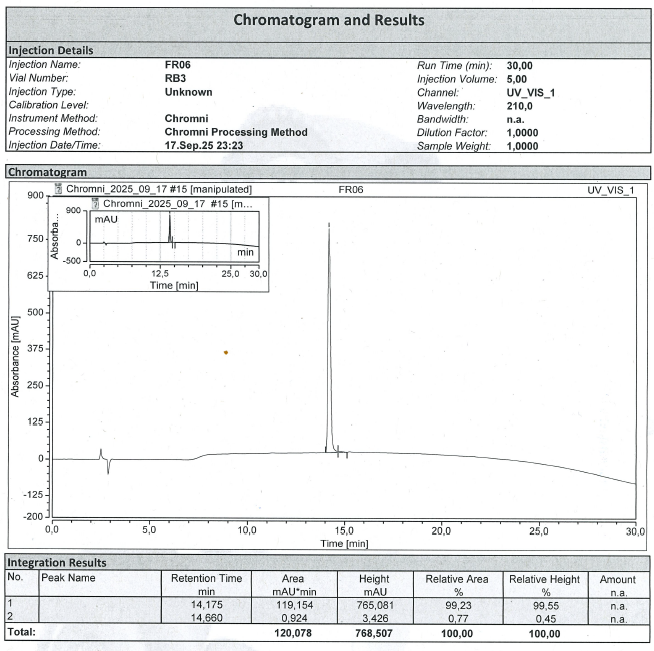


HPLC chromatogram of **5** showing the purity of **5** (t_R_ = 14.2 min, method 1).


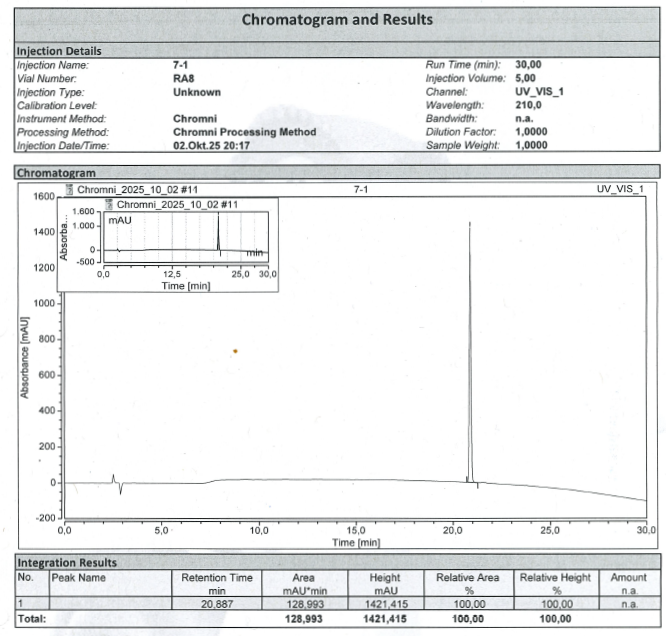


HPLC chromatogram of **4h** showing the purity of **4h** (t_R_ = 20.9 min, method 1).


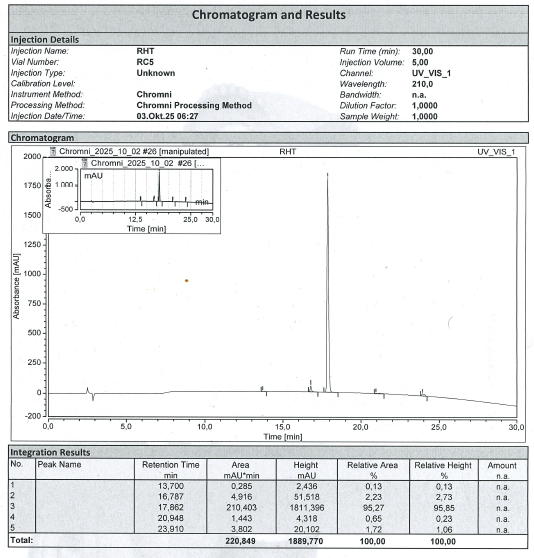


HPLC chromatogram of **8a** showing the purity of **8a** (t_R_ = 17.9 min, method 1).


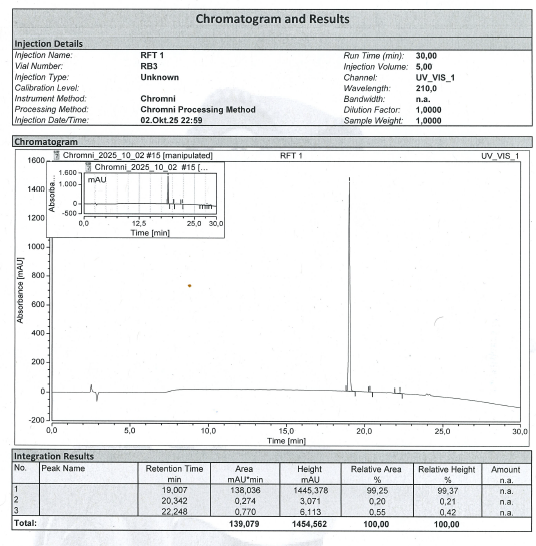


HPLC chromatogram of **8b** showing the purity of **8b** (t_R_ = 19.0 min, method 1).


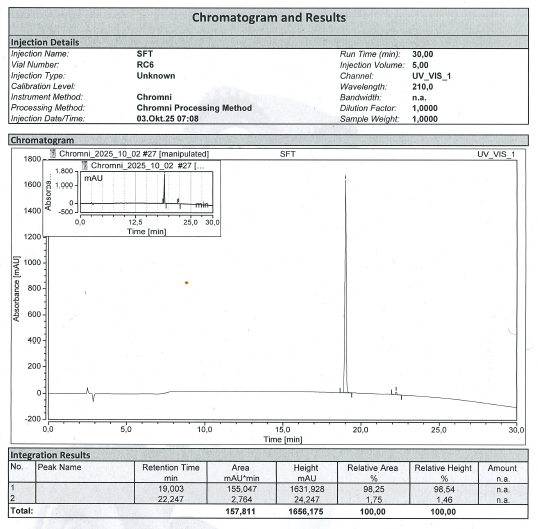


HPLC chromatogram of *ent*-**8b** showing the purity of *ent*-**8b** (t_R_ = 19.0 min, method 1).


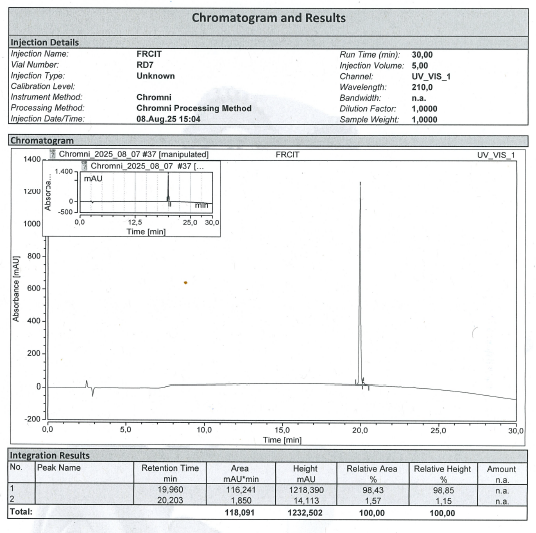


HPLC chromatogram of **8c** showing the purity of **8c** (t_R_ = 20.0 min, method 1).


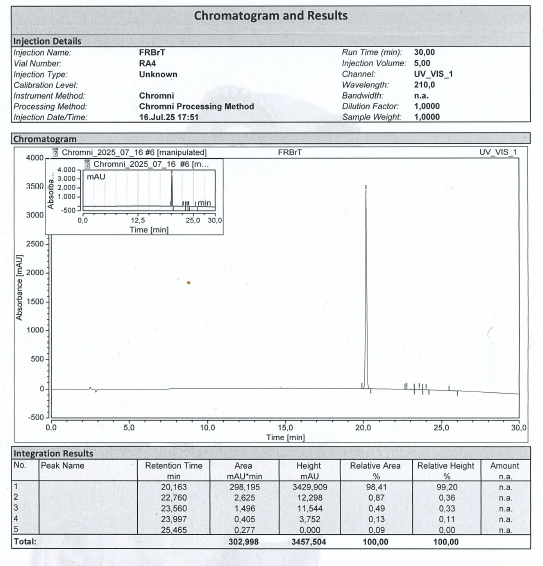


HPLC chromatogram of **8d** showing the purity of **8d** (t_R_ = 20.2 min, method 1).


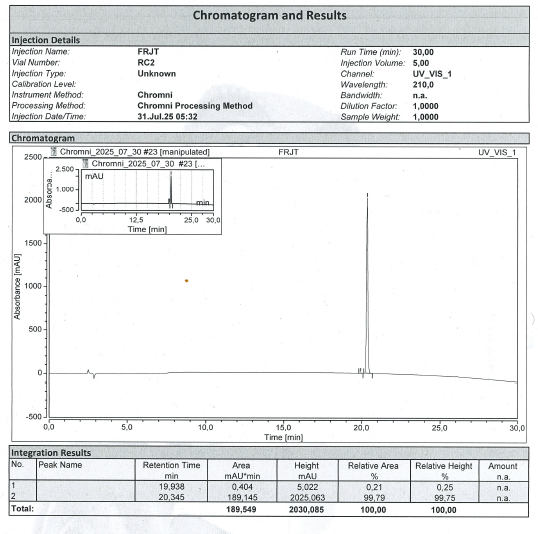


HPLC chromatogram of **8e** showing the purity of **8e** (t_R_ = 20.3 min, method 1).


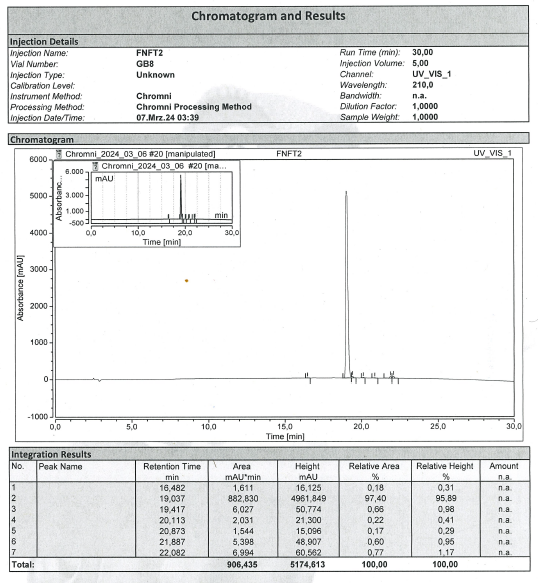


HPLC chromatogram of **8f** showing the purity of **8f** (t_R_ = 19.0 min, method 1).


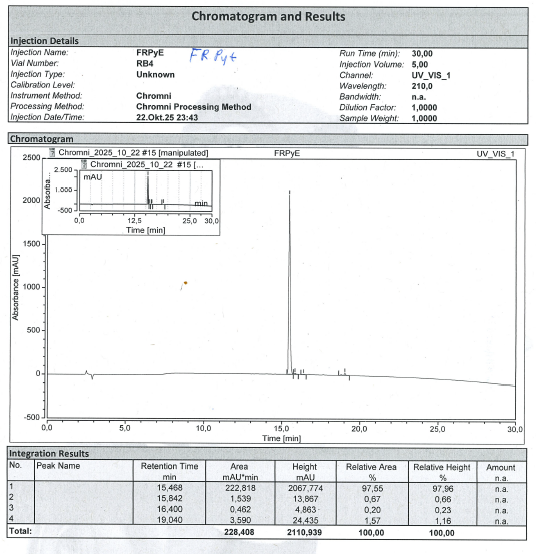


HPLC chromatogram of **8g** showing the purity of **8g** (t_R_ = 15.5 min, method 1).


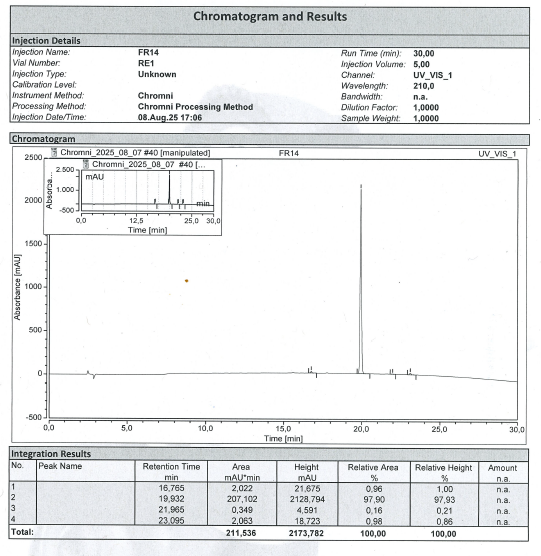


HPLC chromatogram of **8h** showing the purity of **8h** (t_R_ = 19.9 min, method 1).


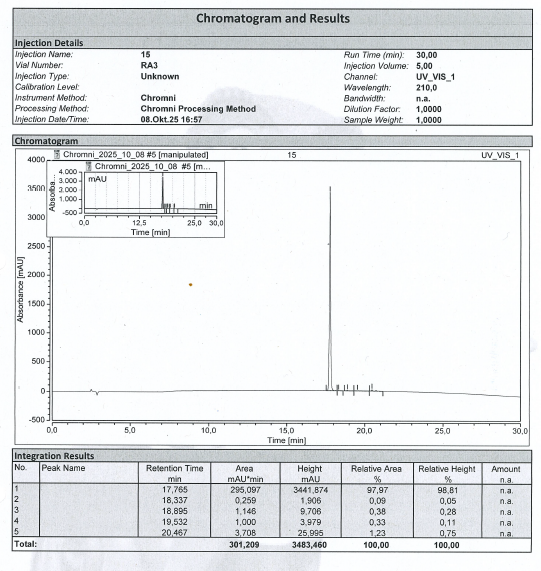


HPLC chromatogram of **8i** showing the purity of **8i** (t_R_ = 17.8 min, method 1).


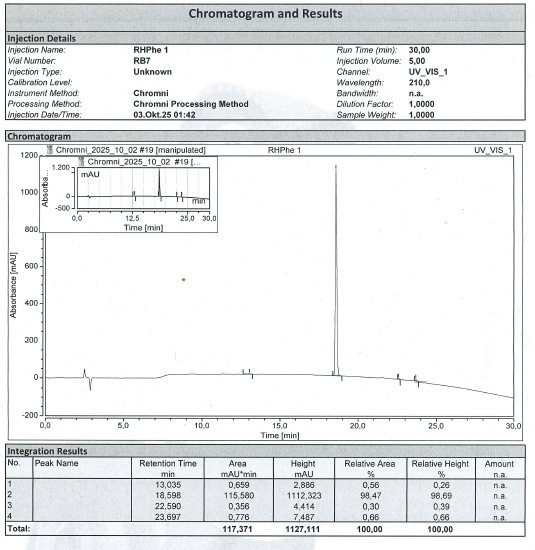


HPLC chromatogram of **12a** showing the purity of **12a** (t_R_ = 18.6 min, method 1).


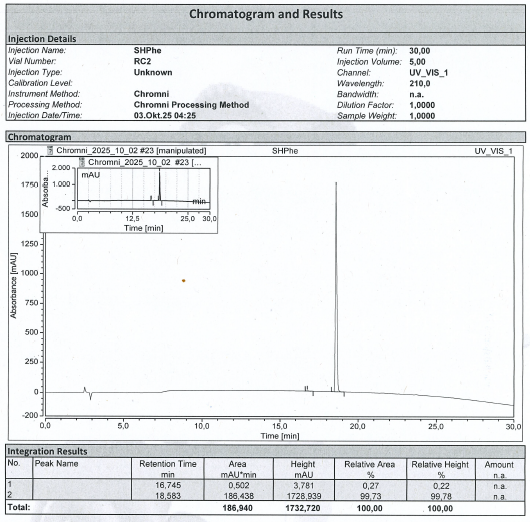


HPLC chromatogram of e*nt-***12a** showing the purity of *ent*-**12a** (t_R_ = 18.6 min, method 1).


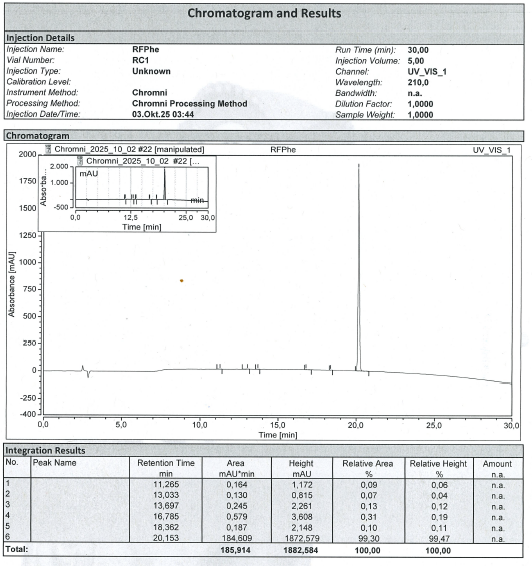


HPLC chromatogram of **13a** showing the purity of **13a** (t_R_ = 20.2 min, method 1).


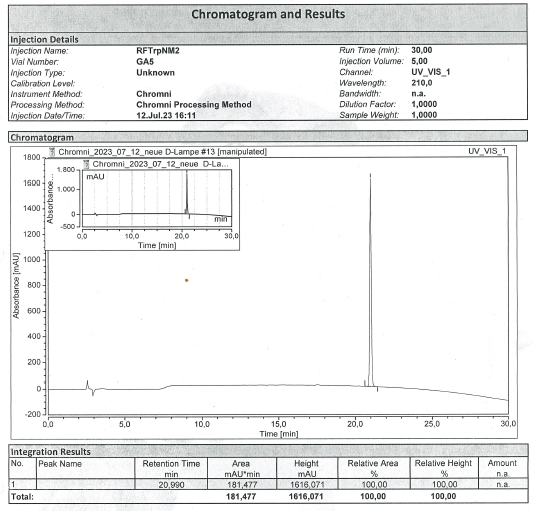


HPLC chromatogram of **9b** showing the purity of **9b** (t_R_ = 21.0 min, method 1).


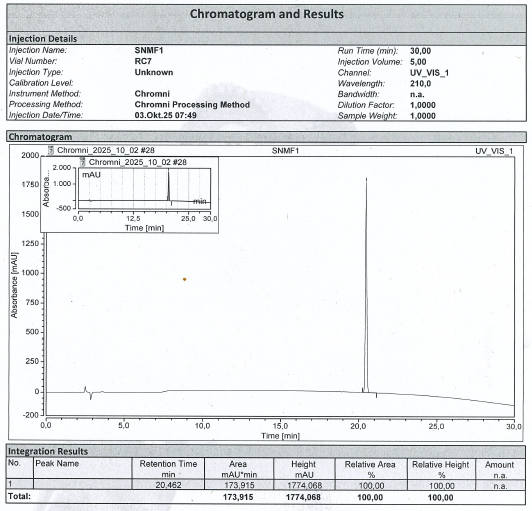


HPLC chromatogram of *ent-***9b** showing the purity of *ent*-**9b** (t_R_ = 20.5 min, method 1).


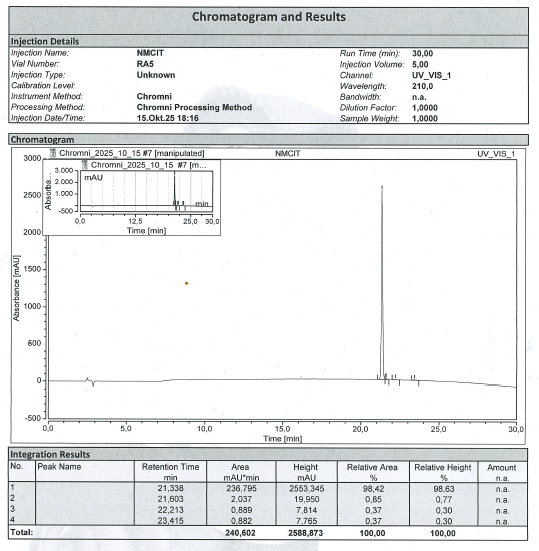


HPLC chromatogram of **9c** showing the purity of **9c** (t_R_ = 21.3 min, method 1).


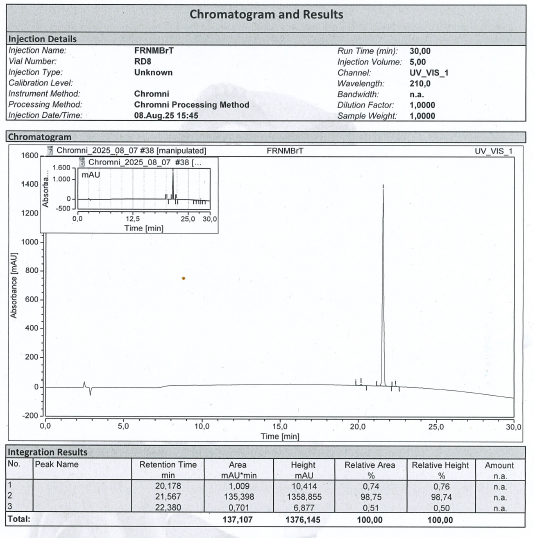


HPLC chromatogram of **9d** showing the purity of **9d** (t_R_ = 21.6 min, method 1).


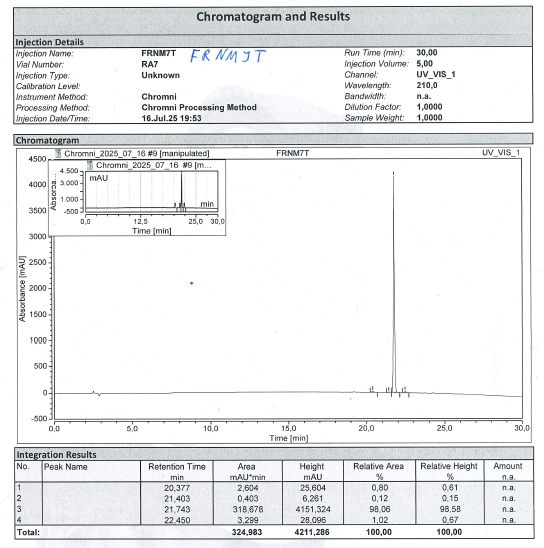


HPLC chromatogram of **9e** showing the purity of **9e** (t_R_ = 21.7 min, method 1).


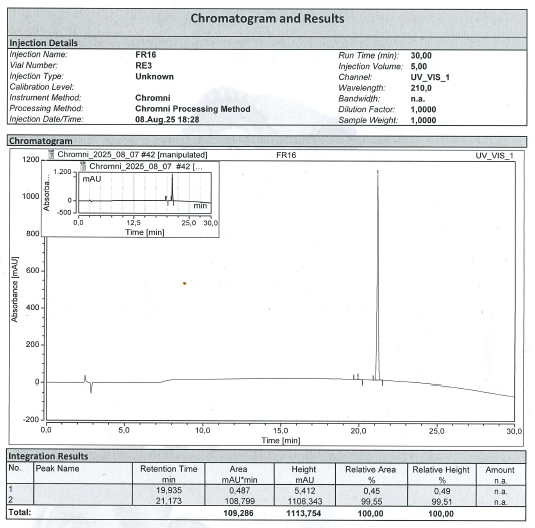


HPLC chromatogram of **9h** showing the purity of **9h** (t_R_ = 21.2 min, method 1).


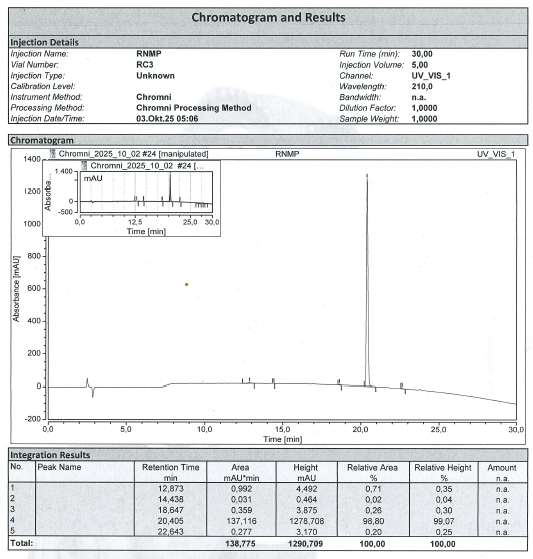


HPLC chromatogram of **13a** showing the purity of **13a** (t_R_ = 20.4 min, method 1).


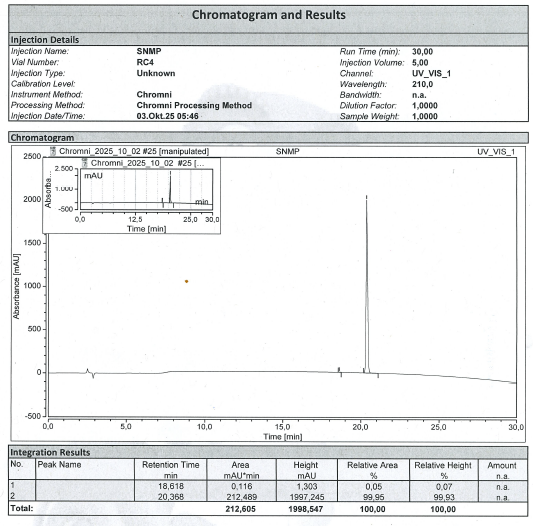


HPLC chromatogram of *ent-***13a** showing the purity of *ent-***13a** (t_R_ = 20.4 min, method 1).


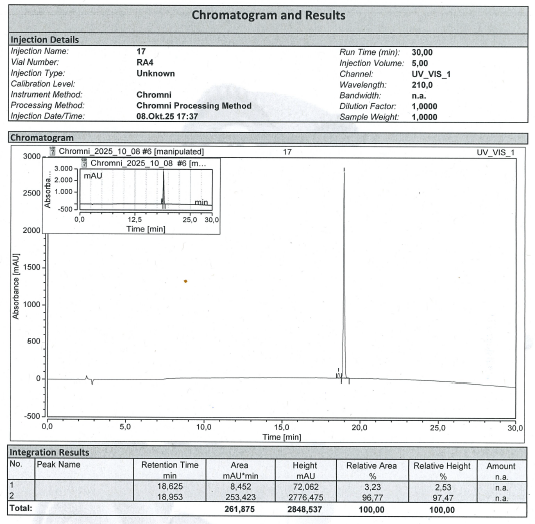


HPLC chromatogram of **9i** showing the purity of **9i** (t_R_ = 19.0 min, method 1).


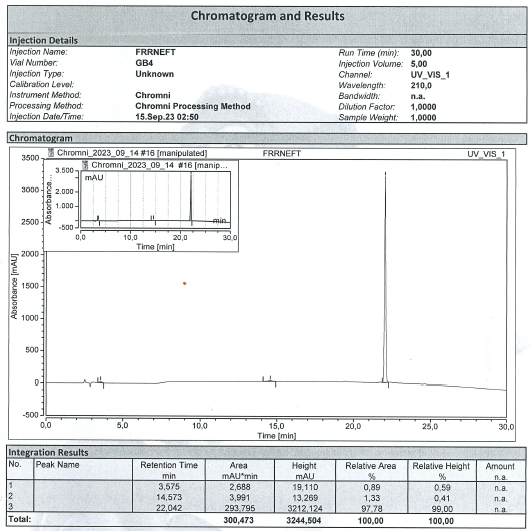


HPLC chromatogram of **14** showing the purity of **14** (t_R_ = 22.0 min, method 1).


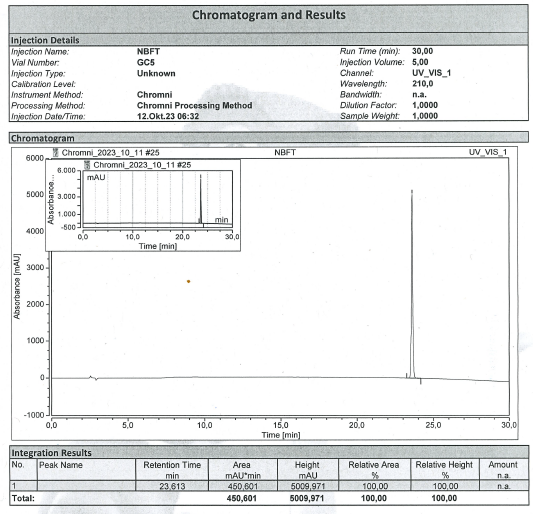


HPLC chromatogram of **15** showing the purity of **15** (t_R_ = 23.6 min, method 1).


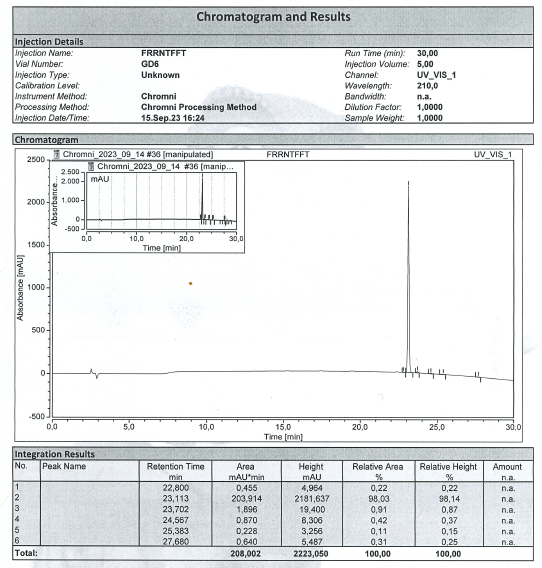


HPLC chromatogram of **16** showing the purity of **16** (t_R_ = 23.1 min, method 1).


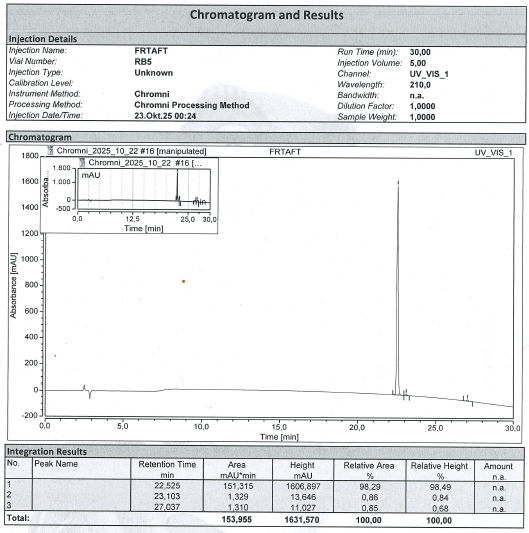


HPLC chromatogram of *rac-***17** showing the purity of *rac-****17*** (t_R_ = 22.5 min, method 1).


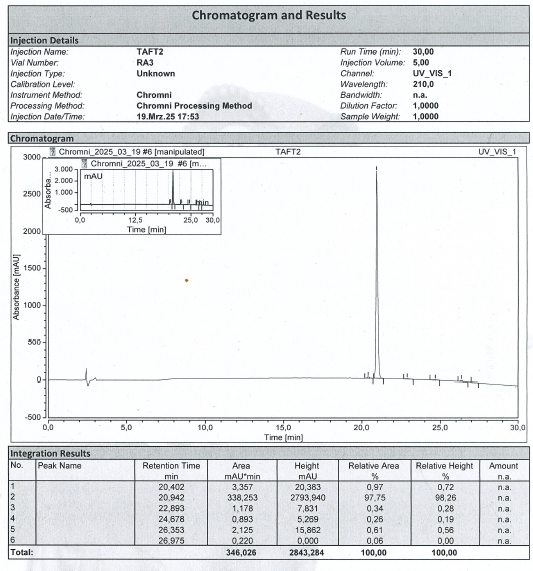


HPLC chromatogram of *(R)*-**17** showing the purity of *(R)-***17** (t_R_ = 20.9 min, method 1).


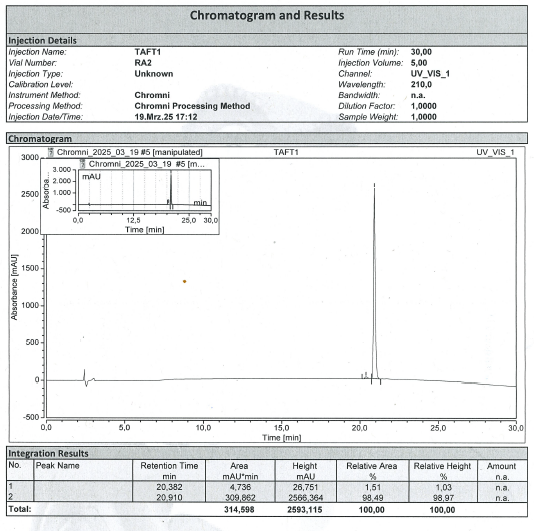


HPLC chromatogram of *(S)-***17** showing the purity of *(S)-***17** (t_R_ = 20.9 min, method 1).


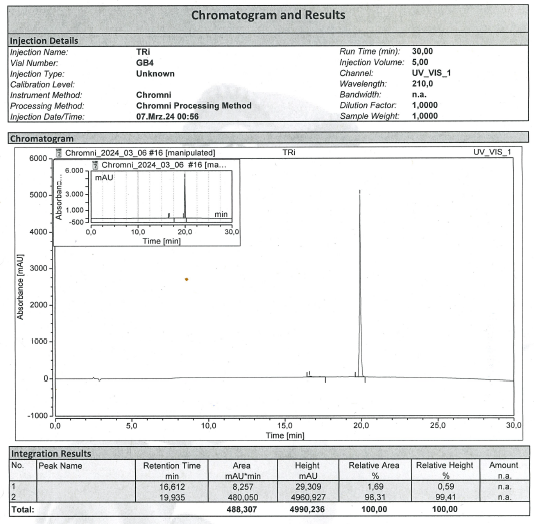


HPLC chromatogram of *rac-***18** showing the purity of *rac-***18** (t_R_ = 19.9 min, method 1).


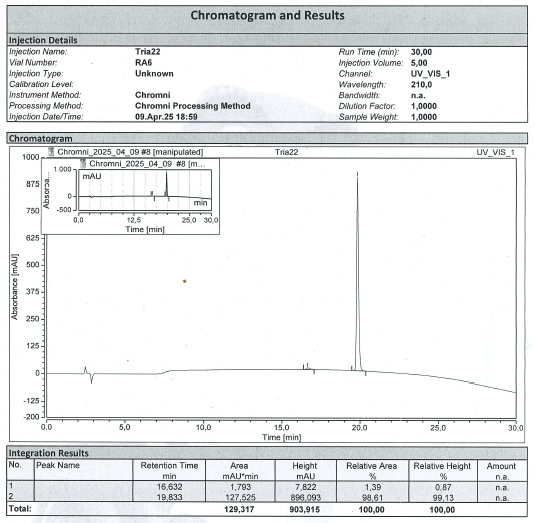


HPLC chromatogram of *(R)-***18** showing the purity of *(R)-***18** (t_R_ = 19.8 min, method 1).


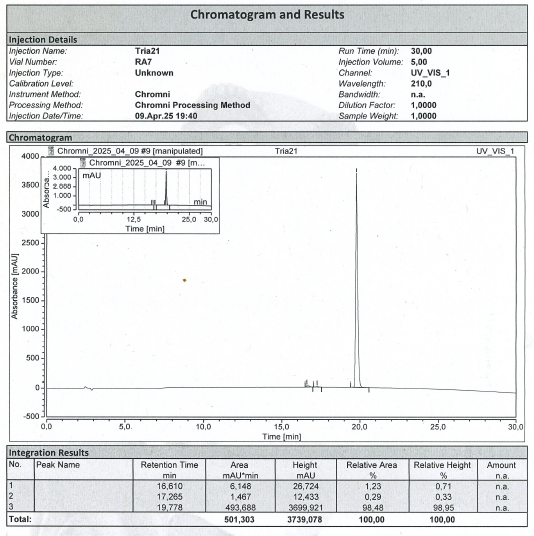


HPLC chromatogram of *(S)-***18** showing the purity of *(S)*-**18** (t_R_ = 19.8 min, method 1).
